# Regioselective Biocatalytic C4‐Prenylation of Unprotected Tryptophan Derivatives

**DOI:** 10.1002/cbic.202200311

**Published:** 2022-07-13

**Authors:** Bettina Eggbauer, Joerg H. Schrittwieser, Bianca Kerschbaumer, Peter Macheroux, Wolfgang Kroutil

**Affiliations:** ^1^ Institute of Chemistry University of Graz NAWI Graz Heinrichstraße 28 8010 Graz Austria; ^2^ Institute of Biochemistry Graz University of Technology Petersgasse 10–12 8010 Graz Austria; ^3^ BioTechMed Graz 8010 Graz Austria; ^4^ Field of Excellence BioHealth University of Graz 8010 Graz Austria

**Keywords:** biocatalysis, C−C bond formation, prenylation, protein engineering, regioselectivity

## Abstract

Regioselective carbon−carbon bond formation belongs to the challenging tasks in organic synthesis. In this context, C−C bond formation catalyzed by 4‐dimethylallyltryptophan synthases (4‐DMATSs) represents a possible tool to regioselectively synthesize C4‐prenylated indole derivatives without site‐specific preactivation and circumventing the need of protection groups as used in chemical synthetic approaches. In this study, a toolbox of 4‐DMATSs to produce a set of 4‐dimethylallyl tryptophan and indole derivatives was identified. Using three wild‐type enzymes as well as variants, various C5‐substituted tryptophan derivatives as well as *N*‐methyl tryptophan were successfully prenylated with conversions up to 90 %. Even truncated tryptophan derivatives like tryptamine and 3‐indole propanoic acid were regioselectively prenylated in position C4. The acceptance of C5‐substituted tryptophan derivatives was improved up to 5‐fold by generating variants (e. g. T108S). The feasibility of semi‐preparative prenylation of selected tryptophan derivatives was successfully demonstrated on 100 mg scale at 15 mM substrate concentration, allowing to reduce the previously published multistep chemical synthetic sequence to just a single step.

## Graphical abstract

**C4‐regioselective prenylation** of 5‐substituted tryptophans as well as truncated tryptophan derivatives was achieved using a set of prenlyltransferases. Conversions were increased by designing improved variants and the feasibility of preparative transformations was successfully demonstrated reaching isolated yields up to 90 %.

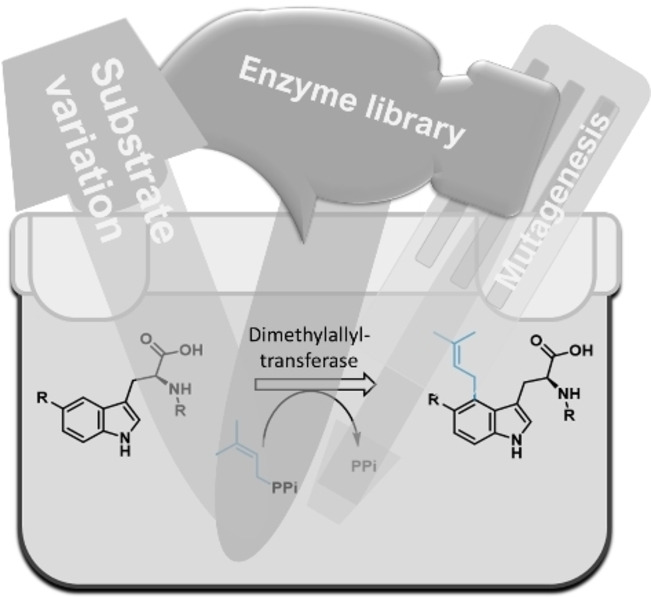

## Introduction

Carbon−carbon bond formation presents a key step in organic chemistry to build the carbon frameworks of molecules. With the goal to create complex structures from smaller, readily available building blocks, numerous strategies for C−C bond formation have been developed, like traditional methods or advanced approaches of organocatalysis and transition metal catalysis.^[1]^ In recent years, the scope of biocatalytic methods for C−C bond formation has been expanded,^[2]^ with recent examples on aldolases, Pictet‐Spenglerases, and engineered cytochromes P450 that catalyze cyclopropanation.^[3]^

Another group of less investigated C−C bond forming enzymes are prenyltransferases.^[4]^ They play an important role in nature for the diversification of natural products by regioselectively attaching prenyl groups to aromatic structures.^[5]^ They catalyze the coupling of dimethylallylpyrophosphate (DMAPP) and selected derivatives to various aromatic structures, *e. g*., flavonoids, and amino acids like tryptophan and tryptophan derivatives.^[6]^

In view of the central role of tryptophan in the biosynthesis of neurotransmitters and secondary metabolites,^[7]^ the diversification of tryptophan represents an important branching point in biosynthesis.^[8]^ Biologically active compounds derived from tryptophan include indole alkaloids, like for instance ergoline alkaloids or communesin B, a fungal natural product possessing anti‐cancer activity as well as the hormones serotonin and melatonin.^[9]^

**Scheme 1:**
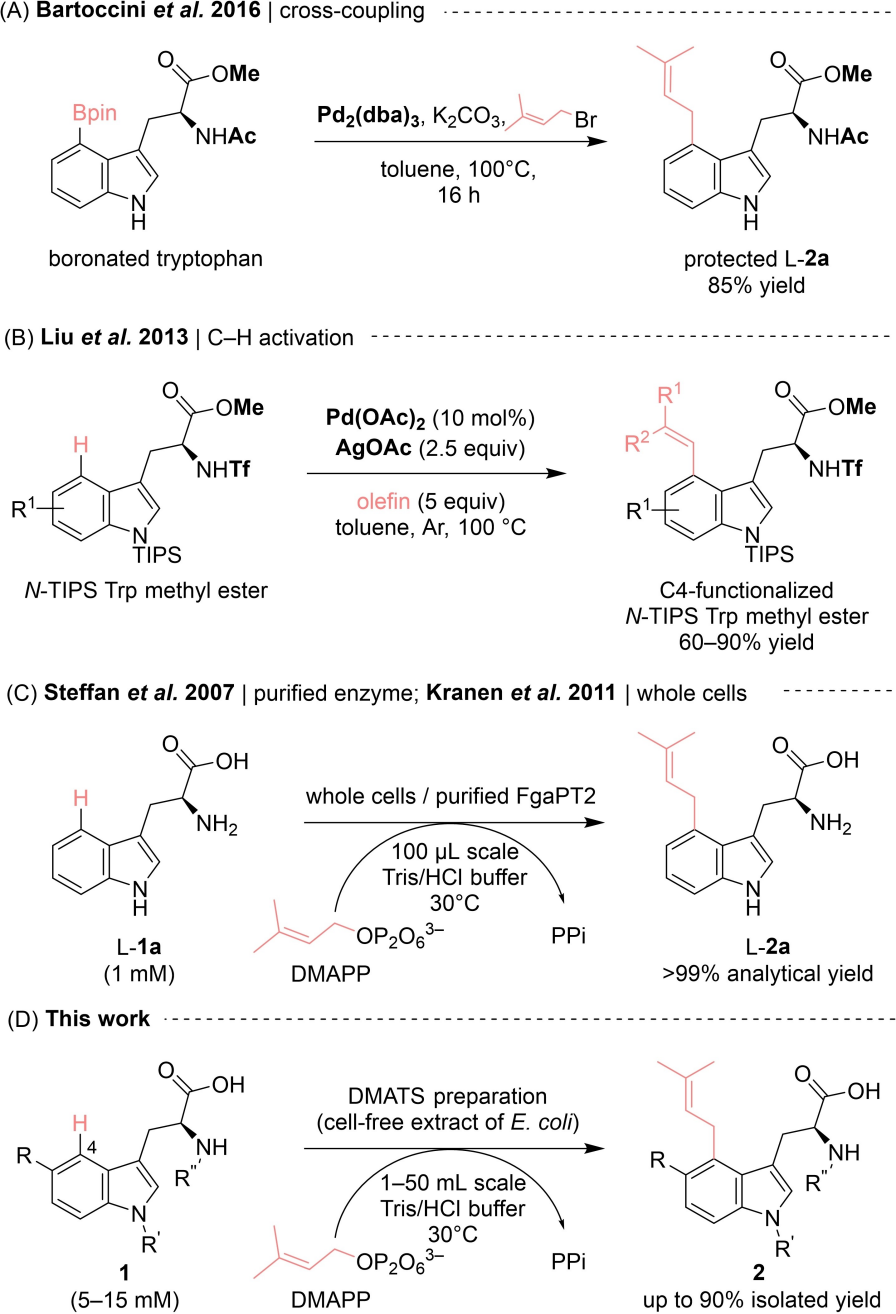
Transition metal catalyzed and biocatalytic approaches to L–DMAT (L‐**2 a**) and derivatives.

A wide range of tryptophan derivatives has already been targeted by chemical but also chemo‐enzymatic synthetic routes, whereby the main challenge being the selective substitution at the C4 position of the indole ring. Approaches employing for example palladium^[10]^ or iridium based catalysts^[11]^ were predominantly regioselective for the C2, C3, and C5 sites and to a lesser extent for C6 and C7, whereas selective functionalization at position C4 was rarely achieved.[^11c^, ^12^] In a recent synthesis of C4‐substituted tryptophan derivatives, a C4‐preactivated substrate (4‐boronated *N*‐acetyl‐tryptophan methyl ester) was prenylated via palladium‐catalyzed cross‐coupling (Scheme 1A).^[13]^ Another transition metal (Pd) catalyzed approach to C4‐substituted tryptophan derivatives required directing and protecting groups for direct olefination (Scheme 1B).^[14]^ Although these synthetic methods achieved high regioselectivity and fair yields, they relied on protected tryptophan derivatives whose preparation required multiple steps.^[14]^ Therefore, the direct prenylation of unprotected L‐tryptophan using prenyltransferases would present a convenient alternative to the above‐mentioned chemical routes (Scheme 1C, D).

Fungal 4‐dimethylallyl tryptophan synthase (4‐DMATS)‐type prenyltransferases catalyze the C4‐selective prenylation of L‐tryptophan (**1 a**) with dimethylallylpyrophosphate (DMAPP) to give 4‐(dimethylallyl)tryptophan (**2 a**, 4‐DMAT) and pyrophosphate. This reaction constitutes the first step in the alkaloid biosynthesis pathway of ergot fungi, which produce a great diversity of ergot alkaloids from the classes lysergic acid and derivatives, clavines or ergopeptines.^[15]^ 4‐DMATS‐encoding genes (termed DmaW or FgaPT2) identified in *Claviceps purpurea*, *Aspergillus japonicus* and *Aspergillus fumigatus* have been cloned and heterologously expressed in *Saccharomyces cerevisiae*.^[16]^ Also diprenylated indole derivatives have been synthesized with recombinant DMATS.^[17]^ However, initial reports on the acceptor and donor substrate scope for C4‐prenylation have been performed for purified FgaPT2 or FgaPT2 displayed on the surface of *E. coli* cells[^5a^, ^16a^, ^18^] and were performed on a small scale (100 μL–1 mL) with low substrate loadings (max. 1 mM). It is worth to mention, that prenylation of C4‐substituted tryptophan analogues was reported in other positions with this type of enzyme on a 5 mL scale at 20 mM tryptophan/8.8 mM DMAPP.^[19]^ During the last years, various DMATS have been investigated for the prenylation of several alternative substrates like fumiquinazolines^[20]^ and tyrosines.^[21]^

Herein, we describe the regioselective biocatalytic C4‐prenylation of L‐tryptophan and derivatives with the aim to (i) to extend the library of 4‐DMATSs, (ii) to investigate the window of reaction parameters, (iii) to elucidate the scope and limitations with respect to the substrate pattern, and (iv) to improve the substrate scope by using rational protein engineering for the 4‐DMATS from *A. japonicus*. As prenylated tryptophan serves as key intermediate to various alkaloids,^[8]^ a scalable method and the access to new derivatives may open the door towards new alkaloid derivatives with new properties. Compared to previously reported prenylations catalyzed by DMATS from *A. fumigatus* and *Cl. purpurea*, the one from *A. japonicus* is less explored. For this reason, we put it in the focus.

## Results and Discussion

### Prenylation of L‐tryptophan by DMATS from *A. japonicus*

**Figure 1:**
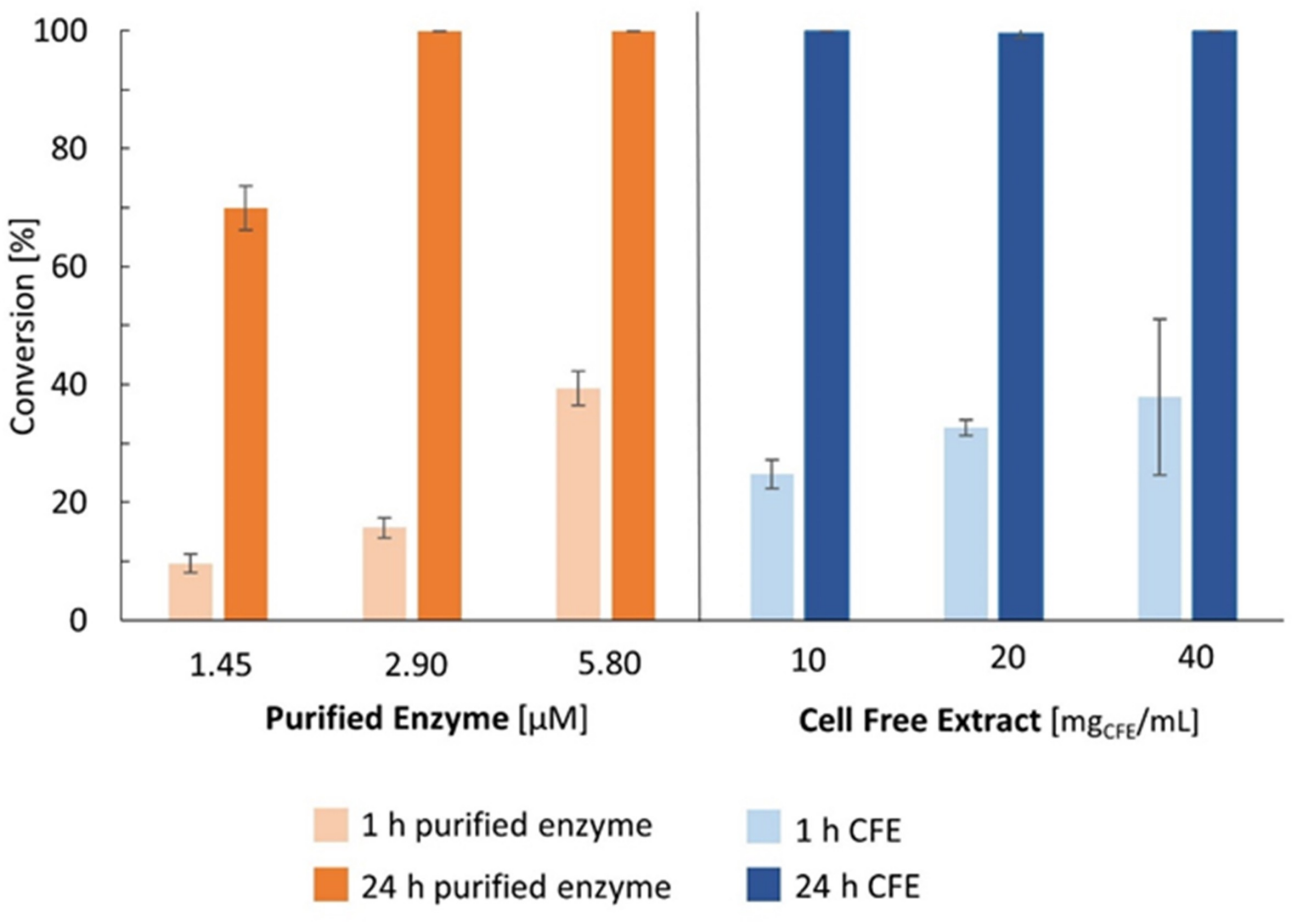
Comparison of prenylation of L**‐1 a** using varied amounts of the prenyltransferase DmaW either as purified preparation (orange) or cell‐free extract (blue). Results after 1 h are shown as light bars and those after 24 h as dark bars. Reactions were investigated using three concentrations of purified DmaW from *A. japonicus* (100, 200 and 400 μg/mL corresponds to 1.45, 2.9 and 5.8 μM) and three concentrations of CFE DmaW preparation [CFE of *E. coli* BL21(DE3), 10, 20 and 40 mg_CFE_/mL of lyophilized CFE corresponding to 6.5, 13 and 26 μM pure DmaW]. Reaction conditions: L‐**1 a** (1 mM), DMAPP (2 mM), Tris/HCl buffer (50 mM pH 7.5, 5 mM CaCl_2_), 30 °C, 24 h.

Based on a literature reported enzymatic C4‐prenylation,^[18d]^ the natural substrate L‐tryptophan (L‐**1 a**, 1 mM) was initially transformed with purified prenyltransferase from *A. japonicus* (DmaW, *Aj*‐4‐DMATS) at varied enzyme concentrations (1.45–5.8 μM) giving 10–39 % conversion within 60 min (Figure 1, light‐orange bars) and quantitative conversion within 24 h when using an enzyme concentration of at least 2.9 μM (dark‐orange bars). Since for synthetic *in‐vitro* applications the use of a cell‐free extract (CFE) preparation is favored over a purified enzyme (especially in case of expression in *E. coli* the laborious purification can be avoided as well as the reagents required for purification), the prenylation was performed using a DmaW−CFE preparation allowing quantitative conversion of C4‐prenylated tryptophan L‐**2 a** within 24 h at 1 mM substrate concentration (Figure 1, dark‐blue bars). The amount of DmaW present in the CFE was estimated to be 0.65 nmol per mg of CFE based on initial rate measurements (Figure S3). It is worth noting that CFE contains already some L‐**1 a** corresponding to 0.25 mM when using 20 mg_CFE_/mL (Supporting Information). Stopping the prenylation reaction already after 60 min showed comparable results for 20 and 40 mg_CFE_/mL, corresponding to 13 and 26 μM pure DmaW (light‐blue bars). Consequently, further biotransformations were conducted with 20 mg_CFE_/mL to keep the amount of catalyst applied to a minimum.

**Figure 2:**
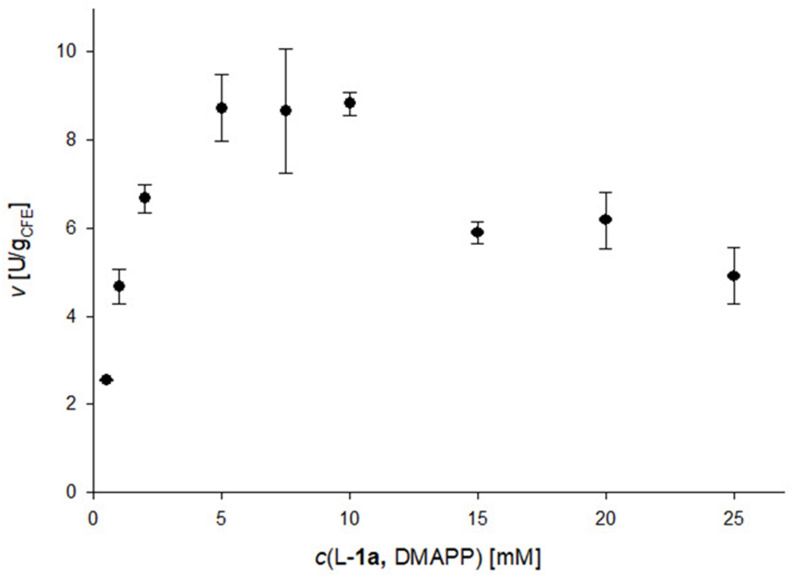
Reaction rate at increasing L‐**1 a**/DMAPP concentration at a 1 : 1 ratio of the two reagents. Reaction conditions: L‐**1 a (**0.5–25 mM), DMAPP (0.5–25 mM), DmaW preparation [CFE of *E. coli* BL21(DE3), 20 mg_CFE_/mL ≙ 13 μM pure enzyme], Tris/HCl buffer (50 mM, pH 7.5, 5 mM CaCl_2_)_,_ DMSO (5 % v/v), 30 °C, 30 min.

In a subsequent experiment it was shown that a 1 : 1 ratio of the two reactants L‐**1 a** and DMAPP is sufficient at 1 mM L‐**1 a** to reach completion (Figure S4), thus DMAPP does not need to be in excess to allow all substrate L‐**1 a** to be converted. Since previously only low substrate concentrations (maximum 1 mM) have been reported,[^18a^, ^18d^] the activity at increasing concentration of the two substrates was investigated (Figure 2). Varying the concentration of L‐**1 a** and DMAPP at a ratio of 1 : 1 between 0.5–25 mM, the highest rates were observed with 5.0–10.0 mM of L‐**1 a**/DMAPP with 8.7–8.8 U/g_CFE_. An increase to 25 mM caused a decrease of rate to 4.9 U/g_CFE_, indicating substrate and/or product inhibition and suggesting an optimal substrate concentration of 10.0 mM for batch reactions.

**Figure 3:**
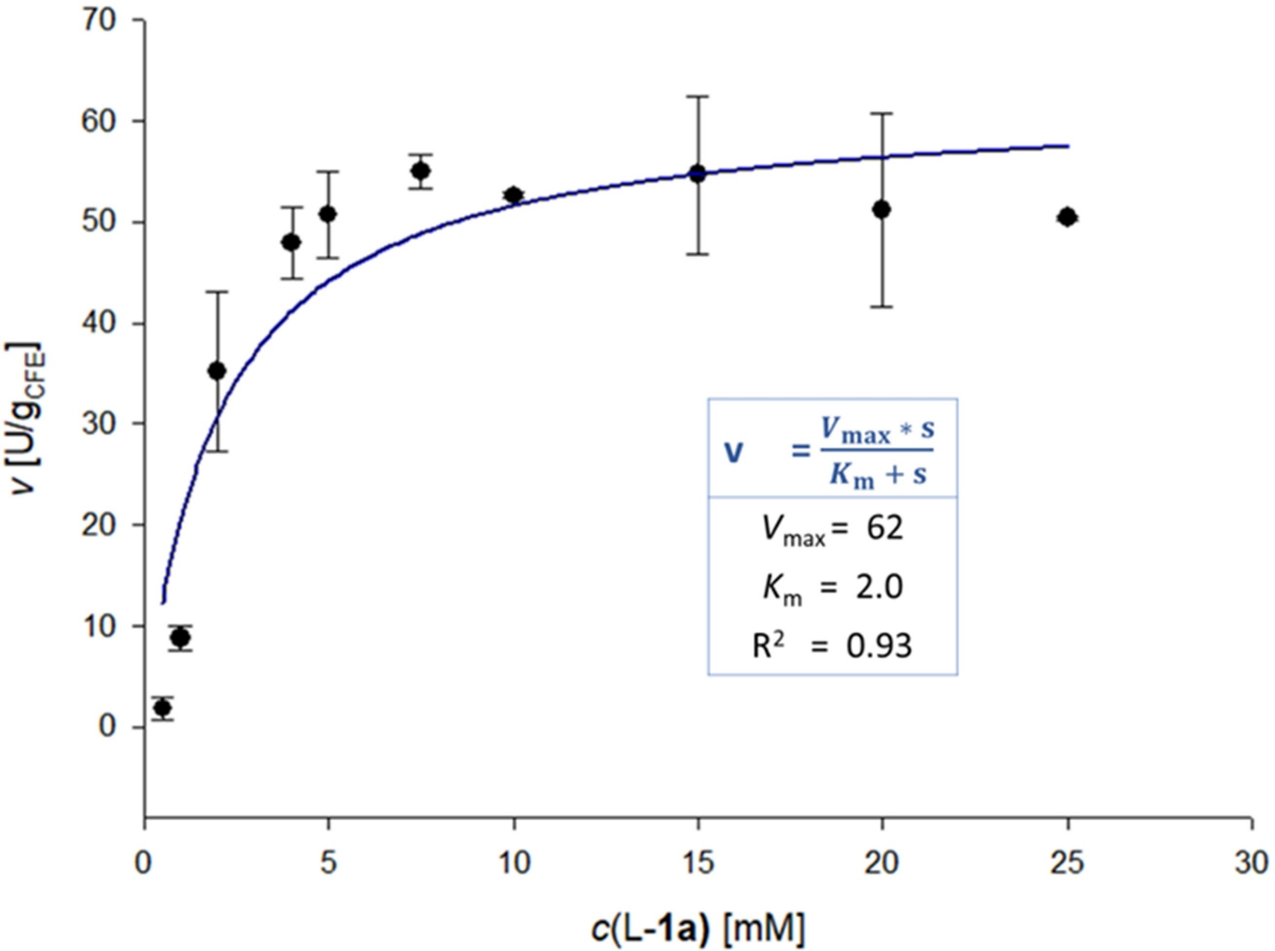
Reaction rate vs. L‐**1 a** concentration at 5 mM DMAPP. Mean values of triplicate measurements (black dots; error bars indicate standard deviation) were fitted to the Michaelis‐Menten equation (blue line) by non‐linear regression. The fit function and the determined kinetic parameters are shown in the light‐blue box. Reaction conditions: L‐**1 a (**0.5–25 mM), DMAPP (5 mM), DmaW preparation [CFE of *E. coli* BL21(DE3), 5 mg_CFE_/mL ≙ 3.25 μM], Tris/HCl buffer (50 mM, pH 7.5, 5 mM CaCl_2_)_,_ DMSO (5 % v/v), 30 °C, 30 min.

**Figure 4:**
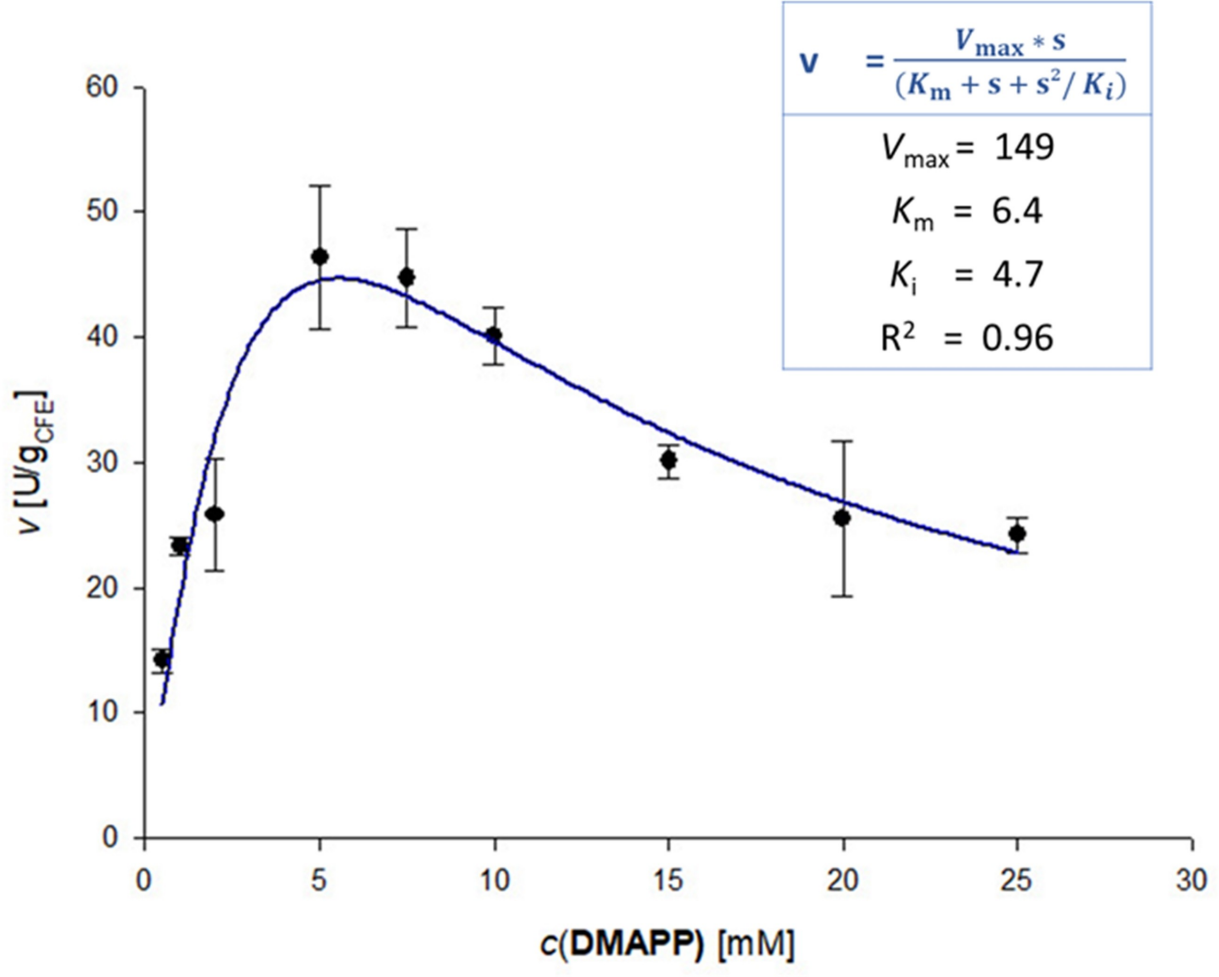
Reaction rate vs. DMAPP concentration at 5 mM L**‐1 a** indicating substrate inhibition. Mean values of triplicate measurements (black dots; error bars indicate standard deviation) were fitted to the Michaelis‐Menten equation for uncompetitive substrate inhibition (blue line) by non‐linear regression. The fit function and the determined kinetic parameters are shown in the light‐blue box. Reaction conditions: L**‐1 a** (5 mM), DMAPP (0.5–25 mM), DmaW preparation [CFE of *E. coli* BL21(DE3), 5 mg_CFE_/mL ≙ 3.25 μM], Tris/HCl buffer (50 mM, pH 7.5, 5 mM CaCl_2_)_,_ DMSO (5 % v/v), 30 °C, 30 min.

To elucidate whether there is substrate inhibition caused by both substrates or just by one, the kinetics were determined by varying one substrate concentration while keeping the other constant (Figures 3 and 4). These experiments revealed that only DMAPP caused substrate inhibition for DmaW (*K*_i_=4.7 mM), whereas the kinetics for L‐**1 a** were well described by the Michaelis‐Menten model between 0.5–25 mM (Table 1). In literature the *K*_M_ for L‐**1 a** varies between 7 μM and 0.2 mM for the *A. fumigatus* enzyme,^[22]^ whereby the DMAPP concentration was just kept between 200 μM and 1 mM. The *K*_M_ for DMAPP varies between 4 μM^[16a]^ and 52 μM^[23]^ at 1 mM L‐**1 a**. An inhibition by DMAPP has never been reported before, which might be due to the low concentrations investigated.

**Table 1:** Apparent kinetic parameters of DmaW for prenylation of L‐**1 a** with DMAPP.

Substrate	*K*_M_^[a]^ [mM]	*V*_max_^[a]^ [U/g_CFE_]	*V*_max_^[a]^ [U/mg_pure enzyme_]
DMAPP (5 mM L‐**1 a**)	6.9±1.2 (*K*_i_: 4.7±0.4 mM)	149±20	3.3±0.4
L‐**1 a** (5 mM DMAPP)	2.0±0.8	62±5.8	1.4±0.1

[a] Reaction conditions: L‐**1 a** (5 mM or 0.5–25 mM), DMAPP (5 mM or 0.5–25 mM), DmaW preparation [CFE of *E. coli* BL21(DE3), 5 mg_CFE_/mL ≙ 3.25 μM enzyme], Tris/HCl buffer (50 mM, pH 7.5, 5 mM CaCl_2_), DMSO (5 % v/v), ambient atmosphere, 30 °C, 0–15 min, reaction volume: 1 mL.

Testing for product inhibition by L**‐2 a**, prenylation experiments of L**‐1 a** (5 mM) in the presence of L**‐2 a** (Figure S6) led to quantitative conversion of L**‐1 a** within 60 min with up to 5 mM of L**‐2 a** supplementation, whereas 10 mM and 20 mM of L**‐2 a** led to slightly lower conversion (90 % and 75 %, respectively). Consequently, for substrate concentrations up to 10 mM, product inhibition by L**‐2 a** was not considered to be relevant. Product inhibition by formed pyrophosphate (PP_i_) could be excluded, due to endogenous inorganic pyrophosphatase present in *E. coli* CFE, a specific enzyme hydrolyzing PP_i_ to P_i_.^[24]^

As **1 a** has only low solubility in water (≈55 mM at 25 °C),^[25]^ co‐solvents such as MeOH, EtOH, acetonitrile, 2‐propanol, DMF, and DMSO were considered to improve the solubility, whereby only DMSO allowed dissolving 100 mM of L**‐1 a**. Testing the activity at increasing DMSO concentration in buffer up to 25 % v/v (Figure S5) showed comparable results for 5 % v/v DMSO and in the absence of DMSO, while at higher DMSO concentrations the rates were reduced; subsequently, 5 % v/v DMSO was used for further experiments, especially as the solubility for selected substituted derivatives (*e. g*., **1 g**) was even worse.

### Prenylation of non‐natural substrates

**Scheme 2:**
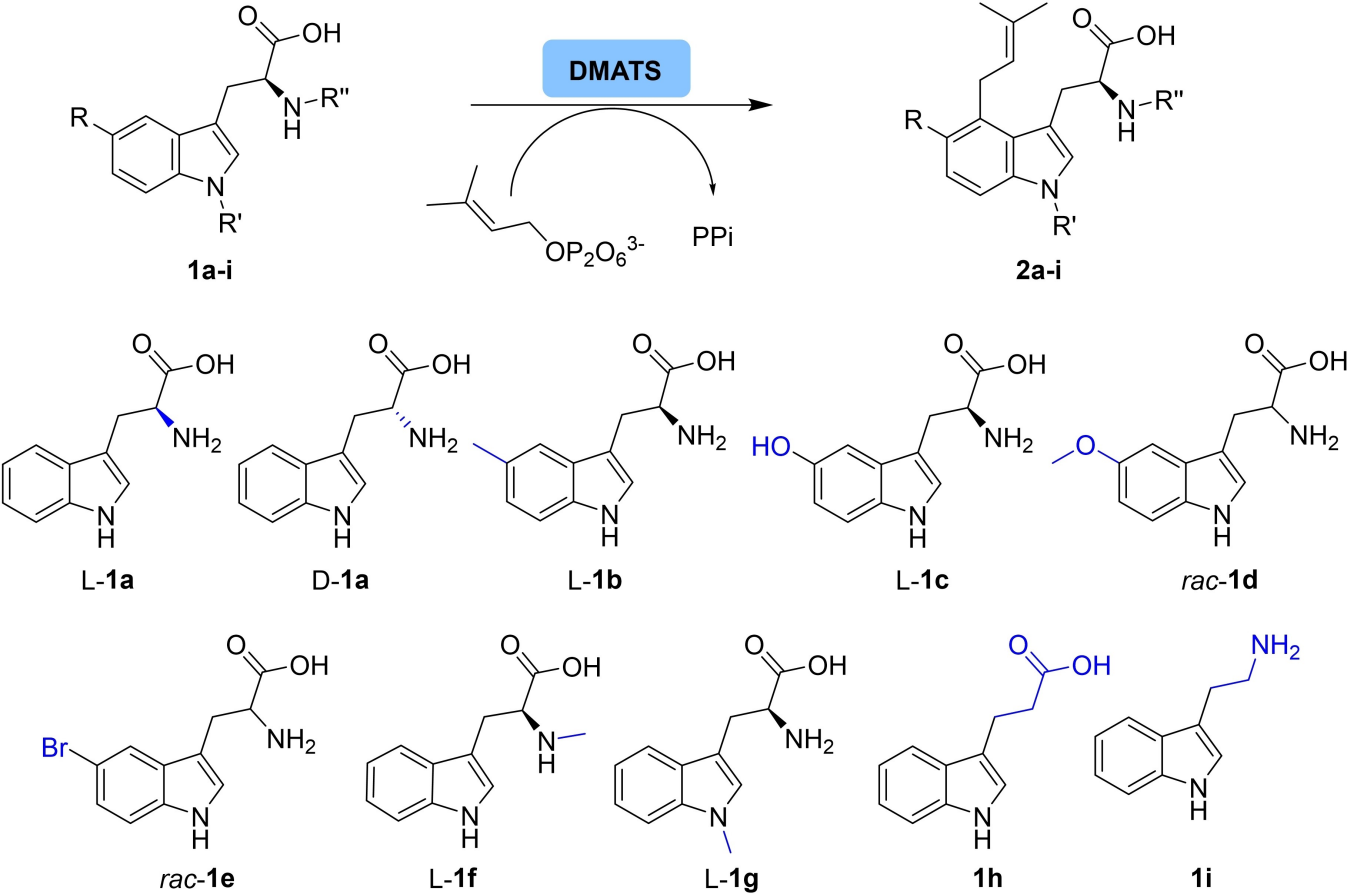
Biocatalytic prenylation of tryptophan derivatives **1 a**–**i**.

Having identified a suitable substrate and solvent concentration range for the C4‐prenylation of L**‐1 a** by dimethylallyltransferase from *A. japonicus* (DmaW, *Af*‐4‐DMATS), substituted tryptophan derivatives (**1 b**–**1 g**) as well as truncated tryptophan derivatives (**1 h**, **1 i**) and D**‐1 a** were investigated (Scheme 2).

Although one might expect a high stereo‐recognition by the enzyme for a stereogenic center as present in L**‐1 a**, the mirror‐image substrate D**‐1 a** was prenylated with reasonable conversion (20 %) at 5 mM substrate concentration within 24 hours (Table 2). When comparing the rates, L**‐1 a** was transformed just three times faster than its mirror image D**‐1 a** (Figure S5), opening the possibility to C4‐prenylate both enantiomers of **1 a**.

**Table 2:** Prenylation of tryptophan and derivatives **1 a**–**1 i** by DMATSs from *A. japonicus*, *Trichophyton benhamiae* and *C. purpurea*.

Substrate	Conversion^[a]^ [%]		
	DMATS from *A. japonicus*	DMATS from *C. purpurea*	DMATS from *T. benhamiae*
L**‐1 a**	>99	8	39
D**‐1 a**	20	n.d.	n.d.
L**‐1 b**	3.9	3.5	12
L**‐1 c**	24	60	29
*rac* **‐1 d**	10^[c]^	22^[c]^	24^[c]^
*rac* **‐1 e**	23^[c]^	11^[c]^	13^[c]^
L**‐1 f**	90^[b]^	31^[b]^	65^[b]^
L**‐1 g**	11	48	20
**1 h**	17	5	4
**1 i**	53	52	44

[a] Conversion (consumption of substrate **1**) determined by HPLC‐UV (262 nm) on an achiral stationary reversed‐phase using propiophenone (2 mM) as internal standard. The reported values are mean of three independent experiments. n.d.=no product detected. Reaction conditions: tryptophan derivative (5 mM), DMAPP (5 mM), DMATS preparation [CFE of *E. coli* BL21(DE3), 20 mg_CFE_/mL], Tris/HCl buffer (50 mM, pH 7.5, 5 mM CaCl_2_), DMSO (5 % v/v), under ambient atmosphere, 30 °C, 24 h, reaction volume: 1 mL. [b] In contrast to all other substrates, the transformation of L‐**1 f** led to several side products beside the desired product L‐**2 f**, such as L‐**2 a** but also **3 f** and **4 f**. The reaction mixture contained for: *A. j*.: 35 % L‐**2 a**, 15 % L‐**2 f**, 5 % **3 f**, 35 % **4 f**; *C. p*.: 6.8 % L‐**2 a**, <1 % L‐**2 f**, <1 % **3 f**, 24 % **4 f**; *T. b*.: 23 % L‐**2 a**, <1 % L‐**2 f**, <1 % **3 f**, 49 % **4 f**. [c] The *e.e*. of the remaining substrate was in general below 5 % with D‐**1** in excess. The *e.e*. was determined by HPLC‐MS on a chiral stationary phase.

As the position at C5 of the indole moiety might cause steric challenges for the reaction due to the proximity to the reaction center C4 and is therefore the most demanding position, several small substituents at C5 were investigated (**1 b**–**1 e**). Interestingly, the wild‐type DMATS from *A. japonicus* transformed the derivative L**‐1 b** possessing a methyl group at C5 only very poorly (4 % conv.), but an OH group was accepted reasonably well (24 % conv.). Even the bigger methoxy group as well as the bromo substituent were accepted similar as the OH group (substrates *rac*‐**1 d**, *rac*‐**1 e**).

To expand the number of suitable DMATSs for C4‐prenylation, a BLAST search was performed using the amino acid sequence of DmaW from *A. japonicus* as query sequence. This search returned 193 hits with an identity of >55 % to the sequence from *A. japonicus* (Table S5). Based on the BLAST search, four DMATSs [*Aspergillus fumigatus* (KEY80409.1)*, Malbranchea aurantiaca* (ABZ80611.1), *Claviceps purpurea* (CAC37397.1) and *Trichophyton benhamiae* (XP_003017766.1)] from fungi were arbitrary selected, whereby the enzymes were chosen to be from different organisms and also display different similarities (68, 64 and 57 %). The enzymes were expressed using synthetic codon‐optimized genes. After successful soluble expression of all four candidates, two of them showed increased activity for some tryptophan derivatives, namely the DMATS from *Claviceps purpurea* ^[26]^ and from *Trichophyton benhamiae*, possessing identities of 57 % and 69 % with DmaW, respectively. Interestingly, the 4‐DMATS from *C. purpurea* and *T. benhamiae* did not convert D**‐1 a** at all, only L**‐1 a** was converted indicating that these enzymes are stereoselective, and thus, they are complementary to DmaW. Interestingly, all wildtype enzymes converted the 5‐methyl substrate L**‐1 b** with lower conversion than the subsequent apparently more sterically demanding substrates. Furthermore, the 4‐DMATS from *C. purpurea* showed higher conversion for the C5‐hydroxy substituted derivative L**‐1 c** than the one from *A. japonicus*.

Comparing the three active 4‐DMATSs by amino acid sequence alignment (Benchling multi‐sequence alignment tool, Figure S9), revealed that the active site regions are conserved. All three DMATSs possess the catalytically important residues Lys174 and Glu85 (numbering according to *C. purpurea*), which were previously assigned^[27]^ to abstract a proton from the σ‐complex at C4 and to interact with the indole N−H. The sequence alignment also confirmed strict conservation of most residues involved in coordinating the pyrophosphate moiety (*i. e*., K186, Y188, R262, K264, Y408, Y414; numbering according to *C. purpurea*) in all the mentioned enzymes. Additionally, four tyrosine residues, namely Y266 and Y350 as well as Y185 and Y414, suggested to protect the reactive carbocation from water,[^27^, ^28^] were identified in all three DMATSs. Moreover, four residues (I80, L81, Y190 and R249) that anchor the polar side chain of the substrate L**‐1 a** were conserved in all three enzymes, consistent with their common regioselectivity towards the C4 position.^[27]^

**Scheme 3:**
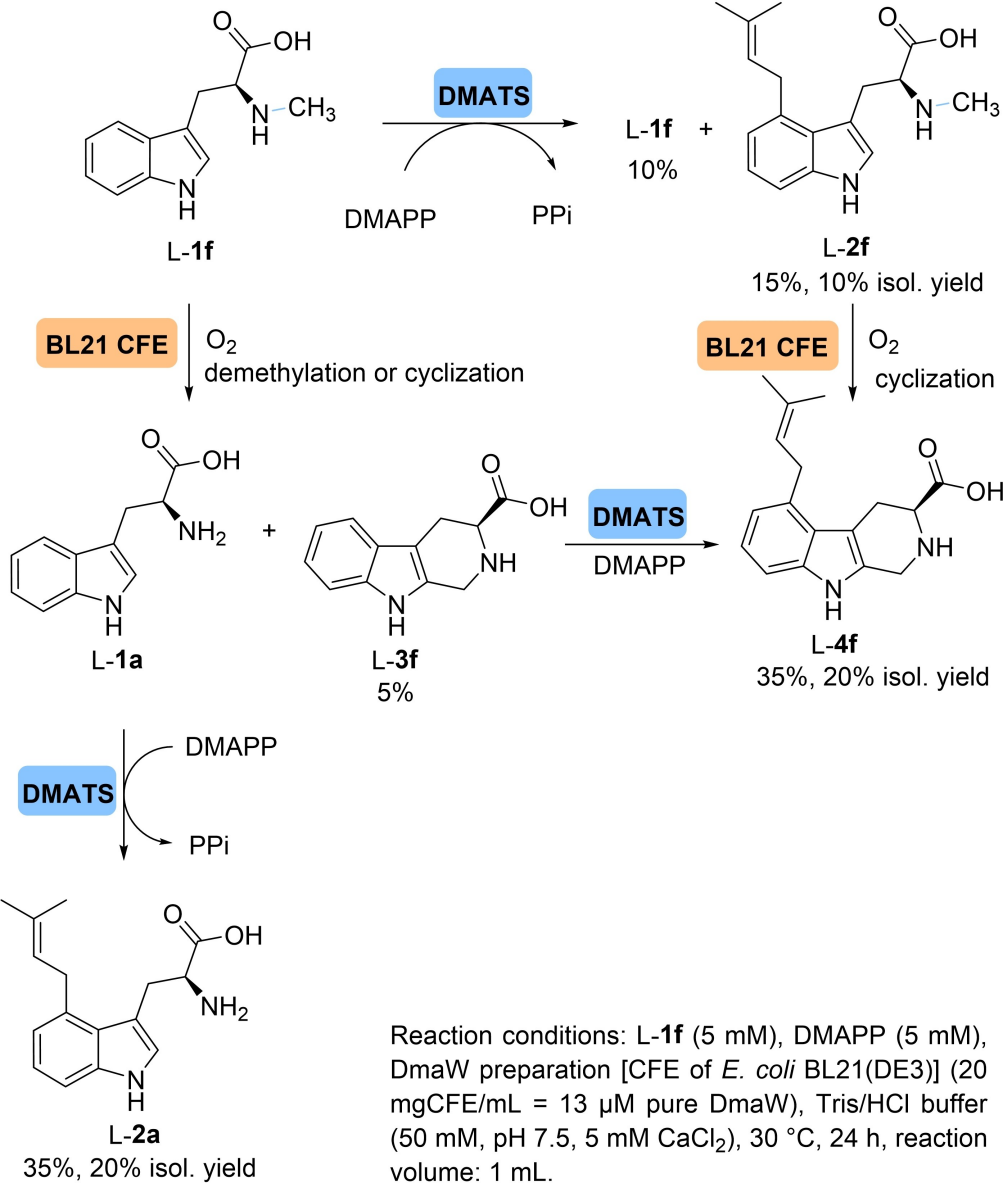
Possible reaction pathways transforming L‐abrine (**1 f**) via prenylation, demethylation and cyclization leading to products L‐**2 a**, L‐**3 f** and L‐**4 f** using cell‐free extract of *E. coli* BL21(DE3)/DMATS.

Next, we investigated the conversion of two *N*‐methylated derivatives of indole (L**‐1 f** and L**‐1 g**). It turned out that both were converted, whereby the transformation of L‐abrine (**1 f**) with DMAPP afforded unexpected products when using CFE (Scheme 3). Besides the expected prenylated product (L**‐2 f**), two additional products were identified after preparative‐scale biotransformation using *Aj*‐DMATS (100 mg L**‐1 f**, 15 mM). At a conversion of 90 % after 24 h, L**‐2 f** was obtained in 10 % isolated yield and the two additional products with 20 % isolated yield each. Analysis of the new products revealed the formation of prenylated demethylated tryptophan L**‐2 a** and a prenylated cyclization product L**‐4 f**. Further analysis revealed that the demethylation/cyclization is not caused by DMATS but due to activities present in the CFE of *E. coli* BL21(DE3) (Supporting Information, “Characterization of L‐abrine prenylation by DmaW”). The same side products were also observed with CFE preparations of DMATS from *C. purpurea* and *T. benhamiae*. Since it has previously been described that *E. coli* possesses a *N*‐methyltryptophan oxidase (MTOX),^[29]^ which was reported to afford the oxidative demethylation of *N*‐methyltryptophan/L‐abrine (**1 f**),^[30]^ we ascribe the generation of the demethylated product to the activity of this enzyme present in the crude cell‐free extract. Interestingly, cyclisation has not been reported for MTOX yet, although other members of this enzyme family are known to catalyze oxidative cyclisation reactions.^[31]^ Consequently, the observed cyclisation may be expected to occur due to the presence of MTOX in the CFE. This initial observation may trigger further investigations with the aim to elucidate the interplay of MTOX and DMATS in generating novel indole derivatives from *N*‐methyltryptophan and derivatives thereof.

**Figure 5:**
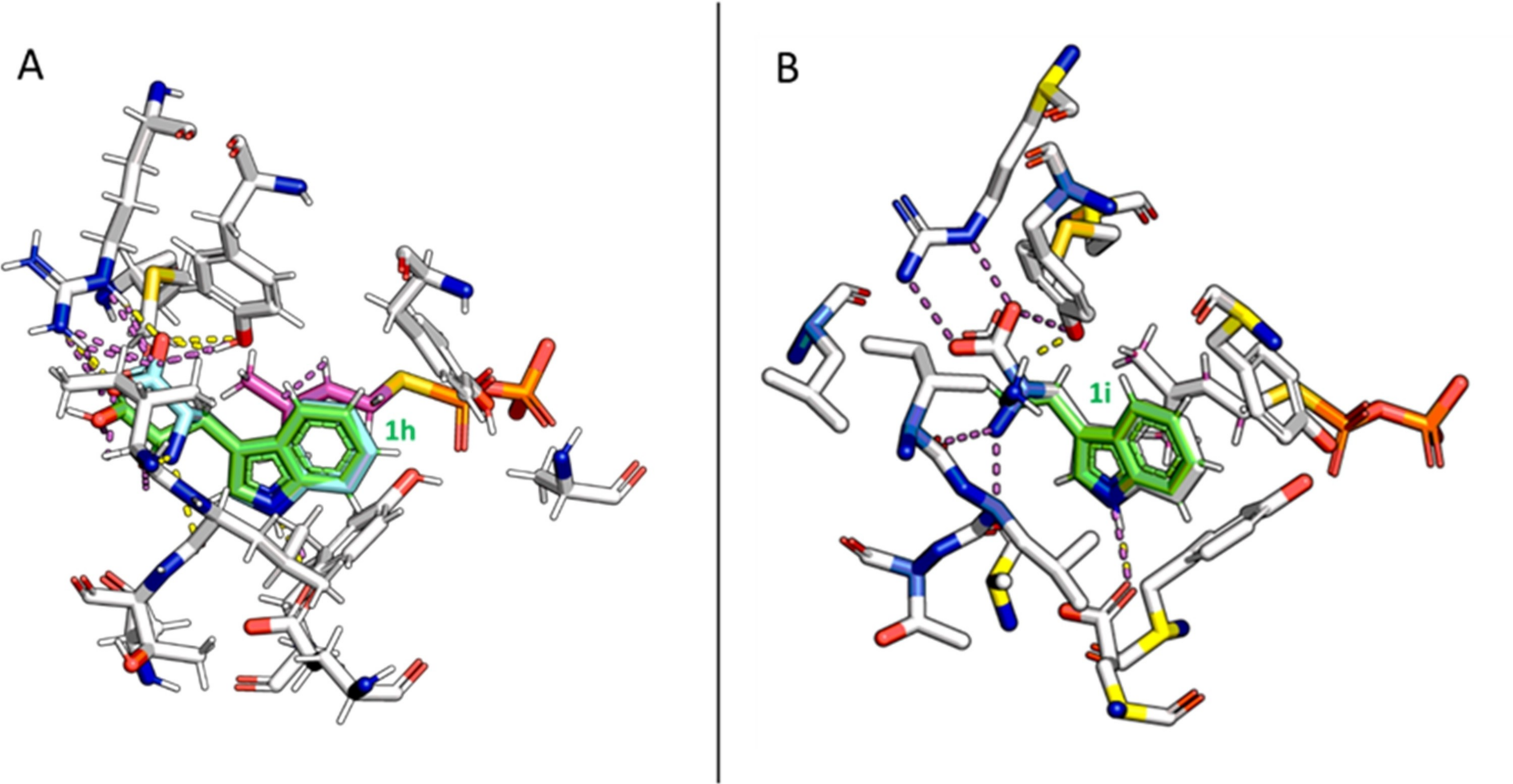
Structural alignment of docking poses for L‐**1 a** (turquoise) with (A) **1 h** (green) and (B) **1 i** (green) in the active site of DMATS from *A. fumigatus*. Polar interactions of L**‐1 a** are highlighted in magenta and polar interactions of indole derivatives in yellow. The X‐ray structure of *A. fumigatus* was used since the predicted structure of the DMATS from *A. japonicus* using AlphaFold2^[32]^ aligned perfectly with the experimentally determined structure of *A. fumigatus*.

Finally, moving to tryptophan derivatives lacking either the α‐amino moiety (**1 h**) or the carboxylic acid moiety (**1 i**) showed that both functionalities can be omitted and prenylation still occurs (Table 2). It is worth to note that omitting the carboxylic acid moiety led in general to higher conversion than using C5‐substituted tryptophan derivatives. Although the docking poses of substrates **1 h** and **1 i** align closely with the one from L‐**1 a** in the active site of DMATS from *A. fumigatus* (Figure 5, PDB code: 3I4X^[27]^), we hypothesized that the binding of these two indole derivatives in the active site is considerably weaker due to missing polar groups and therefore reduced interactions. This assumption was supported by the measured *K*_M_ values: In both cases, the *K*_M_ increased (*K*_M_= 3.4 and 4.9 mM, respectively, for **1 h** and **1 i**) and additionally maximum rates were reduced to 5.3 and 8.8 U/g_CFE_ for **1 h** and **1 i** (Figure S22 and S23).

As here always DMAPP was used as alkyl substrate, it remains to be clarified whether DMATS homologs would lead to changed regioselectivity as recently reported for FgaPT2.^[18e]^

### Evaluation of variants for improved conversion of C5‐substitued tryptophans

**Figure 6:**
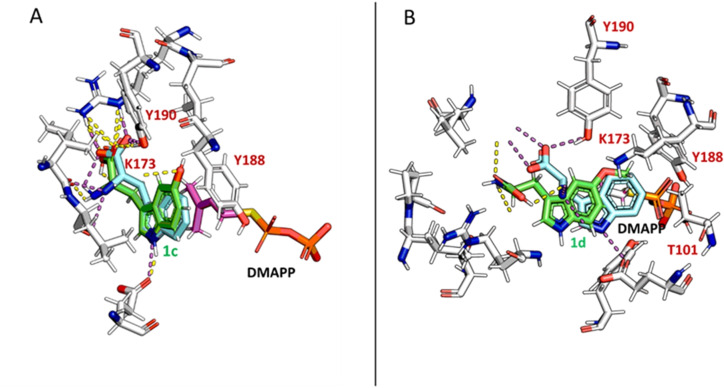
Structural alignment of docking poses of L**‐1 a** (turquoise) with (A) L**‐1 c** and (B) L**‐1 d** (both marked in green) in the active site of DMATS from *A. fumigatus*. Polar interactions of L**‐1 a** are highlighted in magenta and polar interactions of tryptophan derivatives in yellow. Numbering in red is according to *A. fumigatus* and corresponds to Y197 for Y190, Y195 for Y188, K180 for K173 and T108 for T101 in *A. japonicus*.

For a rational protein engineering approach, the C5‐substituted tryptophans L‐**1 c** and L‐**1 d** were docked into the active site of DMATS from *A. fumigatus* together with DMAPP (Figure 6). The docking pose of L**‐1 c** aligned well with the published crystal structure harboring L**‐1 a** and the unreactive sulfur‐analog of DMAPP, DMSPP (PDB code: 3I4X).^[27]^ The indole ring of L‐**1 c** is slightly tilted out of position (the distance from the C4 to the C1 of DMAPP changes from 3.8 to 4.3 Å), apparently as a result of an interaction with the side chain of tyrosine Y188 (numbering according to *A. fumigatus*). The methoxy derivative L‐**1 d**, on the other hand, is displaced by 3.3 Å relative to L‐**1 a**, which seems to disrupt important polar interactions of **1 d** with active‐site residues, including the catalytically important Glu85 and Lys174. This displacement appears to result from steric interactions of the substrate's methoxy group with the side chains of Tyr188, Lys173, and Thr101. These residues were therefore targeted by site‐directed mutagenesis.

**Figure 7:**
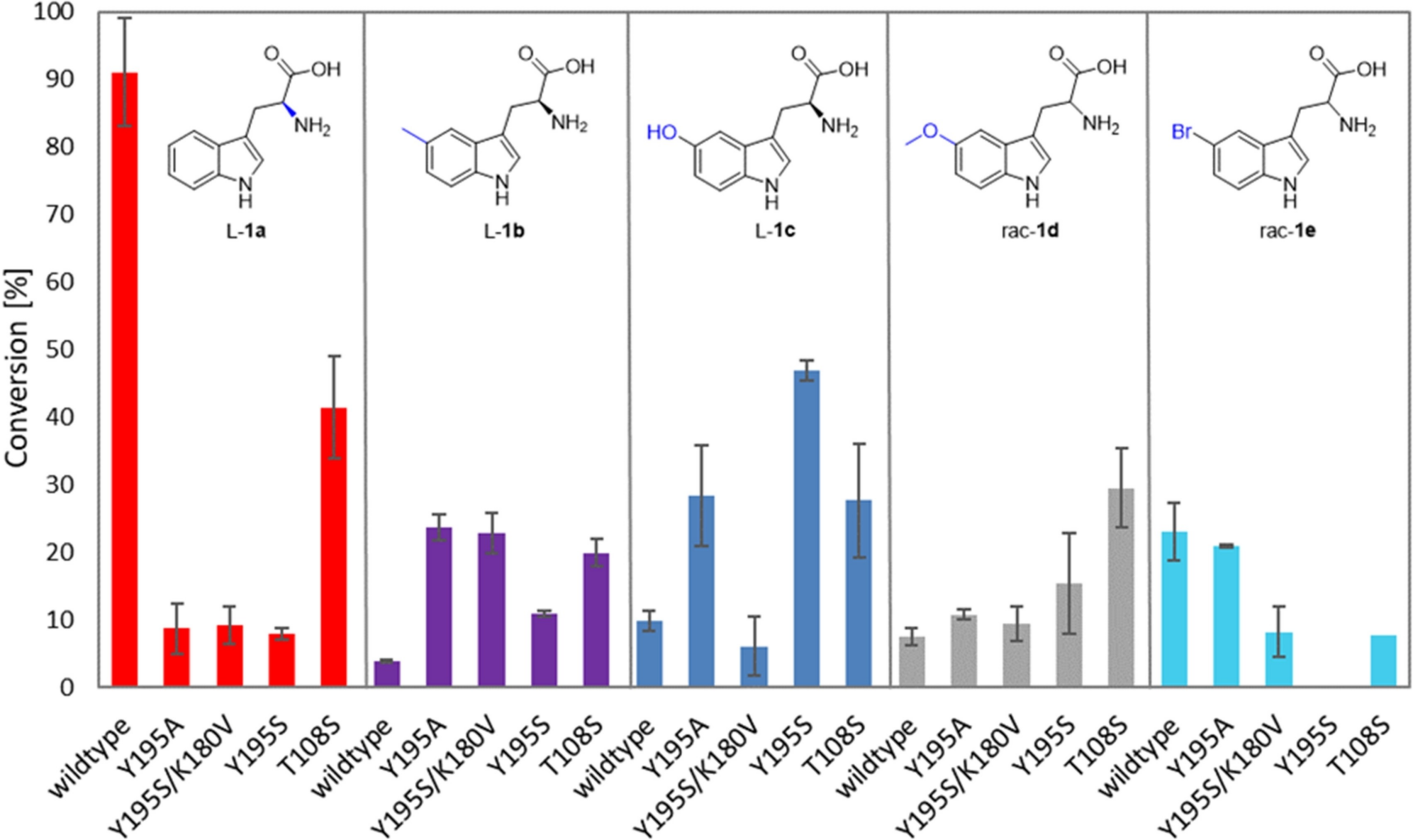
Conversion of L‐**1 a** and C5‐substituted tryptophan derivatives L‐**1 b**, L‐**1 c**, *rac*‐**1 d**, and *rac*‐**1 e** by DmaW wild‐type and DmaW variants after 24 h. Reaction conditions: tryptophan derivative (5 mM), DMAPP (5 mM), DmaW wild‐type and variant preparation [CFE of *E. coli* BL21(DE3)] (20 mg_CFE_/mL), Tris/HCl buffer (50 mM, pH 7.5, 5 mM CaCl_2_), DMSO (5 % v/v), 30 °C, 24 h.

To reduce steric hindrance, the size of the amino acid side chains was reduced by creating the variants Y195A, Y195S, Y195S/K180V and T108S which expressed about as well as the wild‐type (Figure S10). The variants for the Y195 position displayed improved conversion compared to the wild‐type for 5‐substituted tryptophan derivatives **1 b**–**1 d** (Figure 7). Exchange to serine gave improved conversion with L**‐1 c** and *rac* **‐1 d** (46.9±1.5 % and 15.4±7.4 % conv. within 24 h, compared to 9.8±1.4 % and 7.5±1.3 % with the wild‐type). An even higher increase in conversion was obtained for the 5‐methoxy‐substituted tryptophan *rac*‐**1 d** with T108 S (29.5±5.8 % conv. within 24 h). Moreover, for L**‐1 b**, an increase in conversion was observed with all investigated variants. Compared to the wild‐type, which converted only 3.9 % of L**‐1 b**, single variant Y195 A and double variant Y195S/K180 V showed increased conversions of 23.7±1.9 % and 22.9±2.9 %, respectively. No improvement was obtained for *rac* **‐1 e** with any variant; actually, conversions dropped in all cases, which was unexpected.

### Semi‐preparative prenylation of tryptophan derivatives

Having now a small library of DMATSs in hand, the possibility to perform semi‐preparative biotransformations was evaluated. For these experiments the substrate concentration was raised from 5 mM to 10 mM in these reactions, which was expected to result in higher space‐time yield at the cost of reduced conversion. Reactions on 100–200 mg scale (tryptophan derivative) afforded the prenylated tryptophans with up to 90 % conversion and up to 80 % isolated yield (Table 3). The products L‐**2 a**, L‐**2 b**, L‐**2 e**, **2 h**, **2 i** were isolated by preparative HPLC and silica gel chromatography (Supporting Information).

**Table 3:** Semi‐preparative prenylations of L‐**1 a** and derivatives by DmaW wild‐type and variants.

Substrate	Conv.^[a]^	Yield		Product(s)	Enzyme
	[%]	[%]	[mg]		
L‐**1 a**	90*	80	109	L‐**2 a**	*A. japonicus* WT *A. japonicus* Y195S *A. japonicus* T108S *A. japonicus* WT *A. japonicus* WT *C. purpurea* WT
L‐**1 c**	80*	50	72	L‐**2 c**	
*rac*‐**1 d**	15**	10	20^[c]^	**2 d** ^[b]^	
L‐**1 f**	90*	10/20/20	20/40/40	L‐**2 f**/**2 a**/**4 f**	
**1 h**	20**	8	6	**2 h**	
**1 i**	40**	10	6	**2 i**	

[a] Conversion (consumption of substrate 1) determined by reversed‐phase HPLC‐UV (262 nm) on an achiral stationary phase, using propiophenone (2 mM) as internal standard. Reaction conditions: tryptophan derivative 1 (10 mM), DMAPP (10 mM), DMATS preparation [CFE of *E. coli* BL21(DE3), 5 mg_CFE_/mL], Tris/HCl buffer (50 mM pH 7.5, 5 mM CaCl_2_), DMSO (5 % v/v), 30 °C, *24 h/**72 h, reaction volume: 50 mL. [b] Enantiomeric excess of product not determined due to low conversion. [c] Isolated as Boc‐protected derivative

## Conclusion

Regioselective C−C bond formation at aromatic non‐pre‐functionalized positions is a challenge for established chemistry. Biocatalysts may offer alternatives due to the possibility to position the two reaction partners in a very precise position relative to each other in the active site of the biocatalyst.^[2c]^ C4‐Dimethylallytryptophan synthases (4‐DMATSs) were here investigated for their potential in organic synthesis. Starting from a less characterized DMATS from *A. japonicus*, further 4‐DMATSs were identified and tested. All three investigated 4‐DMATSs showed activity with tryptophan and several of its derivatives. It turned out that for higher substrate concentrations the low solubility of the tryptophan derivative in buffer represents a challenge as well as an intrinsic substrate inhibition by DMAPP. Three wild‐type enzymes and a focused library of variants were investigated for the C4‐prenylation of L‐tryptophan **1 a** and derivatives bearing substituents either at the demanding position C5 or at the nitrogen atoms. All non‐natural substrates were successfully prenylated with conversions up to 90 %. Additionally, it was shown that also truncated tryptophan derivatives like tryptamine (**1 i**) are substrates. Investigating selected variants led to the identification of variant Y195S, which allowed a five‐fold increase of conversion for 5‐hydroxy substituted tryptophan derivative L‐**1 c**, and variant T108S, which improved the conversion of *rac*‐**1 d** almost 3‐fold.

The feasibility study on semi‐preparative scale (100 mg, 10 mM substrate concentration) resulted in isolated yields of up to 90 %. It demonstrated for the first time the potential of C4‐prenylation of unprotected tryptophan derivatives for synthetic purposes at a reasonable substrate concentration. Such a single‐step prenylation allows to shorten previously reported chemical synthesis routes,^[33]^ *e. g*., of C4‐prenylated tryptamine (**2 i**), to a single step and does not require preactivation or protecting groups. This research opens the door for regioselective C−C bond formation at C4 of tryptophan derivatives.

## Experimental Section

**Chemicals**: All chemicals and media ingredients were purchased from Sigma‐Aldrich (Vienna, Austria), Roth (Karlsruhe, Germany), Merck (Darmstadt, Germany), Fluka (Buchs, Switzerland) or Becton, Dickinson and Company (Franklin Lakes, NJ, USA).

**Gene synthesis, cloning, expression, and protein purification**: General information on strains and plasmids, and the details of gene design and cloning protocols can be found in the Supporting Information, “Section S1. Supporting Methods”. Implemented variants were generated by QuikChange II XL Site‐Directed Mutagenesis Kit (Agilent, Santa Clara, USA), using primers as listed in the Supporting Information, “List of primers for construction of DmaW variants”. The presence of the desired mutations in all constructs was verified by sequencing. Production of wild‐type DMATS was achieved in *E. coli* BL21(DE3), and DmaW variants were produced in *E. coli* ArcticExpress(DE3). Cultivation of *E. coli* ArcticExpress(DE3) cells was conducted in 600 mL of lysogeny broth (LB) medium (10 g/L tryptone, 5 g/L yeast extract, 5 g/L NaCl) containing kanamycin (50 μg/mL) and gentamycin (20 μg/mL) in the precultures and in the main cultures without antibiotics, as suggested by the cell manufacturer's protocol.^[34]^ Cultivation of *E. coli* BL21(DE3) was conducted in 600 mL of terrific broth (TB) medium (24 g/L yeast extract, 12 g/L tryptone, 4 mL/L glycerol, potassium phosphate buffer (100 mM, pH 7.5) containing kanamycin (50 μg/mL). Cultures were initially incubated at 30 °C and 37 °C for ArcticExpress(DE3) and BL21(DE3), respectively, with shaking at 120 rpm. At an optical density (OD_600_ nm) of 0.5–1.0, isopropyl β‐D‐1‐thiogalactopyranoside (IPTG) was added to a final concentration of 0.5 mM to induce the expression of DMATS. Incubation was continued at 13 °C for ArcticExpress(DE3) and 20 °C for BL21(DE3) with shaking at 120 rpm for 20 h. Cells were then harvested by centrifugation and resuspended in sodium phosphate buffer (50 mM, 150 mM CaCl_2_, pH 7.5). The cells were disrupted using a Soniprep150 ultrasonicator (MSE, London, U.K.), employing 5 min bursts with an amplitude of 30 % with 2 min intervals at 4 °C. Cell debris was removed by centrifugation at 15,000 rpm (42,300 × g) for 15 min and either loaded onto a 5 mL His‐Trap HP column (GE Healthcare) or lyophilized on a freeze‐dryer (Christ alpha 1–4basic). For His‐tag purification, the target protein was eluted using 250 mM imidazole in sodium phosphate buffer (50 mM, pH 7.5) containing 150 mM NaCl. The purified enzyme was desalted using PD10 columns and sodium phosphate buffer (50 mM, pH 7.5) containing 150 mM NaCl without imidazole.

**Biotransformations**: Biotransformations (1 mL) with DMATS preparation [CFE of *E. coli* BL21(DE3)] or purified DmaW from *A. japonicus* contained the tryptophan derivative (0.5–25 mM), DMAPP (0.5–25 mM), DMSO (0–25 % v/v) and CFE (10–40 mg_CFE_/mL corresponding to 6.5–26 mM pure DmaW) or purified DmaW (100–400 μg/mL) in Tris/HCl buffer (50 mM pH 7.5, 5 mM CaCl_2_). Reactions were performed on the lab bench or under oxygen exclusion in the glove box (for abrine (**1 f**) oxidation tests) using a thermoshaker. For determination of conversion, samples (150 μL) were taken from the reaction mixtures after 0, 60 min and 24 h, and for kinetics after 0, 5, 10, 20, 40 and 60 min after incubation at 30 °C, and were quenched with acetonitrile (150 μL, containing 2 mM of propiophenone as internal standard) and after vigorous shaking (10 sec) analyzed on HPLC‐UV and HPLC‐MS. Substrate stocks (50 mM) were prepared in Tris/HCl buffer (50 mM, pH 7.5, 5 mM CaCl_2_). All stocks in buffer were sonicated (30 sec) to obtain a clear solution. Details of columns and analytical methods, with chromatograms, can be found in the Supporting Information, “Analysis: Chromatography columns, conditions, and retention times for investigated substrates”.

**Semi‐preparative biotransformations**: Reactions with tryptophan derivatives (0.5 mmol) as prenyl acceptors were set up on 50 mL scale, using DmaW wild‐type and variants preparation [CFE of *E. coli* BL21(DE3)] (250–1000 mg) and DMAPP (123 mg, 0.5 mmol). The reactions were incubated at 30 °C, 120 rpm for 24–48 h. Reactions were quenched with acetonitrile (50 mL) and, after intensive vortexing, centrifuged for 20 min (14,000 rpm, 21,000× g). The remaining clear solutions were subjected to preparative HPLC for isolation of the desired prenylated compounds. Reactions containing indole‐3‐propionic acid (**1 h**) and tryptamine (**1 i**) were acidified with concentrated aqueous HCl solution (100 μL) or basified with saturated aqueous Na_2_CO_3_ solution (50 mL), respectively, vortexed, and extracted with ethyl acetate (50 mL). After centrifugation for 10 min, the organic layer was collected and evaporated under reduced pressure. The remaining solution was subjected to preparative HPLC as described above. Fractions containing the desired prenylated tryptophan were evaporated under reduced pressure, followed by NMR analysis.

Following the general procedure of semi‐preparative prenylation of tryptophan derivatives, L‐**1 a** (103 mg, 0.5 mmol) afforded L‐**2 a** (109 mg, 0.4 mmol, 80 %) as a white powder after preparative HPLC. ^**1**^ **H NMR** (300 MHz, DMSO‐d6): *δ*=11.06 (d, *J*=2.5 Hz, 1H, N1−H), 7.26 (d, *J*=2.3 Hz, 1H, C2−H), 7.20 (t, *J*=8.0 Hz, 1H, C5−H), 6.96 (t, *J*=7.6 Hz, 1H, C6−H), 6.72 (d, *J*=7.1 Hz, 1H, C7−H), 5.33 (t, *J*=7.1 Hz, 1H, prenyl−CH), 3.74–3.60 (m, 2H, prenyl−CH_2_), 3.59 (dd, *J*=3.3 Hz, 15.4 Hz, 2H, beta‐CH_2_), 3.48 (dd, *J*=3.3, 10.5 Hz, 2H, alpha‐CH) 3.02 (dd, *J*=10.4, 15.4 Hz, 2H, beta‐CH_2_), 1.72 (s, 6H, prenyl−CH_3_); ^**13**^ **C NMR** (75 MHz, DMSO‐d6): *δ*=170.8, 137.6, 133.9, 131.7, 125.3, 124.8, 124.3, 121.4, 118.9, 110.8, 109.9, 55.9, 31.8, 29.7, 26.1, 18.3. **MS** (ESI, pos.): *m*/*z*=273 [M+H^+^].

Following the general procedure of semi‐preparative prenylation of tryptophan derivatives, L‐**1 c** (110 mg, 0.5 mmol) afforded L‐**2 c** (72 mg, 0.25 mmol, 50 %) as a white powder after preparative HPLC. ^**1**^ **H NMR** (300 MHz, DMSO‐d6): *δ*=10.62 (d, *J*=2.5 Hz, 1H, N1−H), 7.12 (d, *J*=2.6 Hz, 1H, C2−H), 6.66 (d, *J*=8.5 Hz, 1H, C6−H), 6.98 (d, *J*=8.5 Hz, 1H, C7−H), 5.09 (t, *J*=6.4 Hz, 1H, prenyl−CH), 3.67 (dd, *J*=14.9, 6.9 Hz, 2H, prenyl−CH_2_), 3.52 (dd, *J*=3.3 Hz, 15.4 Hz, 2H, beta‐CH_2_), 3.40 (dd, *J*=3.2, 10.6 Hz, 2H, alpha‐CH), 2.90 (dd, *J*=10.6, 15.6 Hz, 2H, beta‐CH_2_), 1.72 (s, 3H, prenyl−CH_3_), 1.61 (s, 3H, prenyl−CH_3_) ^**13**^ **C NMR** (75 MHz, DMSO‐d6): *δ*=170.6, 147.8, 132.3, 129.7, 126.2, 125.7, 124.9, 117.8, 112.0, 110.3, 109.5, 55.7, 29.6 26.1, 25.2, 18.6. **MS** (ESI, pos.): *m*/*z*=289 [M+H^+^].

Following the general procedure of semi‐preparative prenylation of tryptophan derivatives, Boc‐protection as described in section “Boc‐protection for semi‐preparative biotransformations” and acidic extraction with EtOAc, L‐**1 d** (117 mg, 0.5 mmol) afforded Boc‐protected L‐**2 d** (20 mg, 0.05 mmol, 10 %) as a white‐yellowish powder after silica chromatography (CH_2_Cl_2_:MeOH 9 : 1+0.1 % Acetic acid). ^**1**^ **H NMR** (300 MHz, Methanol‐d4): *δ*=7.21 (d, *J*=8.8 Hz, 1H, C2−H), 7.13–6.96 (m, 1H, C6−H), 6.74 (d, *J*=6.3 Hz, 1H, C7−H), 5.60 (t, *J*=3.8 Hz, 1H, prenyl−CH), 4.39 (dd, *J*=7.7, 4.9 Hz, 1H), 3.83 (s, 3H, methoxy−CH_3_), 3.34–3.13 (m, 2H, beta‐ and alpha‐CH), 3.09 (dd, *J*=14.7, 7.6 Hz, 1H, beta‐CH_2_), 1.43 (d, *J*=2.4 Hz, 6H, prenyl−CH_3_), 1.37 (s, 9H). ^**13**^ **C NMR** (75 MHz, Methanol‐d4): *δ*=179.1, 157.6, 135.5, 131.7, 127.8, 127.8, 115.4, 115.2, 113.5, 104.3, 104.0, 83.1, 58.9, 58.7, 52.4, 52.4, 52.1, 51.8, 51.6, 51.3, 51.0, 50.7, 31.5, 31.2, 19.0. **MS** (ESI, pos.): *m*/*z*=403 [M+H^+^].

Following the general procedure of semi‐preparative prenylation of tryptophan derivatives, L‐**1 f** (109 mg, 0.5 mmol) afforded L‐**2 f** (21 mg, 0.075 mmol, 15 %) as a white powder after preparative HPLC. ^**1**^ **H NMR** (300 MHz, DMSO‐d6): *δ*=10.76 (d, *J*=18.4, 11.1 Hz, 1H, N1−H), 7.03–7.01 (m, 2H, C2−H), 6.99 (d, *J*=1.1 Hz, 1H, C5−H), 6.79 (t, *J*=8.1 Hz, 1H, C6−H), 6.54 (d, *J*=8.1, 7.2 Hz, 2H, C7−H), 5.15 (t, *J*=7.0 Hz, 1H, prenyl−CH), 3.55 (dd, *J*=15.7, 2.9 Hz, 2H, beta‐CH_2_), 3.40 (dd, *J*=10.4, 3.1 Hz, 2H, alpha‐CH), 2.80 (dd, *J*=15.4, 10.7 Hz, 1H, beta‐CH_2_), 2.41 (N−CH_3_, under DMSO peak) 1.55 (s, 6H, prenyl−CH_3_) ^**13**^ **C NMR** (75 MHz, DMSO‐d6): *δ*=170.2, 137.7, 133.9, 131.7, 125.2, 124.6, 124.3, 121.5, 118.9, 110.9, 109.9, 55.9, 31.8, 29.7, 26.1, 18.3. **MS** (ESI, pos.): *m*/*z*=287 [M+H^+^].

Following the general procedure of semi‐preparative prenylation of tryptophan derivatives and acidic extraction with EtOAc, **1 h** (95 mg, 0.5 mmol) afforded **2 h** (6 mg, 0.023 mmol, 5 %) as a white powder after preparative HPLC. ^**1**^ **H NMR** (300 MHz, CD_3_OD) *δ*=7.25–7.11 (m„ 1H, C5−H), 7.02 (s, 1H, C2−H), 6.98 (t, *J*=7.7 Hz, 1H, C6−H), 6.75 (d, *J*=7.2 Hz, 1H, C7−H), 5.35–5.31 (m, 1H, prenyl−CH), 3.74 (d, 2H, *J*=6.8 Hz, prenyl−CH_2_), 3.22 (m, *J*=7.9 Hz, 2H, beta‐CH_2_), 2.66 (dd, *J*=8.8, 6.8 Hz, 2H, alpha‐CH_2_), 1.78 (s, 3H, prenyl−CH_3_), 1.76 (s, 3H, prenyl−CH_3_). **MS** (ESI, pos.): *m*/*z*=258 [M+H^+^].

Following the general procedure of semi‐preparative prenylation of tryptophan derivatives and basic extraction with EtOAc, **1 i** (80 mg, 0.5 mmol) afforded **2 i** (6 mg, 0.026 mmol, 5 %) as a white powder after preparative HPLC. ^**1**^ **H NMR** (300 MHz, CDCl_3_): *δ*=7.35–7.20 (m, 5H, C5−H), 7.21–7.05 (m, 2H, C2−H, C6−H), 6.92 (d, *J*=7.0 Hz, 1H, C7−H), 5.29 (t, *J*=6.2 Hz, 1H, prenyl−CH), 3.64 (d, *J*=5.8 Hz, 2H, prenyl−CH_2_), 3.49–3.31 (m, 1H, beta‐CH_2_), 3.17 (t, *J*=6.0 Hz, 1H, alpha‐CH_2_), 1.77–1.67 (m, 6H, prenyl−CH_3_), 1.25 (s, 1H, alpha‐NH_2_) **MS** m/z 229 [M+H^+^].

**Boc‐protection for semi‐preparative biotransformations**: For semi‐preparative biotransformation with 5‐methoxy‐DL‐tryptophan (*rac*‐**1 d**), the mixture was carefully acidified to pH 3.0 applying a conc. HCl solution after 24 h of reaction. The aqueous phase was extracted with ethyl acetate (50 mL) and the organic phase was discarded. The aqueous phase was neutralized by addition of 10 M NaOH solution. An equal volume of *tert*‐butanol, another 2 equivalents of 10 M NaOH and di‐*tert*‐butyl dicarbonate (Boc‐anhydride, 2.0 eq) was added. Next, the reaction mixture was stirred at room temperature for 24 hours, after which it was diluted with water (100 mL) and neutralized by addition of conc. HCl solution. The resulting solution was extracted with ethyl acetate (100 mL) and concentrated in vacuum. As a final step, the crude extract was purified by column chromatography (silica gel 60; CH_2_Cl_2_/MeOH/AcOH=90/10/0.1; product *R*_f_=0.3).

**Docking experiments**: For the docking in YASARA, the published crystal structure of DMATS FgaPT2 from *Aspergillus fumigatus* in complex with DMSPP (the sulfur analogue of DMAPP) and L‐tryptophan was chosen as a template (PDB code: 3I4X). Preparation of the protein structure was performed in PyMOL by removal of water molecules and the bound tryptophan ligand. The prenyl donor analogue DMSPP was kept. This was followed by cleaning the structure in YASARA by addition of missing hydrogens to the protein. Within the active site next to DMSPP, a suitable center point was selected for a 10 Å docking box. Additionally, the force field (AMBER03) was chosen, and partial charges were calculated. For the preparation of the respective ligand to dock, the different tryptophan derivatives were drawn in ChemDraw and the conformational energy of the structure was minimized in Chem3D using the force field MM2. After export from Chem3D as PDB files and import in YASARA, a force field (AMBER03) was selected and partial charges were calculated. The docking was performed in YASARA using the “dock_run” macro, which provides an interface to the Autodock Vina program. Different docking modes were analyzed and saved as a PDB file. The best docking poses were selected based on the highest binding energies. Further analysis of the docking poses was performed in PyMOL, using measurements of angles and distances to find potential amino acids for exchange.

PDB files of the docking poses of tryptophan derivatives in the active site of DMATS can be found as Supporting Information.

## Conflict of interest

The authors declare no conflict of interest.

1

## Supporting information

As a service to our authors and readers, this journal provides supporting information supplied by the authors. Such materials are peer reviewed and may be re‐organized for online delivery, but are not copy‐edited or typeset. Technical support issues arising from supporting information (other than missing files) should be addressed to the authors.

Supporting Information

Supporting Information

## Data Availability

The data that support the findings of this study are available in the supplementary material of this article.
