# Supplementary material for: Regioselective Biocatalytic C4‐Prenylation of Unprotected Tryptophan Derivatives
Source: Chembiochem. 2022 Jul 13;23(17):e202200311. doi: 10.1002/cbic.202200311 (PMC9540666; doi:10.1002/cbic.202200311)
Supplement: Supplementary file 2 — Supporting Information [file CBIC-23-0-s002.pdf]

ChemBioChem

Supporting Information

Regioselective Biocatalytic C4-Prenylation of Unprotected Tryptophan Derivatives

Bettina Eggbauer, Joerg H. Schrittwieser, Bianca Kerschbaumer, Peter Macheroux, and Wolfgang Kroutil*

Table of contents

Section S1. Supporting Methods ... 3
Cultivation and Purification of DMATS ... 3
Strains and plasmids ... 3
Cloning and expression of DMATS and variants ... 3
Creation of variants ... 9
Chromatographic purification of DmaW ... 10
SDS-PAGE ... 12
Synthesis of DMAPP. ... 12
Characterization of Side Reaction. ... 13
Determination of enantiomeric excess of D-1f and D-1g ... 14
Section S2. Supporting Data ... 15
BLAST search ... 15
Estimation of enzyme content in the DmaW preparation [CFE of *E. coli* BL21(DE3)] ... 19
Effect of substrate concentration and DMSO addition on the prenylation of tryptophan derivatives by DmaW from *A. japonicus* ... 20
Test for product inhibition ... 21
Blank studies with DmaW preparation [CFE of *E. coli* BL21(DE3)] ... 22
Tryptophan content in *E. coli* BL21(DE3) CFE ... 25
Sequence alignment of DMATSs from *A. japonicus*, *C. purpurea* and *T. benhamiae* ... 26
Engineering of DmaW from *A. japonicus* / Expression of DmaW variants ... 27
Products obtained in the prenylation of L-abrine (1f) by DmaW ... 27
Investigation of purified DmaW in prenylation of L-1f ... 31
Investigation of different CFE preparations in prenylation of L-1f ... 32
HR-MS results of products in prenylation of L-1f ... 34
OrbiTrap measurement of L-1f prenylation ... 35
Activity measurements for 1h and 1i using DmaW from *A. japonicus* ... 39
Analysis: Chromatography columns, conditions, and retention times ... 40
Preparative HPLC chromatograms ... 44
NMR spectra of synthesized compounds ... 47
OrbiTrap chromatograms and spectra ... 62
Reference chromatograms of HPLC-UV ... 74
Optical purity of 1f and 1g ... 77
Structural Alignment and Docking Results ... 79
References ... 81

Section S1. Supporting Methods

Solvents used were of HPLC grade. Column chromatography was performed on silica gel 60 0.040–0.063 mm (Merck; Darmstadt, Germany; 230–400 mesh). ^{1}H and ^{13}C NMR spectra were recorded on a Bruker Avance III instrument (300 MHz for ^{1}H and 75 MHz for ^{13}C) in DMSO-d_6 or CD_3OD. High-resolution mass spectrometry (HRMS) was performed using an Agilent 6230 TOF LC/MS connected to an Agilent 1260 Infinity Series HPLC.

Cultivation and Purification of DMATS

Strains and plasmids

Heat-competent *E. coli* BL21(DE3) cells [F–ompT hsdSB(rB-mB-) gal dcmrne131 (DE3)] were purchased from New England Biolabs (Ipswich, MA, USA). Heat-competent *E. coli* Arctic Express (DE3) cells were purchased from Agilent Technologies (USA). pET-22b(+) vectors containing DMATS genes from *T. benhamiae* and *C. purpurea* (sequence codon-optimized for expression in *E. coli*) were purchased from BioCat (Heidelberg, Germany) and DmaW from *A. japonicus* was obtained as optimized sequence in pETM-11[1] vector from Graz University of Technology. The most important properties of these plasmids and references to relevant publications are provided in Table S1.

Cloning and expression of DMATS and variants

Codon-Optimized DMATS sequences

DmaW from Aspergillus japonicus

DNA sequence:

tctAGAATAATTTTGTTTAACTTTAAGAAGGAGATATACCATGAAACATCACCATCACCATCAC
CCCATGGGCACTGCTGGTCAAGGTATTAAGACTGGTAATGCTTCTGATTGCGAAGTCTACAGAA
CTTTGTCTGTTGCTTTGGATTTCGCCAATCAAGATGAAGAATTGTGGTGGCATTCTACTGCTCC
AATGTTTGCTCAAATGTTGCAATCTACCAACTACAACTTACACGCTCAGTACAAGCACTTGTTG
ATCTACAAGAAGAACGTCATTCCATTCTTGGGTGTTTACCCAACTAATGATAAGCCAAGATGGT
TGTCCATTTTGACCAGATATGGTACTCCATTCGAATTGTCTTTGAACTGTTCTGGTCCATTGGT
TAGATACACTTACGAACCTATTAACGCTGCTACTGGTACTGCTAGAGATCCATTCAATACTCAT

GCTGTTTGGGACTCATTGGAACAATTGATGGCTTTACAATCCGGTATCGATTTGGATTTGTTCA
GACACTTCAAGAACGACTTGACTTTGTCTGCTGAAGAATCCGAATACTTGTACAAGAACAACTT
GGTCGGTGAACAAATCAGAACCCAAAACAAATTGGCTTTGGACTTGCAAGATGGTGAATTCGTT
GTTAAGACTTACATCTACCCAGCCTTGAAATCTTTGGCTACTGGTAGAAGTATCCACGAATTGG
TTTTTGGTTCTGCTTTCAGATGGTCTAAGCAATACCCAGAATTGAGAAAGCCATTGGATACCTT
GGAACAATACGTTTATTCTAGAGGTCCATCTTCTACTGCCTCTCCAAGATTATTGTCTTGCGAT
TTGATTGACCCTACCAAGTCCAGAATCAAAATCTACTTGTTGGAAAGAATGGTCACTTTGGAAG
CCTTGGAAGATTTGTGGACTATGGGTGGTGAAAGAACTGATGCTTCTACTTTGGCTGGTTTGGA
AATGATTAGAGAATTGTGGGAATTGATCAGATTGCCAGCTGGTTTACAATCTTATCCAGCTCCA
TATTTGCCAATCGGTACTATTCCAGATGAACAATTGCCATTGATGGCCAACTACACCATCCATC
ATGATGATCCAGTTCCAGAACCACAAGTTTACTTTACTACTTTCGGTAGAAACGACATGCAAAT
TGCTGATGCTTTGGCTACTTTCTTCGAACGTCGTGGTTGGCACGAAATGGCTAGACAGTACAAA
GCTGAATTGTGCTCTCATTACCCACATGCTGATCACGAAACTTTGAACTACTTGCACGCTTACA
TCTCATTCTCTTACAGAAAGAACAAGCCATACTTGTCCGTCTACTTGCAATCTTTGGAAACTGG
TGATTGGGTTACCTCATCCTTCAACTCTGTCCATGTGGACCCTGGCCTTTCAGCCACCGTCCAA
GAGCTGTCCAAGCTGACCAAGACCGCCGGAACCACCGTTAGGGAGACAAAACTACCACTAACCC
CAGATGGATCAGAGCCTGGCGTCATCACCCAGTACTAGGCGGCCGCACTCgag

Protein sequence:

MGTAGQGIKTGNASDCEVYRTLSVALDFANQDEELWWHSTAPMFAQMLQSTNYNLHAQYKHLLI
YKKNVIPFLGVYPTNDKPRWLSILTRYGTPFELSLNCSGPLVRYTYEPINAATGTARDPFNTHA
VWDSLEQLMALQSGIDLDLFRHFKNDLTLSAEESEYLYKNNLVGEQIRTQNKLALDLQDGEFVV
KTYIYPALKSLATGRSIHELVFGSAFRWSKQYPELRKPLDTLEQYVYSRGPSSTASPRLLSCDL
IDPTKSRIKIYLLERMVTLEALEDLWTMGGERTDASTLAGLEMIRELWELIRLPAGLQSYPAPY
LPIGTIPDEQLPLMANYTIHHDDPVPEPQVYFTTFGRNDMQIADALATFFERRGWHEMARQYKA
ELCSHYPHADHETLNYLHAYISFSYRKNKPYLSVYLQSLETGDWVTSSFNSVHVDPGLSATVQE
LSKLTKTAGTTVRETKLPLTPDGSEPGVITQY

DMATS from Trichophyton benhamiae

DNA sequence:

tctAGAAATAATTTTGTTTAACTTTAAGAAGGAGATATACCATGGGCAGCAGCCATCATCATCA
TCATCACAGCAGCGGCCTGGTGCCGCGCGGCAGCCATATGGGTAGCATTGAAATTCCGAATTGT

AGTGGTAGTATTGTGTATAAAACCATTAGTGACTTCATCGACTTCCCGAATCATGAACAGAAAC
TGTGGTGGCATAGCACCGCACCGATGTTCGCAGAAATGCTGCGTGTTGCCGGCTATGATCTGCA
TAGTCAGTATAAAATTCTGGGTATCTTCCTGAATCATGTTATTCCGTTCCTGGGTGTGTATCCG
ACCCGCATTAATAATCGTTGGCTGAGCATTCTGACCCGTTATGGTACCCCGTTCGAACTGAGTC
TGAATTGCAGTCAGAGCCTGGTTCGCTATACCTATGAACCGATTAATAGCGCCACCGGTACCGT
TAAAGATCCGTTCAATACCCATAGTATCTGGGATGCACTGGATCGCCTGATGCCGCTGCAGAAA
GGTATTGATCTGGAATTCTTCAAACATCTGAAACAGGATCTGACCGTGGATGATCAGGATAGCG
CCTATCTGCTGGAAAATAATCTGGTTGGCGGTCAGATTCGTACCCAGAATAAACTGGCACTGGA
TCTGAAAGGCGGCAACTTCGTTCTGAAAACCTATATCTATCCGGCCCTGAAAGCACTGGCAACC
GGCAAAAGTATTAAAACCTTAATGTTCGATAGCGTGTATCGTCTGTGTCGCCAGAATCCGAGTC
TGGAAGCCCCGCTGCGTGCCCTGGAAGAATATGTTGATAGCAAAGGCCCGAATAGTACCGCAAG
CCCGCGCCTGCTGAGCTGTGATCTGATTGATCCGAGCAAAAGTCGCGTGAAAATCTATATTCTG
GAACTGAATGTTACCCTGGAAGCCATGGAAGATCTGTGGACCATGGGTGGTCGCCTGAATGATG
CAAGCACCCTGGCCGGTCTGGAAATGCTGCGCGAACTGTGGGATCTGATTAAACTGCCGCCGGG
TATGCGTGAATATCCGGAACCGTTCCTGCAGCTGGGTACCATTCCGGATGAACAGCTGCCGCTG
ATGGCCAATTATACCCTGCATCATGATCAGGCAATGCCGGAACCGCAGGTGTACTTCACCACCT
TCGGCCTGAATGATGGTCGTATTGCAGATGGTCTGGTGACCTTCTTCGAACGCCGCGGTTGGAA
TCACATGGCACAGACCTATAAAGATAGTCTGCGTGCATATTATCCGCATGCAGATCAGGAAACC
TTAAATTATCTGCATGCCTATATTAGCTTCAGTTATCGTAAAGGTAAGCCGTATCTGAGCGTGT
ATCTGCAGACCTTCGAAACCGGCGATTGGCCGATTAGCAACTTCGGCATTCCGGTTGTGAAACC
GCTGCGTAGTAATGTGGGTTGCCAGCATCCGATTAGCTTCAGCATTCCGATTACCAAAGCACCG
CTGCTGGGCGTTGCACTCgag

Protein Sequence:

MGSIEIPNCSGSIVYKTISDFIDFPNHEQKLWWHSTAPMFAEMLRVAGYDLHSQYKILGIFLNH
VIPFLGVYPTRINNRWLSILTRYGTPFELSLNCSQSLVRYTYEPINSATGTVKDPFNTHSIWDA
LDRLMPLQKGIDLEFFKHLKQDLTVDDQDSAYLLENNLVGGQIRTQNKLALDLKGGNFVLKTYI
YPALKALATGKSIKTLMFDSVYRLCRQNPSLEAPLRALEEYVDSKGPNSTASPRLLSCDLIDPS
KSRVKIYILELNVTLEAMEDLWTMGGRLNDASTLAGLEMLRELWDLIKLPPGMREYPEPFLQLG
TIPDEQLPLMANYTLHHDQAMPEPQVYFTTFGLNDGRIADGLVTFFERRGWNHMAQTYKDSLRA
YYPHADQETLNYLHAYISFSYRKGKPYLSVYLQTFETGDWPISNFGIPVVKPLRSNVGCQHPIS
FSIPITKAPLLGVA

DMATS from Claviceps purpurea

DNA sequence:

tctAGAAATAATTTTGTTTAACTTTAAGAAGGAGATATACCATGGGCAGCAGCCATCATCATCA
TCATCACAGCAGCGGCCTGGTGCCGCGCGGCAGCCATATGAGTACCGCAAAAGATCCGGGTAAT
GGTGTGTATGAAATTCTGAGTCTGATCTTCGACTTCCCGAGTAATGAACAGCGTCTGTGGTGGC
ATAGCACCGCACCGATGTTCGCAGCAATGCTGGATAATGCAGGTTATAATATTCATGATCAGTA
CCGTCATCTGGGTATCTTCAAAAAACATATTATCCCGTTCCTGGGTGTGTATCCGACCAAAGAT
AAAGAACGCTGGCTGAGCATTCTGACCCGCTGCGGTCTGCCGCTGGAACTGAGTCTGAATTGCA
CCGATAGCGTGGTTCGTTATACCTATGAACCGATTAATGAAGTTACCGGTACCGAAAAAGATAC
CTTCAATACCCTGGCCATTATGACCAGCGTGCAGAAACTGGCCCAGATTCAGGCAGGCATTGAT
CTGGAATGGTTCAGTTACTTCAAAGATGAACTGACCCTGGATGAAAGCGAAAGCGCCACCCTGC
AGAGTAATGAACTGGTTAAAGAACAGATTAAGACCCAGAATAAACTGGCCCTGGATCTGAAAGA
AAGTCAGTTCGCCCTGAAAGTGTACTTCTATCCGCATCTGAAAAGCATTGCCACCGGTAAAAGC
ACCCATGATCTGATCTTCGATAGTGTTCTGAAACTGAGTCAGAAACATGATAGCATTCAGCCGG
CCTTCCAGGTTCTGTGTGATTATGTGAGTCGTCGTAATCATAGTGCCGAAGTGGATCAGCATGG
TGCACTGCATGCACGTCTGCTGAGTTGCGATCTGATTGATCCGGCCAAAAGCCGCGTTAAAATC
TATCTGCTGGAAAAAACCGTGAGTCTGAGTGTTATGGAAGATCTGTGGACCCTGGGCGGTCAGC
GCGTTGATGCCAGCACCATGGATGGTCTGGATATGCTGCGTGAACTGTGGAGTCTGCTGAAAGT
TCCGACCGGCCATCTGGAATATCCGAAAGGTTATCTGGAACTGGGCGAAATTCCGAATGAACAG
CTGCCGAGCATGGCAAATTATACCCTGCATCATAATGATCCGATGCCGGAACCGCAGGTGTACT
TCACCGTGTTCGGTATGAATGATGCCGAAATTAGTAATGCACTGACCATCTTCTTCCAGCGTCA
TGGCTTCGATGATATGGCAAAAAATTATCGCGTGTTCCTGCAGGATAGTTATCCGTATCATGAC
TTCGAAAGTCTGAATTATCTGCATGCATATATTAGCTTCAGCTATCGCCGCAATAAACCGTATC
TGAGCGTGTATCTGCATACCTTCGAAACCGGTCGTTGGCCGGTGTTCGCAGATAGTCCGATTAG
CTTCGATGCATATCGTCGCTGTGAACTGAGCACCAAACTCgag

Protein Sequence:

MSTAKDPGNGVYEILSLIFDFPSNEQRLWWHSTAPMFAAMLDNAGYNIHDQYRHLGIFKKHIIP
FLGVYPTKDKERWLSILTRCGLPLELSLNCTDSVVRYTYEPINEVTGTEKDTFNTLAIMTSVQK
LAQIQAGIDLEWFSYFKDELTLDESESATLQSNELVKEQIKTQNKLALDLKESQFALKVYFYPH
LKSIATGKSTHDLIFDSVLKLSQKHDSIQPAFQVLCDYVSRRNHSAEVDQHGALHARLLSCDLI

DPAKSRVKIYLLEKTVSLSVMEDLWTLGGQRVDASTMDGLDMLRELWSLLKVPTGHLEYPKGYL
ELGEIPNEQLPSMANYTLHHNDPMPEPQVYFTVFGMNDAEISNALTIFFQRHGFDDMAKNYRVF
LQDSYPYHDFESLNYLHAYISFSYRRNKPYLSVYLHTFETGRWPVFADSPISFDAYRRCELSTK

Table S1: Plasmids used

pEG Nr.*a*	Vector	Insert	Original organism	Antibiotic	Restriction sites		Ref.
					5’	3’	
619	pETM-11	DmaW	*A. japonicus*	Kanamycin	*Xba*I	*Xho*I	Nielsen *et al.* [2]
620	pETM-11	DmaW_ K180V /Y195S	variant from *A. japonicus*	Kanamycin	*Xba*I	*Xho*I	this work
621	pETM-11	DmaW_T1 08S	variant from *A. japonicus*	Kanamycin	*Xba*I	*Xho*I	this work
622	pETM-11	DmaW_Y1 95A	variant from *A. japonicus*	Kanamycin	*Xba*I	*Xho*I	this work
623	pETM-11	DmaW_Y1 95S	variant from *A. japonicus*	Kanamycin	*Xba*I	*Xho*I	this work
624	pET-22b(+)	*Cl.pur*DM ATS	*C. purpurea*	Kanamycin	*Xba*I	*Xho*I	Tsai *et al.* [3]
625	pET-22b(+)	*T.benha*D MATS	*T. benhamiae*	Kanamycin	*Xba*I	*Xho*I	this work

a Internal plasmid identifier number.

Creation of variants

QuikChange mutagenesis

Plasmids containing mutated DmaW genes were generated using the PCR-based QuikChange XL site-directed mutagenesis kit (Agilent Technologies; Santa Clara, USA). The necessary forward and reverse primers containing the amino acid exchange were ordered at Eurofins Genomics (Munich, Germany). All components of the PCR and the exact pipetting scheme are shown in Table S2, and the temperature program is shown in

Table S3.

Table S2: Pipetting instructions for QuikChange mutagenesis PCR.

Component	Volume [µL]
Reaction buffer	5
Template (1 µg/mL) plasmid pETM-11 with DmaW	10
Forward primer (10 pmol/µL)	1.25
Reverse primer (10 pmol/µL)	1.25
dNTPs mix	1
Quik Solution	3
dH_2O	28.5
Total	50

Table S3: Temperature program for QuikChange mutagenesis PCR.

Cycle step	Temperature [°C]	Time	Cycles
Denaturation	95	1 minute	1
Denaturation	95	50 sec	
Annealing	60	50 sec	18
Extension	68	1 minute/kb plasmid length	
Final Extension	68	7 minutes	1
Store	4	∞	

After amplification of the mutated plasmid, wild-type DNA was digested with *Dpn*I (1 µL, 37 °C, 1 h). XL10 Gold cells (45 µL), after addition of XL10-Gold β-mercaptoethanol mix (2 µL), were transformed with digested plasmid solution (2 µL) according to the supplier's protocol. From the transformation plates, overnight cultures (LB medium, 50 µg/mL kanamycin) were prepared for plasmid isolation and transformation in expression strains BL21(DE3) or ArticExpress (DE3).

List of primers for construction of DmaW variants

Table S4: Primers used in QuikChange mutagenesis PCR, highlighted in red is the mutated area

Name	Nucleotide sequence
DmaW Y195A Asymm Fw	TTGTTAAGACTGCCATCTACCCAGCCTTGAAATCTTTGGCTAC
DmaW Y195A Asymm Rv	TTTCAAGGCTGGGTAGATGGCAGTCTTAACAACGAATTCAC
DmaW K180V Asymm Fw	CAGAACCCAAAACGTATTGGCTTTGGACTTGCAAGATG
DmaW K180V Asymm Rv	CATCTTGCAAGTCCAAAGCCAATACGTTTTGGGTTCTGATTTG
DmaW Y195S Asymm Fw	TTGTTAAGACTTCCATCTACCCAGCCTTGAAATCTTTGGCTAC
DmaW Y195S Asymm Rv	TTTCAAGGCTGGGTAGATGGAAGTCTTAACAACGAATTCAC
DmaW T108S Asymm Fw	TGGTCCATTGGTTAGATACAGTTACGAACCTATTAACGC
DmaW T108S Asymm Rv	TAGCAGCGTTAATAGGTTCGTAACTGTATCTAACCAATGGACC

Chromatographic purification of DmaW

His-tag purification was carried out with liquid chromatography using an ÄKTA™ FPLC system (GE Healthcare) and the UNICORN™ control software. A 5 mL HisTrap FF column (GE Healthcare; Ni^{2+} on NTA-modified cross-linked agarose) connected to the ÄKTA (equipped with a fraction collector) was equilibrated with lysis buffer (50 mL sodium phosphate buffer, 50 mM pH 8, 150 mM NaCl). The cell-free extract obtained after cell disruption of *E. coli* BL21(DE3) cells was filtered with a 0.45 µm syringe filter and loaded onto the column using sample pump with 1.5 mL/min flow. After application of the cell-free extract the column was washed with 25 mL of 50 mM sodium phosphate buffer, 50 mM pH 8, 150 mM NaCl, 10 mM imidazole. The enzyme was eluted manually using sodium phosphate buffer, 50 mM, pH 8, 150 mM NaCl, 250 mM imidazole, collecting 5 mL fractions. Protein-containing fractions were pooled, concentrated

using VivaSpin centrifugal filters with 10 kDa molecular weight cut-off, and desalted using a GE Healthcare PD-10 column and 50 mL sodium phosphate buffer, 50 mM pH 8, 150 mM NaCl as eluent. The desalted protein solution was analyzed for protein concentration using the Biorad Bradford protein assay, lyophilized overnight and stored at –20°C until further use.

A representative SDS-PAGE of DMATS purification is shown in Figure S1 and the corresponding FPLC chromatogram is given in Figure S2.

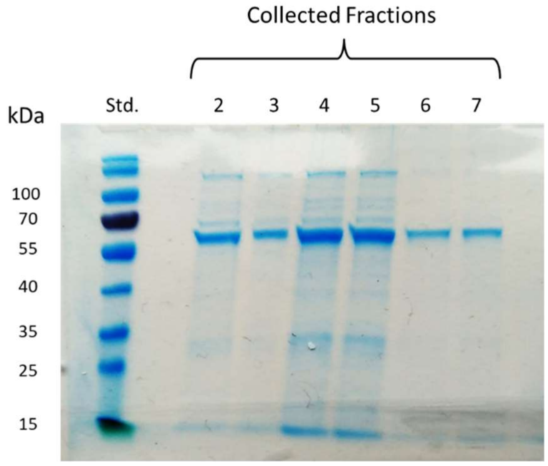

Figure S1: SDS-PAGE of HisTrap purification of DmaW wild type from *A. japonicus.* Lanes show a molecular weight standard on the left and the collected fractions 2–7 after manual elution.

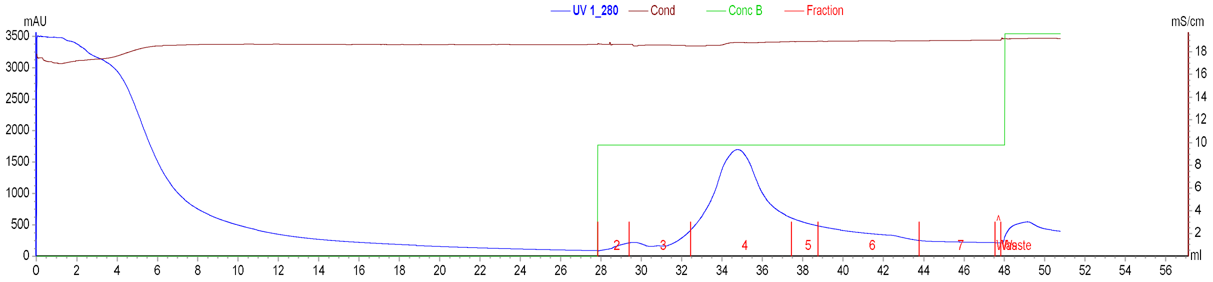

Figure S2: ÄKTA FPLC chromatogram of HisTrap purification of DmaW wild type from *A. japonicus.* Absorption at 280 nm is shown by the blue line and the collected fractions 2–7 are marked in red.

SDS-PAGE

The fractions from affinity chromatography, cell pellets and supernatants were analyzed by SDS-PAGE using commercial gels (SurePAGE^{TM}, Bis-Tris,10%; GenScript, New Jersey, USA) and buffers (Tris-MOPS-SDS Running Buffer; GenScript). Preparation of the samples containing the protein was carried out as follows: 10 µL of the eluted fraction were mixed with 10 µL Lämmli sample buffer 2× (Sigma-Aldrich) and incubated at 95 °C for 10 minutes. The mixture was loaded onto the gel. Additionally, 8 µL of the PageRuler™ Prestained Protein Ladder 10–180 kDa (Thermo Scientific) were applied as a control for the molecular weight of the expressed proteins. Gels were run at 140 V for 1 h, stained with Quick Staining solution (850 mL H_2O, 50 mL EtOH, 80 mL phosphoric acid, 110 mg Coomassie Blue G-250, 10 g β-cyclodextrin) for 24 h and destained with deionized water for 24 h.

Synthesis of DMAPP.

Chemical synthesis of dimethylallyl pyrophosphate was performed based on literature according to Woodside *et al. Org. Synth., 1988 and Baubakir et al. Phytochemistry, 2005*. The batch size of the nucleophilic substitution of dimethylallyl bromide by pyrophosphate was increased six-fold (from 5 mmol dimethylallyl bromide and 10 mmol pyrophosphate to 30 mmol and 60 mmol, respectively). The amounts of solvent (MeCN) for the nucleophilic substitution reaction and ion exchange resin (Dowex 50W-X8) for obtaining the final ammonium form of DMAPP were doubled, affording quantitative conversion to DMAPP with only traces of remaining tetrabutylammonium ions. Hence, a final purification by column chromatography was avoided, and up to 9 g of DMAPP were obtained in one batch of synthesis.

In detail: Sodium pyrophosphate (13.8 g, 62 mmol) was dissolved in H_2O (50 mL) together with aqueous NH_3 (25% in H_2O, 1 mL) to give a colorless solution. A chromatography column (Ø = 2 cm) was packed with Dowex 50W-X8 cation exchange resin (H^+ form, 100–200 mesh) to a height of 30 cm, and the column was rinsed with H_2O (200 mL) before loading the colorless pyrophosphate solution onto the column. Elution was performed with H_2O and gravity flow. The first 250 mL of eluate were collected in a graduated cylinder. After transfer into a round-bottom flask (500 mL), the solution was titrated to pH 7.3 using aq. tetrabutylammonium hydroxide solution (40% in H_2O). The solution was flash-frozen with liquid nitrogen and lyophilized overnight. The remaining white syrup was re-dissolved in MeCN (25 mL, HPLC-grade),

evaporated under reduced pressure (40 °C water bath), flash-frozen and lyophilized a second time. Roughly 60 g of white powdery solid were obtained and stored in a desiccator over $CaCl_2$. As a next step, the pyrophosphate salt from the 1st step (57.4 g, 60.0 mmol) was weighed into a round-bottom flask and dissolved in MeCN (anh., 30 mL). 3,3-dimethylallylbromide (4.5 g, 30.0 mmol, 3550 µL) was volumetrically added to the stirring solution. The flask was flushed with nitrogen and the reaction was stirred at room temperature for 4 h. After full conversion of dimethylallyl bromide was checked by TLC (silica gel, 1-PrOH/NH_4OH/H_2O = 9/3/1), the solution was concentrated under reduced pressure (40 °C water bath). The remaining yellowish oil (20 g) was stored at 4 °C. Remaining tetrabutylammonium ions were exchanged by another ion-exchange chromatography column. To this end, the pale yellowish oil (20 g) was dissolved in ion-exchange buffer (5 mL, NH_4HCO_3 2 g/L in 2 % (v/v) isopropyl alcohol / water) and the resulting mixture was loaded onto a chromatography column (Ø = 5 cm), which was packed with Dowex 50W-X8 cation exchange resin (ammonium form, 100–200 mesh). For preparation of ammonium form, the resin (188 mL, H^+ form) was placed in a fritted glass funnel (1 L) and washed with four portions of concentrated aq. ammonia (200 mL). Afterwards the resin was washed with eight portions of H_2O (each 200 mL), to set a pH of the filtrate of 7. The washed resin was additionally washed with two portions of ion exchange buffer (200 mL) and resuspended in another portion of ion exchange buffer (200 mL). The yellowish oil was loaded onto the column, and the flask was washed with two portions of ion exchange buffer (5 mL), which were loaded on the column as well. Elution was performed with ion-exchange buffer (350–400 mL). The eluent was collected in round-bottom flasks (2 × 500 mL), flash-frozen with liquid nitrogen and lyophilized for 18 h giving 8.9 g (>99%) of product. No further purification was necessary. R_f (silica gel, 1-PrOH/NH_4OH/H_2O = 8/5/2, $KMnO_4$ staining): 0.38; ^{1}H NMR (500 MHz, D_2O): δ = 5.30 (1 H, t, J = 7.2 Hz, CH), 4.31 (2 H, t, J = 6.9 Hz, CH_2), 1.71 (s, CH_3), 1.67 (s, CH_3).

Characterization of Side Reaction.

Prenylation of L-abrine (1f) by DmaW preparation [CFE of *E. coli* BL21(DE3)], Filtered CFE and Retained Solution.

Activity assays (1 mL) with different DmaW preparations [CFE of *E. coli* BL21(DE3)] containing L-abrine (**1f**, 5 mM) as prenyl acceptor, DMAPP (5 mM), DMSO (5% v/v) and Tris/HCl buffer (50 mM pH 7.5, 5 mM $CaCl_2$) were incubated at 30 °C for 24 h. To this end, the

CFE was dissolved in Tris/HCl buffer and filtered through 10, 30 or 100 kDa MWCO centrifugal filters or a PD10 column. For assays, the non-filtered solution, the filtrate as well as the retained solution were investigated. After 0, 60 min and 24 h reaction samples were taken (150 µL) and quenched with methanol or acetonitrile (150 µL, containing 2 mM of propiophenone as internal standard) and after vigorous shaking (10 sec) analyzed on HPLC-UV.

Abrine Background Studies – "Empty" *E. coli* BL21(DE3).

Reactions (1 mL) containing either L-abrine (**1f,** 5 mM) or no prenyl acceptor, DMAPP (5 mM) and cell-free extract of *E. coli* BL21(DE3), which was transformed with pET28a(+) containing no DMATS, in Tris/HCl buffer (50 mM pH 7.5, 5 mM $CaCl_2$) were incubated at 30 °C for 24 h. Samples taken after 0, 60 min and 24 h (150 µL) were quenched with acetonitrile (150 µL, containing 2 mM of propiophenone as internal standard) and after vigorous shaking (10 sec) analyzed on HPLC-UV.

Determination of enantiomeric excess of D-1f and D-1g

Reactions for the determination of enantiomeric excess (*ee*) with tryptophan derivatives *rac*-**1f** and *rac*-**1g** (5 mM) as prenyl acceptors were set up on 5 mL scale, using DmaW wild-type [CFE of *E. coli* BL21(DE3), 100 mg] and DMAPP (5 mM). The reactions were incubated at 30 °C, 120 rpm for 24 h. Reactions were quenched with acetonitrile (5 mL) and after intensive vortexing centrifuged for 20 min. The remaining clear solutions were subjected to preparative HPLC for isolation of the remaining substrate **1f** and **1g**. Fractions containing the remaining substrate **1f** and **1g** were directly analyzed by HPLC-MS using an Astec Chirobiotic T column (25 cm x 4.6 mm, 5 µm particle size) for chiral analysis.

Section S2. Supporting Data

BLAST search

BLAST search using the amino acid sequence of DmaW from *A. japonicus* as query sequence

Table S5: Hits of BLAST search with an identity of >55% to the sequence from *A. japonicus*.

Origin of DMATS / Scientific Name	Identity	Accession code
Aspergillus indologenus CBS 114.80	100.00%	PYI33858.1
Aspergillus hancockii	70.50%	KAF7589021.1
Aspergillus leporis	69.91%	KAB8073422.1
Trichophyton rubrum	69.83%	OAL67630.1
Trichophyton rubrum	69.83%	KMQ42995.1
Trichophyton rubrum MR850	69.48%	EZF25380.1
Trichophyton equinum CBS 127.97	69.36%	EGE05316.1
Microsporum canis CBS 113480	69.25%	XP_002846186.1
Aspergillus viridinutans	69.11%	XP_043128339.1
Trichophyton benhamiae CBS 112371	68.82%	DAA79841.1
Trichophyton violaceum	68.67%	OAL70943.1
Aspergillus felis	68.67%	KAF7181299.1
Aspergillus leporis	68.47%	KAB8071281.1
Aspergillus fumigatus Z5	68.40%	KMK61240.1
Aspergillus fumigatus	68.40%	KAH3007265.1
***Aspergillus fumigatus* var. RP-2014**	**68.40%**	**KEY80409.1**
Trichophyton interdigitale	68.36%	KAG5205993.1
Trichophyton tonsurans CBS 112818	68.36%	EGE00006.1
Trichophyton interdigitale H6	68.36%	EZF33069.1
***Trichophyton benhamiae* CBS 112371**	**68.34%**	**XP_003017766.1**
Trichophyton verrucosum HKI 0517	68.28%	XP_003024001.1
Trichophyton mentagrophytes	68.13%	GBF63857.1
Aspergillus fumigatiaffinis	68.06%	KAF4211874.1
Aspergillus lentulus	68.06%	GFF56762.1
Arthroderma uncinatum	67.90%	XP_033410092.1
Aspergillus fumigatiaffinis	67.80%	KAF4221877.1
Paecilomyces variotii No. 5	67.68%	GAD93029.1
Aspergillus lentulus	67.59%	XP_033419696.1
Aspergillus lentulus	67.59%	KAF4168532.1
Aspergillus lentulus	67.59%	GAQ03949.1
Aspergillus lentulus	67.36%	KAF4207528.1
Aspergillus fumigatus	67.36%	KAH1365663.1
Hypoxylon sp. EC38	67.30%	OTA60880.1
Aspergillus fumigatus	67.10%	KAH1303997.1
Aspergillus hiratsukae	66.82%	KAF7114973.1
Aspergillus steynii IBT 23096	66.51%	XP_024702495.1
Aspergillus hancockii	66.40%	KAF7588835.1
Aspergillus fumigatus Af293	66.37%	XP_756141.2
Epichloe elymi	65.48%	AFU34392.1

Epichloe brachyelytri	65.24%	AET10057.1
Penicillium roqueforti	65.13%	XP_038930379.1
Penicillium roqueforti FM164	65.13%	W6QIM8.1
Monosporascus *sp.* CRB-9-2	65.10%	RYP93155.1
Epichloe coenophiala	64.76%	AQR55712.1
Epichloe sp. FaTG-4	64.76%	AQR55707.1
Epichloe coenophiala	64.76%	Q6X2E2.1
Epichloe *sp.* E4305	64.76%	AGS32000.1
Aspergillus coremiiformis	64.75%	KAE8348771.1
Penicillium camemberti	64.61%	CRL19777.1
Epichloe bromicola	64.52%	ATJ44712.1
Epichloe glyceriae	64.52%	AET10048.1
Epichloe sp. FaTG-5	64.52%	AQR55705.1
Penicillium sp. YT-2016	64.51%	AMQ76113.1
Malbranchea aurantiaca	**64.39%**	**ABZ80611.1**
Penicillium griseofulvum	64.35%	XP_040647200.1
Epichloe cabralii	64.18%	AII23274.1
Epichloe amarillans	64.05%	AGS31969.1
Epichloe cabralii	63.93%	AII23275.1
Aspergillus homomorphus CBS 101889	63.92%	XP_025554348.1
Aspergillus fumigatus Af293	63.83%	Q50EL0.2
Aspergillus fumigatus	63.83%	AAX08549.1
Epichloe canadensis	63.81%	AGS31978.1
Epichloe mollis	63.81%	AGS31988.1
Epichloe typhina	63.81%	AEV21238.1
Penicillium expansum	63.64%	XP_016600812.1
Epichloe coenophiala	63.57%	AQR55717.1
Daldinia sp. EC12	63.51%	OTB14167.1
Aspergillus fumigatus	63.45%	3I4X_A
Periglandula sp. 1 WB-2014	63.37%	AJO54608.1
Epichloe sp. FaTG-2	63.33%	AQR55709.1
Epichloe sp. FaTG-2	63.33%	AQR55711.1
Epichloe festucae var. lolii	63.33%	AQR55704.1
Epichloe coenophiala	63.33%	Q6X2E1.1
Periglandula sp. 3 WB-2014	63.13%	AJO54614.1
Periglandula sp. 3 WB-2014	63.13%	AJO54604.1
Epichloe festucae	63.10%	AET11901.1
Epichloe coenophiala	63.04%	AQR55719.1
Periglandula sp. 3 WB-2014	62.89%	AJO54600.1
Periglandula sp. 3 WB-2014	62.89%	AJO54602.1
Periglandula sp. 3 WB-2014	62.89%	AJO54605.1
Periglandula sp. 1 WB-2014	62.89%	AJO54609.1
Periglandula sp. 3 WB-2014	62.65%	AJO54601.1
Periglandula sp. 1 WB-2014	62.65%	AJO54607.1
Aspergillus fumigatus	62.55%	KAH1435415.1
Periglandula sp. 3 WB-2014	62.41%	AJO54603.1

Penicillium steckii	62.39%	OQE13746.1
Clavicipitaceae sp. US2005a	62.38%	AAZ29613.1
Glarea lozoyensis ATCC 20868	62.00%	XP_008079535.1
Metarhizium robertsii	61.75%	EXU97513.1
Metarhizium acridum CQMa 102	61.45%	XP_007813329.1
Metarhizium anisopliae	61.00%	KAF5137822.1
Clavicipitaceae sp. US2005b	60.98%	AAZ29614.1
Daldinia childiae	60.89%	XP_033438065.1
Aspergillus fumigatus	60.61%	KAH1479965.1
Aspergillus fumigatus	60.61%	KAH1325236.1
Balansia obtecta	60.42%	Q6X1E1.1
Aspergillus fumigatus	60.38%	KAH1320154.1
Aspergillus fumigatus	60.38%	KAH1306301.1
Trichoderma arundinaceum	60.32%	RFU74759.1
Claviceps purpurea	60.22%	UFQ30683.1
Nemania sp. FL0031	60.19%	KAI0103178.1
Claviceps purpurea	60.11%	UFQ30643.1
Claviceps purpurea	60.11%	UFQ30725.1
Aspergillus fumigatus	59.91%	OXN07853.1
Aspergillus fumigatus	59.67%	KAF4283057.1
Aspergillus fumigatus	59.67%	KAF4257043.1
Aspergillus fumigatus	59.67%	KAF4267498.1
Epichloe inebrians	59.58%	AFO67567.1
Claviceps purpurea	59.46%	UFQ30717.1
Claviceps cyperi	59.33%	UFQ30732.1
Glarea lozoyensis 74030	59.32%	EHK99795.1
Xylaria telfairii	59.22%	KAI0436161.1
Claviceps purpurea	58.69%	KAG6124472.1
Claviceps purpurea	58.69%	KAG6203129.1
Bisporella sp. PMI_857	58.57%	KAH8599572.1
Claviceps purpurea	58.51%	UFQ30714.1
Claviceps monticola	58.47%	UFQ30628.1
Bisporella sp. PMI_857	58.41%	KAH8600507.1
Claviceps purpurea	58.29%	KAG6175777.1
Claviceps purpurea 20.1	58.27%	M1WA41.1
Claviceps purpurea	58.27%	UFQ30693.1
Claviceps purpurea	58.27%	UFQ30692.1
Claviceps purpurea	58.27%	UFQ30705.1
Claviceps purpurea	58.03%	UIO60788.1
Claviceps purpurea	58.03%	AAP81209.1
Claviceps purpurea	58.03%	P0CT20.1
Claviceps purpurea	58.03%	UFQ30706.1
Claviceps purpurea	57.95%	UFQ30694.1
Claviceps sp. LM218 group G6	57.85%	KAG6081729.1
Claviceps purpurea	57.79%	UFQ30685.1
Claviceps purpurea	57.79%	UFQ30703.1

Claviceps purpurea	57.79%	UFQ30709.1
Claviceps purpurea	57.61%	UFQ30637.1
Claviceps sp. LM219 group G6	57.61%	KAG6113331.1
Claviceps purpurea	57.52%	UFQ30659.1
Claviceps purpurea	57.52%	UFQ30666.1
Claviceps perihumidiphila	57.38%	UFQ30701.1
Claviceps sp. LM220 group G6	57.38%	KAG6092037.1
Claviceps sp. LM454 group G7	57.38%	KAG6102183.1
Claviceps cyperi	57.34%	KAG5963720.1
Claviceps monticola	57.33%	KAG5937931.1
Claviceps aff. purpurea	57.33%	KAG6298805.1
Claviceps purpurea	**57.29%**	**CAC37397.1**
Claviceps spartinae	57.14%	KAG5988862.1
Claviceps paspali	57.14%	AET79202.1
Claviceps humidiphila	57.11%	UFQ30639.1
Claviceps arundinis	57.11%	UFQ30733.1
Claviceps purpurea	57.07%	KAG6165195.1
Claviceps purpurea	57.07%	KAG6252860.1
Claviceps purpurea	57.07%	KAG6236345.1
Claviceps capensis	57.07%	KAG5912733.1
Claviceps purpurea	57.04%	UFQ30658.1
Claviceps arundinis	56.88%	UFQ30630.1
Claviceps quebecensis	56.85%	UFQ30690.1
Claviceps purpurea	56.83%	UFQ30707.1
Claviceps purpurea	56.80%	KAG6278115.1
Claviceps purpurea	56.80%	KAG6187076.1
Claviceps purpurea	56.80%	KAG6195066.1
Claviceps purpurea	56.80%	KAG6156876.1
Claviceps purpurea	56.80%	KAG6319203.1
Claviceps purpurea	56.80%	KAG6170825.1
Claviceps purpurea	56.76%	KAG6247998.1
Claviceps sp. LM458 group G5	56.72%	KAG6022592.1
Claviceps purpurea	56.59%	UFQ30646.1
Claviceps purpurea	56.53%	KAG6196366.1
Claviceps purpurea	56.53%	KAG6239811.1
Claviceps purpurea	56.53%	KAG6223227.1
Claviceps purpurea	56.53%	KAG6141982.1
Claviceps purpurea	56.53%	KAG6183545.1
Claviceps purpurea	56.53%	KAG6229852.1
Claviceps purpurea	56.50%	KAG6178302.1
Claviceps aff. humidiphila group G2b	56.46%	KAG6062460.1
Claviceps quebecensis	56.39%	UFQ30724.1
Claviceps sp. LM77 group G4	56.36%	KAG6056459.1
Claviceps purpurea	56.27%	KAG6140329.1
Claviceps purpurea	56.27%	KAG6267624.1
Claviceps aff. purpurea	56.27%	KAG6286574.1

Claviceps purpurea	56.12%	KAG6318267.1
Claviceps purpurea	56.10%	KAG6135791.1
Claviceps aff. humidiphila group G2b	55.97%	KAG6058163.1
Claviceps perihumidiphila	55.97%	UFQ30654.1
Claviceps purpurea	55.96%	UFQ30720.1
Claviceps sp. Clav32 group G5	55.84%	KAG6033503.1
Claviceps purpurea	55.73%	KAG6273628.1
Claviceps purpurea	55.73%	KAG6165429.1
Claviceps purpurea	55.60%	UFQ30728.1
Claviceps humidiphila	55.56%	KAG6117442.1
Claviceps arundinis	55.56%	KAG5965194.1
Claviceps arundinis	55.30%	KAG5951803.1
Claviceps monticola	55.17%	KAG5934042.1
Claviceps sp. LM84 group G4	55.17%	KAG6078752.1
Claviceps sp. LM77 group G4	55.17%	KAG6057682.1
Claviceps pazoutovae	55.05%	UFQ30632.1

Estimation of enzyme content in the DmaW preparation [CFE of *E. coli* BL21(DE3)]

An estimation of pure DmaW content per mg of DmaW preparation [CFE of *E. coli* BL21(DE3)] was made by comparison of the initial reaction rate (consumption of L-**1a** over 30 min) in biotransformations using 20 mg/mL of CFE or different concentrations of purified DmaW. Thereby, 20 mg/mL DmaW preparation [CFE of *E. coli* BL21(DE3)] revealed an initial rate roughly equivalent in activity to 13 µM of pure enzyme (Figure S3). Hence an estimation of 0.65 µM of pure DMATS per mg of CFE could be made.

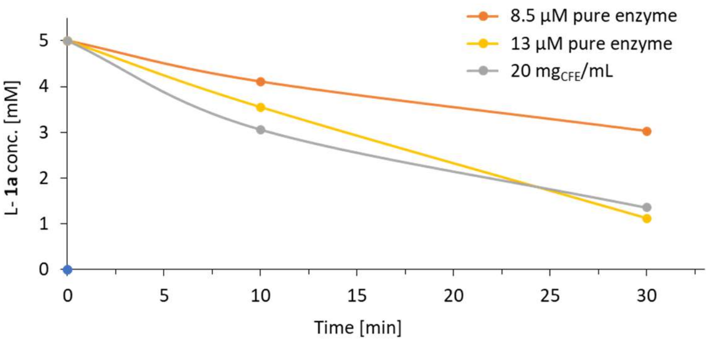

Figure S3: Comparison of L-**1a** (5 mM) consumption in 30 min by purified enzyme (8.5 and 13 µM) and crude DmaW preparation [CFE of *E. coli* BL21(DE3)] (20 mg_{CFE}/mL). Reaction conditions: L-**1a** (5 mM), DMAPP (5 mM), by purified enzyme (8.5 or 13 µM) or DmaW

preparation [CFE of *E. coli* BL21(DE3)] (20 mg_{CFE}/mL), Tris/HCl buffer (50 mM pH 7.5, 5 mM $CaCl_2$), 30 °C, 24 h.

Effect of substrate concentration and DMSO addition on the prenylation of tryptophan derivatives by DmaW from *A. japonicus*

The optimum ratio of prenyl donor (DMAPP) to prenyl acceptor (L-**1a**) was assessed using 1 mM of L-**1a**, 1–4 mM of DMAPP, and 10, 20 or 40 mg_{CFE}/mL reaction volume by checking the conversion after 1 h and 24 h. An equimolar ratio of L-**1a** and DMAPP gave the highest conversion after 1 h (25–38%), decreasing with increase of DMAPP concentration to 19–29% for a 1:2 ratio and 10–23% for a 1:4 ratio of L-**1a** and DMAPP. No significant difference was found with investigation of 20 or 40 mg_{CFE}/mL (Figure S4). After 24 h, in all investigated setups quantitative conversion could be obtained. Therefore, a 1:1 ratio of prenyl donor and prenyl acceptor was used in all further reactions with DMATS.

The effect of DMSO concentration on the C4-prenylation activity of DmaW was studied. For the determination of the DMSO optimum, the consumption of substrates L-**1a,** D-**1a,** L-**1c,** *rac*-**1d,** L-**1f** and L-**1g** in 60 min in prenylation by 20 mg/mL DmaW preparation [CFE of *E. coli* BL21(DE3)] was measured. The conversion data were transformed into a the rate (in U/g_{CFE}), which was found to be highest at 5– 10% v/v DMSO for most substrates (Figure S5). Despite the cosolvent addition, completely homogeneous reaction mixtures could not be obtained in some cases (*e.g.*, L-**1f** and L-**1g**), leading to large standard deviations. However, as addition of 5% v/v DMSO to the reaction mixtures gave overall the best results in prenylation of tryptophan derivatives, all further reactions were supplemented with this amount of cosolvent.

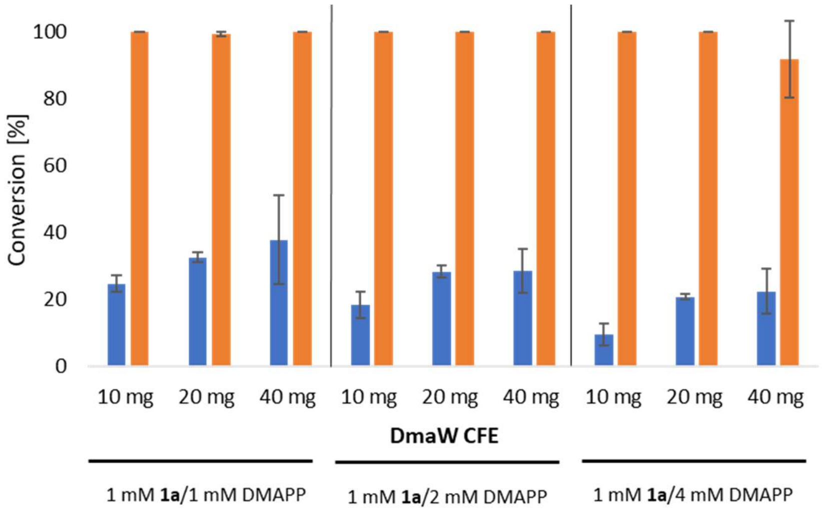

Figure S4: Conversion of L-**1a** (1 mM) into L-**2a** by DmaW preparation [CFE of *E. coli* BL21(DE3)] (10, 20 and 40 mg_{CFE}/mL, corresponding to 6.5, 13 and 26 µM pure DmaW), after 1 h (blue) and 24 h (orange) of reaction, investigating 10–40 mg/mL CFE and either 1:1, 1:2 or 1:4 ratio of prenyl acceptor (L-**1a**) and prenyl donor. Reaction conditions: L-**1a** (1 mM), DMAPP (1–4 mM), Tris/HCl buffer (50 mM pH 7.5, 5 mM $CaCl_2$), 30 °C, 1–24 h.

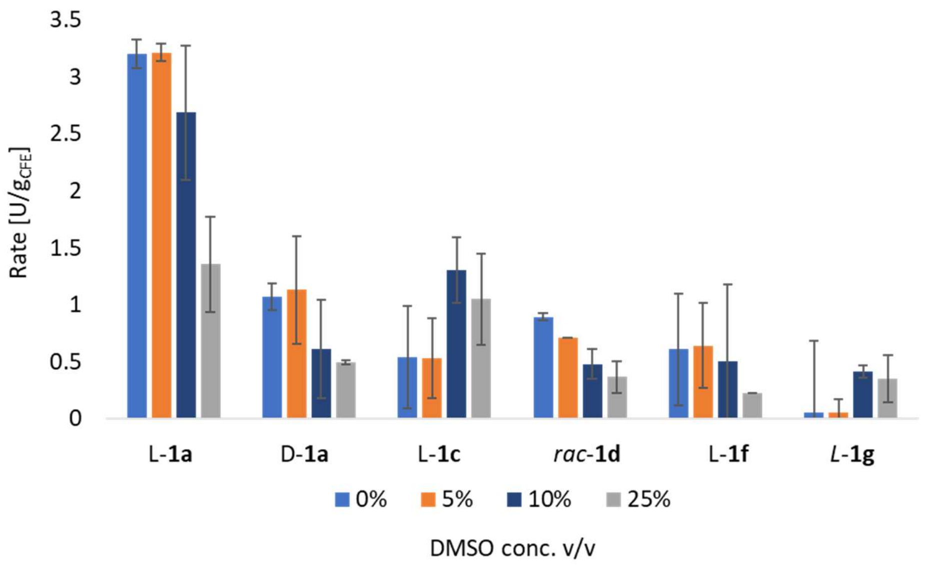

Figure S5: Rates of prenylation of tryptophan derivatives L-**1a,** D-**1a,** L-**1c,** *rac*-**1d,** L-**1f** and L-**1g** (5 mM) within the first 60 min of reaction using 20 mg/mL DmaW preparation [CFE of *E. coli* BL21(DE3)] and 0–25 % v/v DMSO. Reaction conditions: tryptophan derivative (5 mM), DMAPP (5 mM), DmaW preparation [CFE of *E. coli* BL21(DE3)] (20 mg_{CFE}/mL, corresponding to 13 µM pure DmaW), Tris/HCl buffer (50 mM pH 7.5, 5 mM $CaCl_2$), DMSO (0–25 % v/v), 30 °C, 60 min.

Test for product inhibition

The product L-**2a** was added to the prenylation reaction at varied concentration (Figure S6). Until 5 mM of initial product concentration present no effect was observed. 10 and 20 mM of the prenylated product L-**2a** led to slightly reduced conversions of 90 and 75%, respectively.

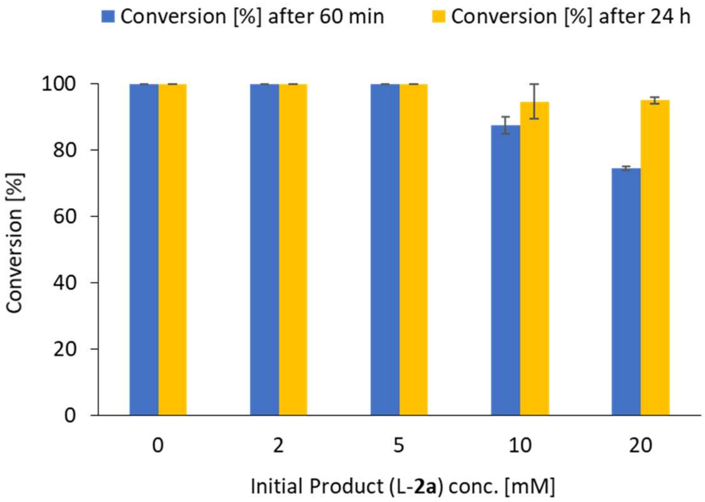

Figure S6: Investigation of product inhibition in the prenylation of L-**1a** by DmaW. Conversion of L-**1a** after 60 min (blue) and 24 h (yellow) at varied initial product concentration L-**2a** (0, 2, 5, 10 and 20 mM). Reaction conditions: L-**1a** (5 mM), DMAPP (5 mM), L-**2a** (0–20 mM), DmaW preparation [CFE of *E. coli* BL21(DE3),] (20 mg$_{CFE}$/mL, corresponding to 13 µM pure DmaW), Tris/HCl buffer (50 mM, pH 7.5, 5 mM CaCl2), 30 °C, 24 h.

Blank studies with DmaW preparation [CFE of *E. coli* BL21(DE3)]

To identify the background resulting from *E. coli* CFE, blank reactions containing either only the DmaW CFE-preparation, or the substrate L-**1a** or the substrate L-**1a** and DMAPP, were analyzed by HPLC-UV. Several peaks in the area between 2.0 and 6.0 min of retention time were assigned to the CFE. Reactions without CFE, but L-**1a** and DMAPP showed no additional peaks in this retention time range with UV detection at 262 nm (Figure S7).

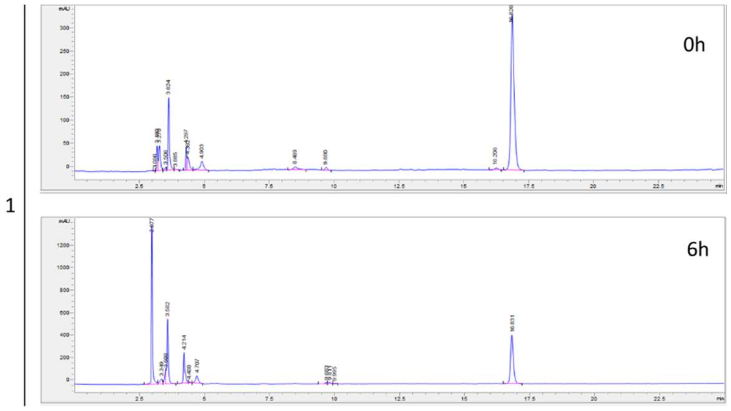

2

3

4

5

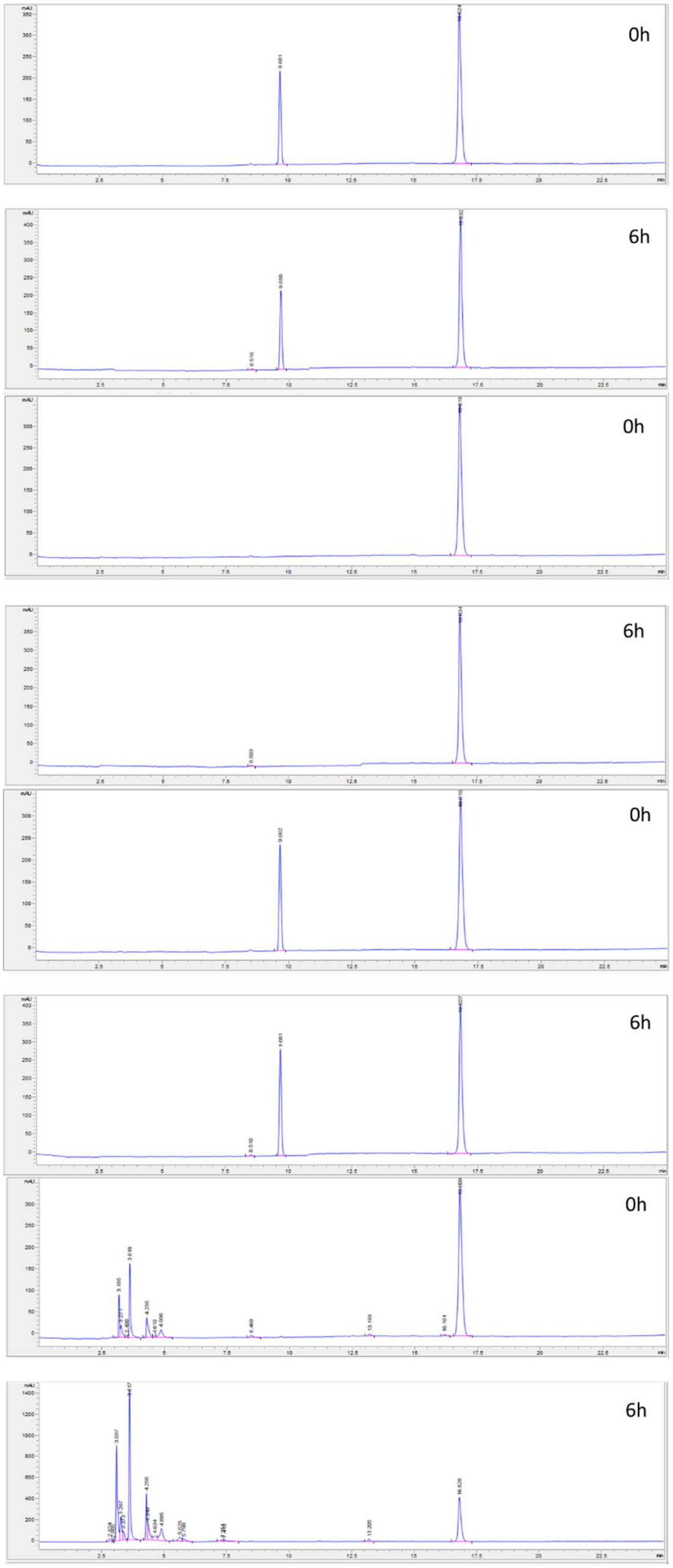

6

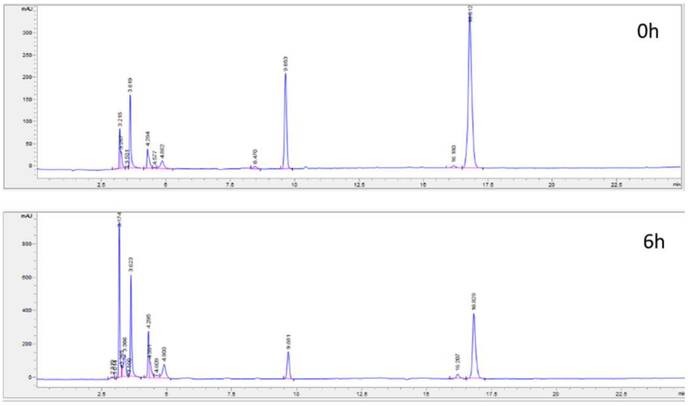

Figure S7: Blank studies to determine background peaks (additional peaks, which neither can attributed to the substrate nor the product) using DmaW preparation [CFE of *E. coli* BL21(DE3)] and Tris/HCl buffer (50 mM, pH 7.8, 5 mM $CaCl_2$). 1: DmaW preparation [CFE of *E. coli* BL21(DE3)] (20 mg_{CFE}/mL, corresponding to 13 μM pure DmaW), Tris/HCl buffer (50 mM pH 7.5, 5 mM $CaCl_2$), internal standard 2-propiophenone, 30 °C, 120 rpm, 0 and 6h; 2: L-**1a** (5 mM), Tris/HCl buffer (50 mM pH 7.5, 5 mM $CaCl_2$), 30 °C, 120 rpm, 0 and 6 h; 3: DMAPP (5 mM), Tris/HCl buffer (50 mM pH 7.5, 5 mM $CaCl_2$), 30 °C, 120 rpm, 0 and 6 h; 4: L-**1a (**5 mM), DMAPP (5 mM), Tris/HCl buffer (50 mM pH 7.5, 5 mM $CaCl_2$), 30 °C, 120 rpm, 0 and 6 h; 5: DmaW preparation [CFE of *E. coli* BL21(DE3)] (20 mg_{CFE}/mL, corresponding to 13 μM pure DmaW), DMAPP (5 mM), Tris/HCl buffer (50 mM pH 7.5, 5 mM $CaCl_2$), 30 °C, 120 rpm, 0 and 6 h; 6: DmaW preparation [CFE of *E. coli* BL21(DE3)] (20 mg_{CFE}/mL, corresponding to 13 μM pure DmaW), L-**1a (**5 mM), Tris/HCl buffer (50 mM pH 7.5, 5 mM $CaCl_2$), 30 °C, 120 rpm, 0 and 6 h.

Tryptophan content in *E. coli* BL21(DE3) CFE

Since crude CFE was used as biocatalyst, the amino acid L-**1a** was likely present. The tryptophan concentration in the CFE of *E. coli* BL21(DE3) was determined by HPLC-UV and was found to be in the range of 0.25 mM at 20 mg/mL CFE.

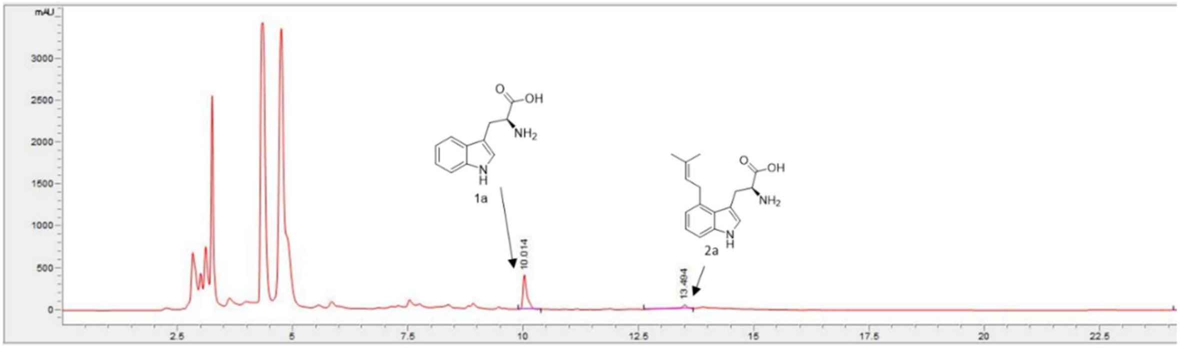

Figure S8: HPLC-UV chromatogram of 200 mg/mL CFE of *E. coli* BL21(DE3) without DmaW in Tris/HCl buffer (50 mM pH 7.5, 5 mM $CaCl_2$), indicating the presence of L-**1a** and trace amounts of L-**2a**.

Sequence alignment of DMATSs from *A. japonicus*, *C. purpurea* and *T. benhamiae*

An amino acid sequence alignment of all three investigated DMATSs from *A. japonicus*, *T. benhamiae* and *C. purpurea* was performed using the Benchling sequence alignment tool (Figure S9). Some conserved regions (highlighted in dark) within the active site (T108 – T208), but also within the whole enzyme were identified.

```
T.benhamiae    1 MGSI----EIPNCSGSIVYKTISDFIDFPNHEQKLWWHSTAPMFAEMLRVAGYDLHSQYK
C.purpurea     1 MSTA----KD---PGNGVYEILSLIFDFPSNEQRLWWHSTAPMFAAMLDNAGYNIHDQYR
A.japonicus    1 MGTAGQGIKTGNASDCEVYRTLSVALDFANQDEELWWHSTAPMFAQMLQSTNYNLHAQYK

T.benhamiae   57 ILGIFLNHVIPFLGVYPTRINNRWLSILTRYGTPFELSLNCSQSLVRYTYEPINSATGTV
C.purpurea    54 HLGIFKKHIIPFLGVYPTKDKERWLSILTRCGLPLELSLNCTDSVVRYTYEPINEVTGTE
A.japonicus   61 HLLIYKKNVIPFLGVYPTNDKPRWLSILTRYGTPFELSLNCSGPLVRYTYEPINAATGTA

T.benhamiae  117 KDPFNTHSIWDALDRLMPLQKGIDLEFFKHLKQDLTVDDQDSAYLLENNLVGGQIRTQNK
C.purpurea   114 KDTFNTLAIMTSVQKLAQIQAGIDLEWFSYFKDELTLDESESATLQSNELVKEQIKTQNK
A.japonicus  121 RDPFNTHAVWDSLEQLMALQSGIDLDLFRHFKNDLTLSAEESEYLYKNNLVGEQIRTQNK

T.benhamiae  177 LALDLKGGNFVLKTYIYPALKALATGKSIKTLMFDSVYRLCRQNPSLEAPLRALEEYVDS
C.purpurea   174 LALDLKESQFALKVYFYPHLKSIATGKSTHDLIFDSVLKLSQKHDSIQPAFQVLCDYVSR
A.japonicus  181 LALDLQDGEFVVKTYIYPALKSLATGRSIHELVFGSAFRWSKQYPELRKPLDTLEQYVYS

T.benhamiae  237 KGPNST------ASPRLLSCDLIDPSKSRVKIYILELNVTLEAMEDLWTMGGRLNDASTL
C.purpurea   234 RNHSAEVDQHGALHARLLSCDLIDPAKSRVKIYLLEKTVSLSVMEDLWTLGGQRVDASTM
A.japonicus  241 RGPSST------ASPRLLSCDLIDPTKSRIKIYLLERMVTLEALEDLWTMGGERTDASTL

T.benhamiae  291 AGLEMLRELWDLIKLPPGMREYPEPFLQLGTIPDEQLPLMANYTLHHDQAMPEPQVYFTT
C.purpurea   294 DGLDMLRELWSLLKVPTGHLEYPKGYLELGEIPNEQLPSMANYTLHHNDPMPEPQVYFTV
A.japonicus  295 AGLEMIRELWELIRLPAGLQSYPAPYLPIGTIPDEQLPLMANYTIHHDDPVPEPQVYFTT

T.benhamiae  351 FGLNDGRIADGLVTFFERRGWNHMAQTYKDSLRAYYPHADQETLNYLHAYISFSYRKGKP
C.purpurea   354 FGMNDAEISNALTIFFQRHGFDDMAKNYRVFLQDSYPYHDFESLNYLHAYISFSYRRNKP
A.japonicus  355 FGRNDMQIADALATFFERRGWHEMARQYKAELCSHYPHADHETLNYLHAYISFSYRKNKP

T.benhamiae  411 YLSVYLQTFETGDWPISNFGIPVVKP-LRSNVGCQHPI----SFSIPITKAPLLGV----
C.purpurea   414 YLSVYLHTFETGRWPVFADSPISFDAYRRC------EL---------STK----------
A.japonicus  415 YLSVYLQSLETGDWVTSSFNSVHVDPGLSATVQELSKLTKTAGTTVRETKLPLTPDGSEP

T.benhamiae  462 -----A
C.purpurea       ------
A.japonicus  475 GVITQY
```

Figure S9: Sequence alignment of DMATSs from *T. benhamiae*, *C. purpurea* and *A. japonicus* generated using the Benchling sequence alignment tool. Amino acid positions conserved in all three enzymes are highlighted in black.

Engineering of DmaW from *A. japonicus* / Expression of DmaW variants

The results of sequencing indicated the desired amino acid exchange in all cases and hence the plasmids were transformed into cells of the expression strain *E. coli* ArcticExpress (DE3). Successful expression of all DmaW variants was conducted in *E. coli* ArcticExpress (DE3) cells using an expression temperature of 13 °C (Figure S10).

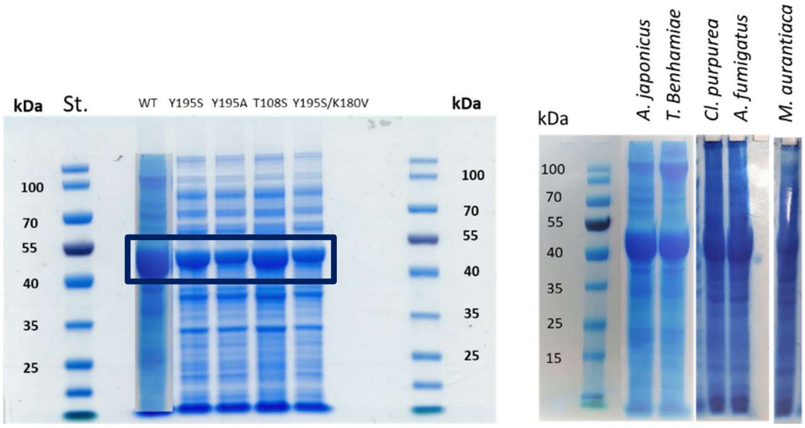

Figure S10: SDS-PAGE analysis of the successful expression trials of mutated DmaW from *A. japonicus* and alternative DMATS: Lanes show CFE after sonication containing a band in the area between 40 and 55 kDa, which corresponds to soluble DMATS. Wild type (WT) DMATS and variants are indicated above the SDS PAGE together with a molecular weight standard on the left and right end.

Products obtained in the prenylation of L-abrine (1f) by DmaW

HPLC-MS analysis of L-**1f** prenylation revealed four product peaks (t_r = 13.5 min, 13.7 min, 13.9 min and 14.1 min) with *m*/*z* = 273, *m*/*z* = 285, *m*/*z* = 287, and *m*/*z* = 283 ($M+H^+$ in all cases), in which the third one corresponds to the desired Me-DMAT. The other masses suggested a demethylation process (first peak, *m*/*z* = –14 relative to **1f**) and an oxidation (second and fourth peak, *m*/*z* –2 and –4, respectively). To further elucidate these reactions, studies with L-**1f** and various preparations of DmaW either in purified or cell-free extract form were carried out, using

either standard conditions or oxygen-free atmosphere. Blank reactions using the CFE of *E. coli* BL21(DE3) containing no DmaW were also set up. An overview of suggested reaction schemes with CFE and purified DmaW is shown in the main article (Scheme 2).

Comparison of L-**1a** and L-**1f** prenylation

One of the main products formed in the prenylation of L-**1f** using CFE and atmospheric conditions was the non-methylated DMAT (L-**2a**), which suggests that the identified demethylation processes take place much faster than prenylation of L-**1f**. The progress of prenylation and demethylation was compared between biotransformations run for 1 h and for 24 h (Figure S11). After 60 min mostly the non-methylated DMAT L-**2a** and remaining L-**1f** can be detected, whereas after 24 h no L-**1f** was left and the cyclized L-abrine prenylation product L-**4f** increased. To further elucidate the difference in reaction rate between L-**1a** and L-**1f**, separate biotransformations of either of these substrates (5 mM) were set up and the decrease in substrate concentration followed for 60 min. Using purified DmaW, roughly 40% of L-**1a** was converted to DMAT (L-**2a**) in the first 60 min, whereas conversion of L-**1f** to its prenylated analogue L-**2f** was hardly detectable (Figure S12).

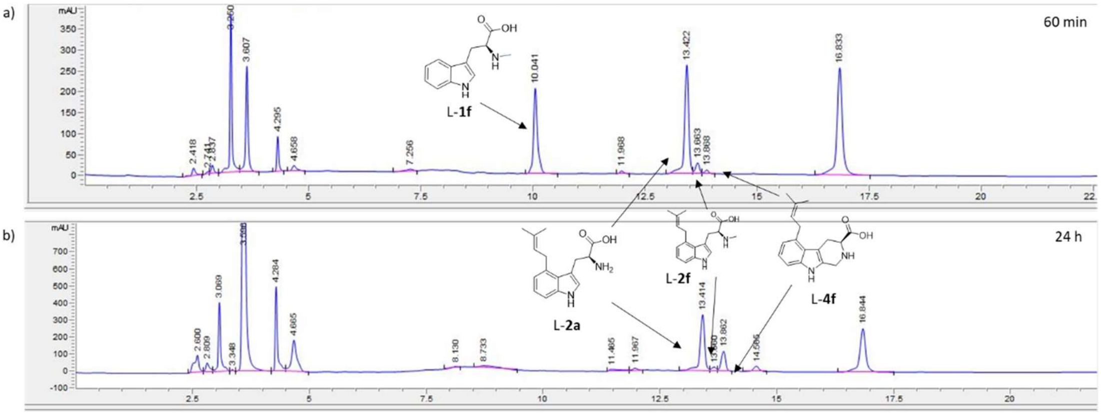

Figure S11: HPLC-UV chromatograms of L-**1f** prenylation using DmaW preparation [CFE of *E. coli* BL21(DE3)]. Reaction conditions: L-**1f** (5 mM), DMAPP (5 mM), DmaW preparation [CFE of *E. coli* BL21(DE3)] (20 mg_{CFE}/mL, corresponding to 13 µM pure DmaW), Tris/HCl buffer (50

mM pH 7.5, 5 mM $CaCl_2$), 30 °C, 120 rpm; a) after 60 min of reaction, highlighting L-**1f** at t_r = 10.04 min, L-**2a** at t_r = 13.4 min, L-**2f** at t_r = 13.6 min, L-**4f** at t_r = 13.8 min and the internal standard propiophenone at t_r = 16.8 min; b) after 24 h of reaction, highlighting L-**2a** at t_r = 13.4 min, L-**2f** at t_r = 13.6 min, L-**4f** at t_r = 13.8 min and the internal standard propiophenone at t_r = 16.8 min.

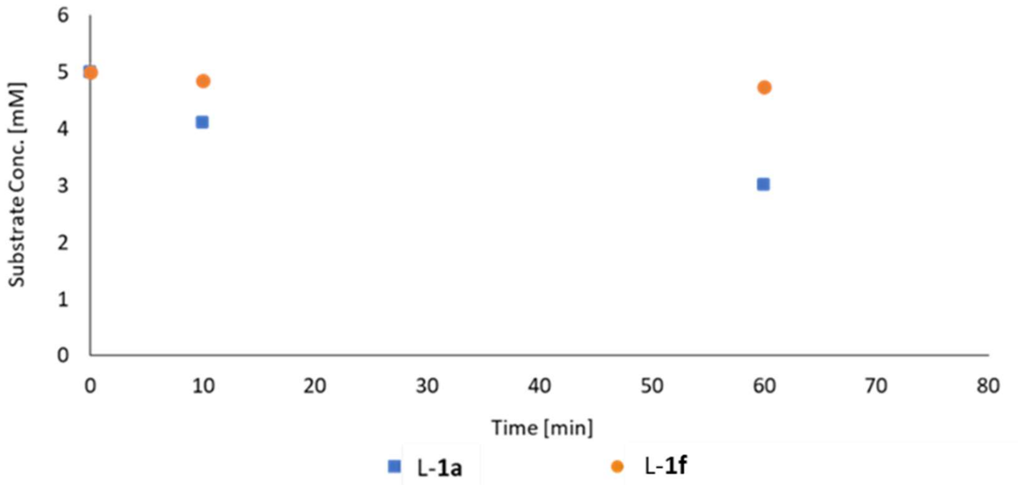

Figure S12: Substrate concentration of L-**1a** (blue) and L-**1f** (orange) at starting point, 15 min and 60 min of prenylation using purified DmaW from *Aspergillus japonicus*. Reaction conditions: L-**1a** or L-**1f** (5 mM), DMAPP (5 mM), DmaW (13 µM), Tris/HCl buffer (50 mM pH 7.5, 5 mM $CaCl_2$), 30 °C, 120 rpm, 0–60 min.

To identify the background activity of the CFE of *E. coli* BL21(DE3), reactions were performed using the CFE devoid of DMATS under atmospheric conditions. Either L-**1f** alone or L-**1f** with DMAPP as prenyl donor were used as substrate. Both setups showed separation of the substrate peak (**1f**, t_r = 10.9 min) into two (t_r = 10.5 min and 10.9 min; Figure S13), which could be assigned by HPLC-MS to the starting material L-**1f** (m/z = 219, $M+H^+$) and the demethylated product L-**1a** (m/z = 205, $M+H^+$). These results indicate that the demethylation side activity observed in DmaW-catalyzed prenylation reactions can be ascribed to *E. coli* proteins rather than DMATS.

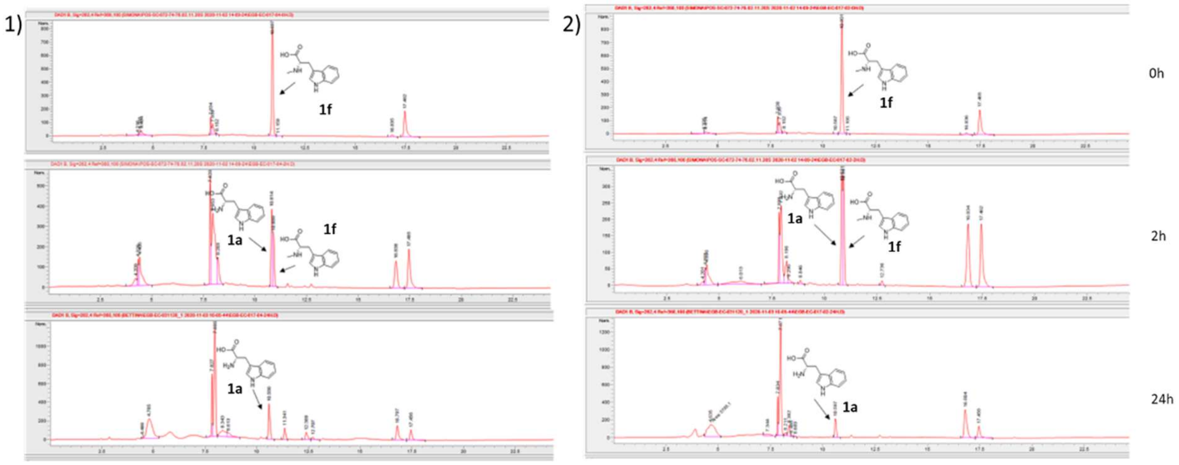

Figure S13: HPLC-UV chromatograms of blank studies using CFE of "empty" *E. coli* BL21(DE3), L-**1f**, DMAPP and Tris/HCl buffer (50 mM pH 7.5, 5 mM $CaCl_2$). 1): CFE of *E. coli* BL21(DE3) (20 mg/mL), L-**1f (**5 mM), Tris/HCl buffer (50 mM pH 7.5, 5 mM $CaCl_2$), internal standard 2-propiophenone, 30 °C, 120 rpm, 0, 2 h and 24 h. 2): DmaW preparation [CFE of *E. coli* BL21(DE3)] (20 mg_{CFE}/mL/ 13 µM pure DmaW), L-**1f** (5 mM), DMAPP (5 mM), Tris/HCl buffer (50 mM pH 7.5, 5 mM $CaCl_2$), internal standard 2-propiophenone, 30 °C, 120 rpm, 0, 2 h and 24 h.

Given the apparent oxidation reactions under atmospheric conditions using DmaW preparation [CFE of *E. coli* BL21(DE3)], prenylation was also performed under exclusion of oxygen in a glove box. Setting up the reactions in oxygen-free environment under otherwise identical conditions led mainly to the formation of L-**2f**. No oxidation product L-**4f** (m/z = 285) and only a small amount of DMAT (L-**2a**) with the retention time of 13.4 min were obtained (Figure S14; Note: The retention times are shifted ~0.5 min compared to other chromatograms. See internal standard propiophenone at 17.5 min compared to 17.0 min in other chromatograms). Moreover, with oxygen exclusion less than 50% of L-**1f** were converted, whereas under atmospheric conditions the tryptophan derivative is completely transformed to L-**2f**, L-**2a**, the cyclic oxidation product L-**4f** (m/z = 285) and another oxidation product (m/z = 283) with unknown structure. In combination with other experiments, this result points towards initial demethylation of L-**1f** to L-**1a** and subsequent prenylation of the latter. This explains the increase in conversion due to better acceptance of L-**1a** than L-**1f** by DmaW.

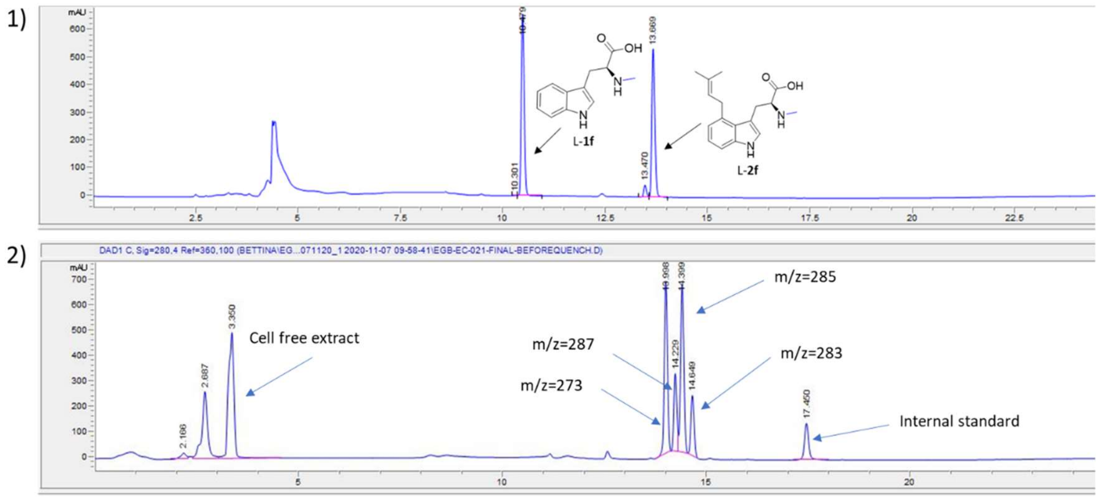

Figure S14: HPLC-UV chromatograms of L-**1f** prenylation using DmaW preparation [CFE of *E. coli* BL21(DE3)], L-**1f**, DMAPP and Tris/HCl buffer (50 mM pH 7.5, 5 mM $CaCl_2$) in the presence or absence of atmospheric oxygen. 1: HPLC-UV chromatogram of prenylation by DmaW preparation [CFE of *E. coli* BL21(DE3)], L-**1f (**5 mM), Tris/HCl buffer (50 mM pH 7.5, 5 mM $CaCl_2$), 30 °C, 120 rpm, in the absence of molecular oxygen, 24 h; 2: HPLC-UV chromatogram of prenylation by DmaW preparation [CFE of *E. coli* BL21(DE3)] (20 mg_{CFE}/mL, corresponding to 13 µM pure DmaW), L-**1f (**5 mM), Tris/HCl buffer (50 mM pH 7.5, 5 mM $CaCl_2$), 30 °C, 120 rpm, under ambient atmosphere, 24 h, highlighting the four obtained product peaks and their masses.

Investigation of purified DmaW in prenylation of L-1f

To identify the impact of CFE on prenylation reactions using L-**1f** as prenyl acceptor, additional reactions were set up with His-tag purified DmaW in the presence or absence of atmospheric oxygen. Blank reactions without enzyme gave only an L-**1f** peak after 24 h, showing no formation of the demethylated form L-**1a**. Reactions with purified enzyme revealed only the methylated DMAT (Me-DMAT, L-**2f**) with a retention time of 13.6 min, showing no oxidation product L-**4f** or demethylated form L-**2a** (Figure S15). Additionally, setups with L-**1f** and purified DmaW but without DMAPP (prenyl donor) gave no demethylation of L-**1f**. These results were equal in oxygen-containing and oxygen-free environment. Conversions to L-**2f** were around 8% for oxygen-containing (ambient) and around 12% for oxygen-free conditions. These results agree with those of blank reactions using "empty *E. coli* (see above), confirming components of the *E. coli* CFE as cause for the demethylation and oxidation side reactions.

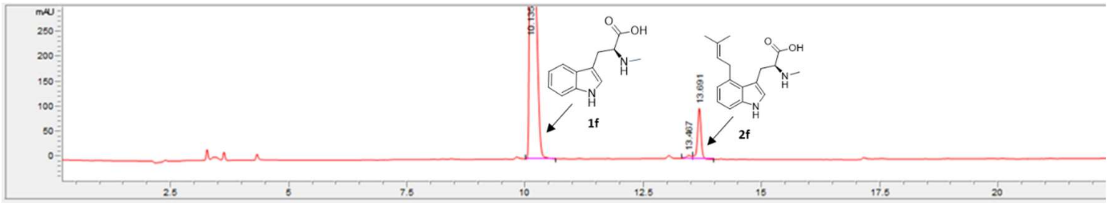

Figure S15: HPLC-UV chromatograms of L-**1f** prenylation using purified DmaW from *A. japonicus*, highlighting the formation of L-**2f** at 13.6 min and the remaining substrate peak, L-**1f** at 10.1 min. Reaction conditions: L-**1f** (5 mM), DMAPP (5 mM), purified DmaW (900 µg/mL), Tris/HCl buffer (50 mM pH 7.5, 5 mM $CaCl_2$), under ambient atmosphere.

Investigation of different CFE preparations in prenylation of L-1f

To better understand the source of demethylation and oxidation activity within the CFE of *E. coli* BL21(DE3), different preparations of the lyophilized CFE were investigated in the prenylation of L-**1f**. By using either the CFE solution directly after dissolving in Tris/HCl buffer, the flow-through after filtration with different molecular weight centrifugal filters (low molecular weight fractions = filtrate) or the remaining higher molecular weight fractions in the upper part of the centrifugal filter (= retentate), the side reaction should be traced back to components of different molecular weight. In addition, the eluate of a commercial size-exclusion chromatography column (PD10, GE Healthcare) was investigated. In Figure S16 an overview of the obtained results is given. The different colored circles indicate presence of the demethylation (orange) and cyclisation (green) side reactions as well as the prenyltransferase activity (blue). Additionally, the size of the circles reflects the abundance of the formed product.

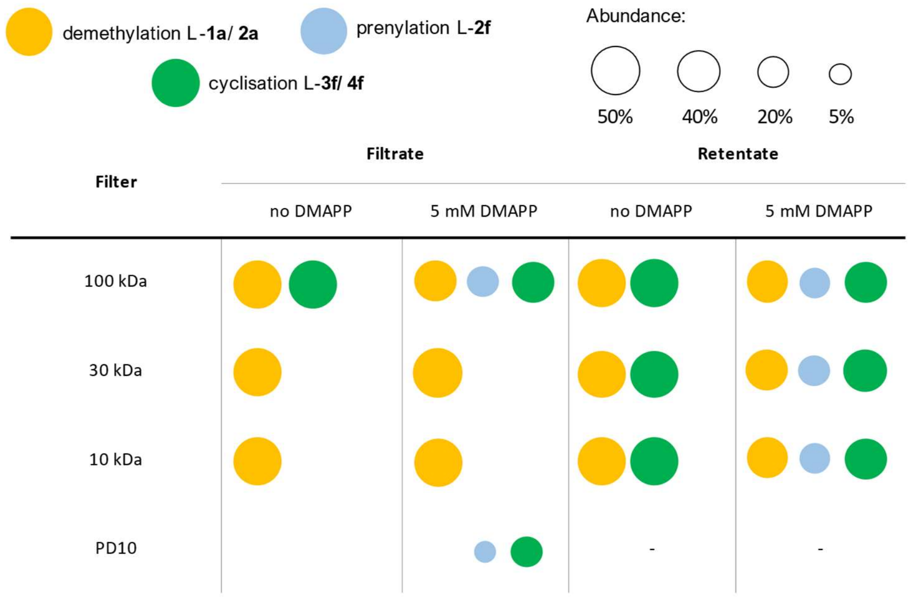

Figure S16: Overview of results obtained in "Investigation of different CFE preparation" indicating demethylation, prenylation and cyclization products with different colored circles and their abundance with different sizes. In case of reaction without DMAPP demethylation product is L-**1a** and with DMAPP L-**2a**, in case of cyclization without DMAPP the respective products are L-**3f** and with DMAPP L-**4f** as shown in the main article (Main Article, Scheme 2). Reaction conditions: L-**1f** (5 mM), DMAPP (5 mM), DmaW preparation [CFE of *E. coli* BL21(DE3)] (20 mg_{CFE}/mL, corresponding to 13 µM pure DmaW), Tris/HCl buffer (50 mM pH 7.5, 5 mM $CaCl_2$), 24 h, 30 °C under ambient atmosphere.

Demethylation of L-**1f** as well as the prenylation product L-**2f** was obtained in all reaction setups with filtrates from 100 kDa to 10 kDa centrifugal filtration. However, also in the retentate, which reflects a higher molecular weight fraction, the demethylation was detected. Only after filtering the CFE solution through PD10 columns, no demethylation of substrate or product was found. According to these results, the demethylation activity can be assigned to small chemical species that are present in the CFE of *E. coli* BL21(DE3).

On the other hand, cyclization of substrate and product can only be found in the filtrate after centrifugation with 100 kDa centrifugal filters. In all the other centrifugal filtrates no cyclized form can be obtained, whereas all retentates give cyclization. Also, the PD10 eluate, which contains only high-molecular mass components of the CFE, gave cyclization. Summarizing these results,

the cyclization towards L-**3f** and L-**4f** observed as side reaction in the DmaW-catalyzed prenylation of L-**1f** is most likely due to other enzymes inside of the *E. coli* CFE. In contrast to small chemical species, enzymes with a molecular weight of 30 kDa and higher cannot pass the centrifugal filters of 10 and 30 kDa, but can flow through the PD10 column, whereas small molecules and ions are retained in the PD10 column.

HR-MS results of products in prenylation of L-1f

For additional characterization of the obtained products in prenylation of L-**1f** by DmaW preparation (CFE of *E. coli* BL21(DE3)), the isolated compounds including the apparent cyclic oxidation product L-**4f** (*m/z* = 285), L-**2a** (*m/z* = 273), L-**2f** (*m/z* = 287) as well as L-**1f** (*m/z* = 219) and L-**1a** (*m/z* = 205), from upscaled biotransformations (15 mM, 100 mg) were subjected to HR-MS analysis. The results of these measurements are shown in Table S6.

Table S6: HR-MS results for isolated compounds L-**2a**, L-**2f**, L-**3f**, L-**1f**, L-**1a** indicating the chemical structure, the experimental mass, and the calculated mass.

Substance	*m/z* (experimental)	*m/z* (calculated)
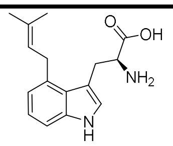 L-**2a**	273.16023	273.159754
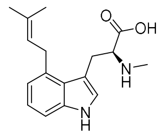 L-**2f**	287.175352	287.175404
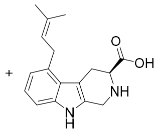 L-**3f**	285.159563	285.159754

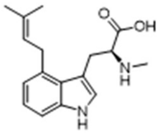 L-**1f**	219.112976	219.112804
L-**1a**	205.097567	205.097154

OrbiTrap measurement of L-1f prenylation

To get more structural information on the obtained peaks in prenylation of L-**1f** by DmaW, OrbiTrap measurements of the HR-MS samples were conducted. All obtained chromatograms and MS/MS spectra are depicted in "OrbiTrap chromatograms and spectra".

In general, high tendencies towards water adduct formation were evident in the obtained chromatograms, leading to peaks with coinciding retention times and an *m*/*z* difference of 18 (*e.g.*, *m*/*z* 273.16058 and 291.17056 for **2a**). In the corresponding MS/MS spectra, the water adducts have low intensities, and hence the spectra of the water adducts and the parent ions are practically identical.

L-**2a** and L-**2f** possess an intensive fragment at 256.133, which corresponds to a cleavage of the amino group (methyl-amino group in L-**2f**), a fragmentation that has recently been documented for several open-chain amino acids, including tryptophan.[4] Potentially, a shift in charge or a cyclization to stabilize the cation may also occur, leading to isomeric structures of the same exact mass (Figure S17). Differentiation between these structures is not possible based on the obtained data.

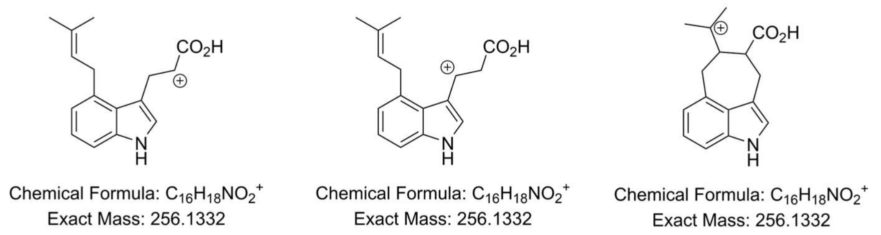

Figure S17: Cleavage of amino group from L-**2a** leads to three possible structures possessing the exact mass of 256.1332.

Moreover, the masses 217.097 and 231.113 in L-**2a** and L-**2f**, respectively, indicate a cleavage of the prenyl group forming a benzylic cation, keeping the amino acid part intact (Figure S18).

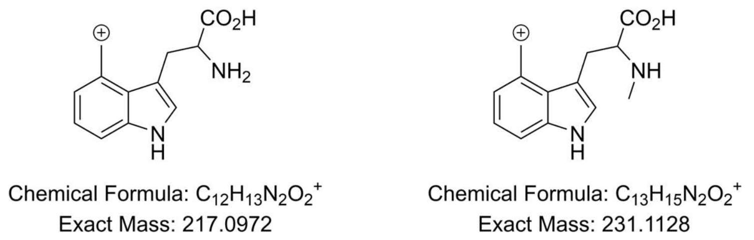

Figure S18: Benzylic cations of L-**1a** and L-**1f** after cleavage of prenyl group.

In case of L-**2f** a fragment with *m*/*z* 88.039 was visible, which corresponds to an *N*-methylglycinium cation. An analogous fragment with a mass of 74.024 has been reported in the literature for tryptophan (Figure S19).[4]

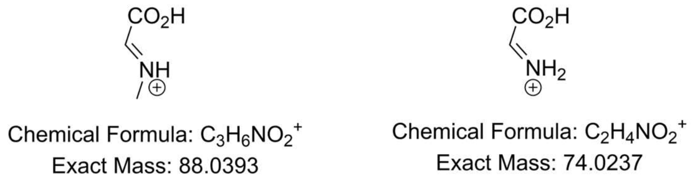

Figure S19: *N*-methylglycinium cation and glycinium cation.

For reaction mixture samples containing all obtained products as depicted in Figure S14, a product peak at 15.10 min (*m*/*z* 303.170, water adduct of cyclization product **4f**) overlapped with other ions, namely L-**2f** (305.186) and an unidentified, doubly oxidized product (301.154). Easier to interpret were the fragments of the molecular ion ($M+H^+$, 285.159), with *m*/*z* 268.132 and 254.117. Their weak intensity suggests cleavage of the amino-acid nitrogen to be unfavorable,

pointing towards a cyclic structure. The largest significant fragment 229.097 corresponds to the benzylic cation and supports the loss of two units of mass compared to L-**2f** in the right-hand area of the molecule (amino acid moiety) and not in the prenyl group (Figure S20).

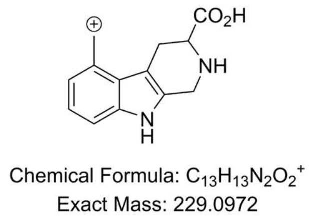

Figure S20: Benzylic cation indicating the loss of two mass units in the right-hand area of the molecule.

The intense fragment 212.143, with a loss of 73 mass units compared to the molecular ion (285.159), would be unusual for open-chain, *N*-methylated compounds, whereas for the cyclic, non-prenylated amino-acid 1,2,3,4-tetrahydro-β-carboline-3-carboxylic acid a cleavage like this is known, with a fragment of 144.081 giving the most intense ion (Figure S21).[5]

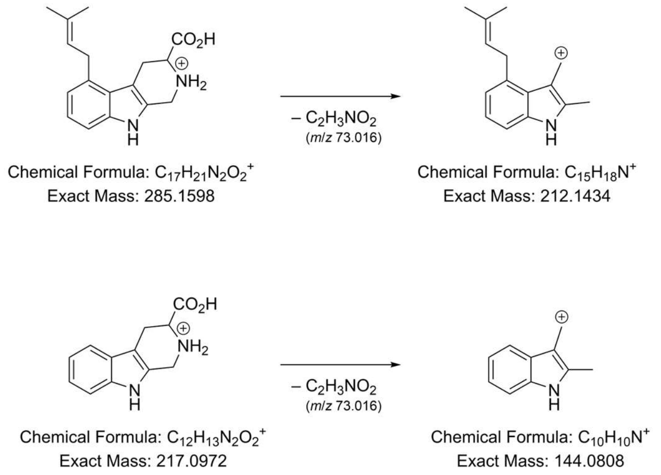

Figure S21: Hypothesized fragmentation of cyclic, prenylated amino acid **4f** (top) and an analogous, literature-known cleavage of cyclic, non-prenylated amino acid **3f** (bottom).

Further compounds identified in the reaction mixture samples from L-**1f** prenylation are **1a** (11.1 min, 205.097), **1f** (11.5 min, 219.113), the cyclization product of abrine (**3f**; 12.8 min, 217.097) and a compound that appears to be a methylated derivative of **3f** (13.5 min, 231.113).

Table S7: Overview of obtained products in prenylation of L-**1b** by DmaW preparation [CFE of *E. coli* BL21(DE3)].

Product Structure	Product Abbreviation	Source
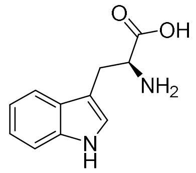 *m/z* 205	L-**1a**	Demethylation by BL21 CFE
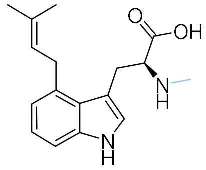 *m/z* 287	L-**2f**	Prenylation by DMATS
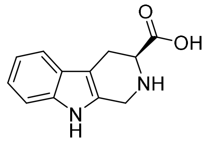 *m/z* 217	L-**3f**	Demethylation and oxidative cyclization by BL21 CFE
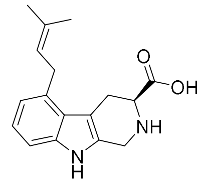 *m/z* 285	L-**4f**	Prenylation by DMATS and oxidative cyclization by BL21 CFE

Activity measurements for 1h and 1i using DmaW from *A. japonicus*

To examine a potentially weaker binding of indole derivatives **1h** and **1i** in the active site of DmaW, activity measurements were performed at concentrations of 0.5–25 mM of tryptophan derivative and 5 mM DMAPP.

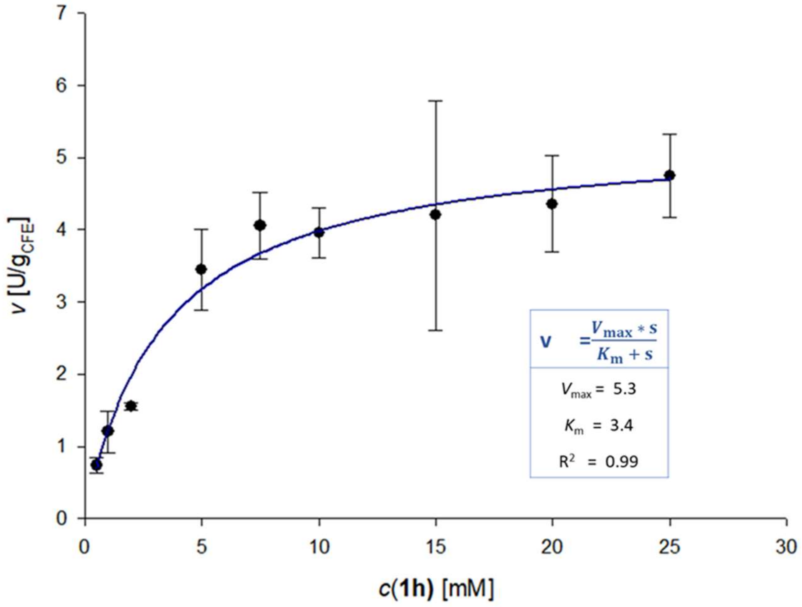

Figure S22: Rate determined by consumption of substrate vs. **1h** concentration. Reaction conditions: **1h** (0.5–25 mM), DMAPP (5 mM), DmaW preparation [CFE of *E. coli* BL21(DE3)] (20 mg_{CFE}/mL, corresponding to 13 µM pure DmaW), Tris/HCl buffer (50 mM pH 7.5, 5 mM $CaCl_2$), DMSO (5% v/v), 30 °C, 40 min.

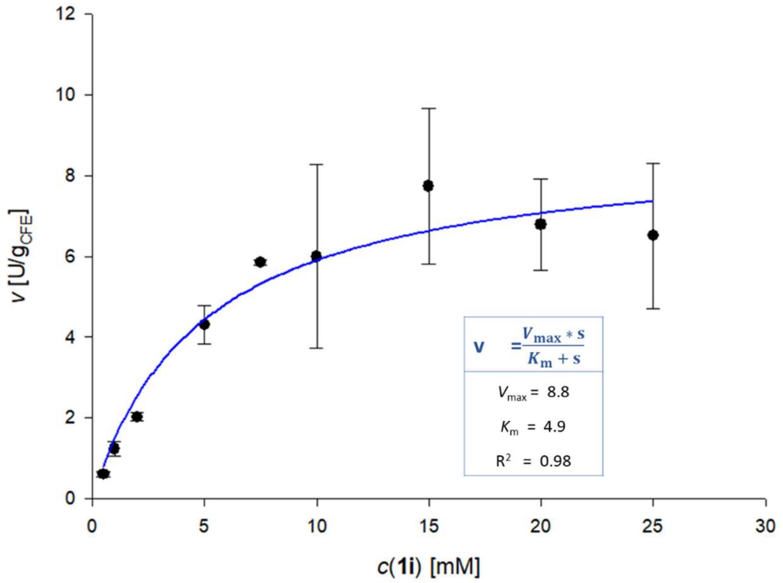

Figure S23: Rate determined by consumption of substrate vs. **1i** concentration. Reaction conditions: **1i** (0.5–25 mM), DMAPP (5 mM), DmaW preparation [CFE of *E. coli* BL21(DE3)] (20 mg_{CFE}/mL, corresponding to 13 µM pure DmaW), Tris/HCl buffer (50 mM, pH 7.5, 5 mM $CaCl_2$), DMSO (5 % v/v), 30 °C, 40 min.

Analysis: Chromatography columns, conditions, and retention times

HPLC analysis

Achiral reversed-phase HPLC was performed on an Agilent 1260 Infinity HPLC system (Santa Clara, CA, USA) consisting of a G1311B quaternary pump, a G1329B autosampler, a G1316A temperature-controlled column compartment and a G4212B diode array detector and for mass spectrometry a 6120 single-quadrupole LC/MS detector. A Phenomenex Luna® 5 µm C18(2) 100 Å 250 × 4.5 mm column and an Astec Chirobiotic T 5 µm, 25 cm × 4.6 mm column were used. The typical injection volume was 10 µL, chromatograms were monitored at 262 nm and an internal standard 1-propiophenone was used to account for the variation in UV response. All solvent mixtures are given in (v/v) ratios.

Method *achiral* for HPLC-UV and HPLC-MS

Column:	Luna C18 (2)
Column temperature:	30 °C
Eluent gradient:	H_2O/MeCN = 95:5 (+ 0.1% TFA for UV, + 0.1% FA for MS), 2 min;<br>linear gradient to H_2O/MeCN = 30:70 (+ 0.1% TFA for UV, + 0.1% FA for MS), 13 min;<br>linear gradient to 100% MeCN (+ 0.1% TFA for UV, + 0.1% FA for MS), 1 min;<br>100 % MeCN (+ 0.1% TFA for UV, + 0.1% FA for MS), 4 min;<br>100 % H_2O (+ 0.1% TFA for UV, +0.1% FA for MS), 5 min;
Elution flow:	1.0 mL/min
Run time:	25 min
Integration wavelength:	262 nm

Method *chiral* for HPLC-MS

Column:	Astec Chirobiotic T
Column temperature:	30 °C
Eluent gradient:	isocratic mode with H_2O/MeOH = 30:70 (+ 0.1% FA)
Elution flow:	1.0 mL/min
Run time:	12 min

Table S8: Retention times of investigated substrates and respective products in HPLC-UV analysis

Substrate	Product	Substrate retention time [min]	Product retention time [min]
L-**1a**	**2a**	10.1	13.5
D-**1a**	**2a**	10.1	13.5
L-**1b**	**2b**	11.0	14.5
L-**1c**	**2c**	8.4	11.4
rac-**1d**	**2d**	10.1	13.1
rac-**1e**	**2e**	11.7	15.1
L-**1f**	**2f**	10.4	13.7
L-**1g**	**2g**	11.1	14.6
**1h**	**2h**	14.1	17.9
**1i**	**2i**	11.3	13.5

HR-MS measurements

HR-MS measurements were performed by HPLC-TOF-MS on an Agilent 1260 Infinity Series HPLC coupled to an Agilent 6230 TOF LC/MS detector operated in positive ESI mode. 0.05 µL sample were injected and separated with isocratic method 40% HPLC-grade H_2O and 60% MeCN/H_2O (5:1 + 0.1% 5 M ammonium formate) and a flow rate of 0.3 mL/min.

OrbiTrap measurement

OrbiTrap measurements were performed using a Dionex Ultimate 3000 HPLC system coupled to a Q-Exactive Orbitrap Mass Spectrometer (Thermo Fisher Science, Erlangen, Germany) equipped with a heated electrospray ionisation (HESI) source. The chromatography was performed using the same column and eluting conditions as described above for the "Method achiral for HPLC-UV" system; the injection volume was 5 µL. Mass spectrometry was performed in the positive ion mode and source settings were: spray voltage 3500 V, capillary temp 300 °C, gas temp 350 °C, with flow rates of 65 (sheath) and 20 (aux) instrument units. The full scan range was 50–450 *m*/*z* (mass/charge) with the resolution set to 70,000 (full width half-maximum, FWHM), an automatic gain control (AGC) target of 1e6 and a maximum injection time (IT) of 100 ms were

used. Data dependent tandem mass spectrometry (ddMS/MS) was performed with normalized collision energies (NCE) of 20, 40, 60 (stepped) instrument units for the fragmentation experiments. MS/MS settings were: maximum injection time 50 ms, AGC 2e5, resolution 17,500 FWHM.

Preparative HPLC for isolation

For isolation of desired prenylated compounds, scaled-up reaction mixtures were separated by preparative HPLC using a Nexera Prep HPLC system from Shimadzu (Kyōto, Japan) consisting of a two LC-20AP pumps, a FR10A fraction collector, a SDP-M20A diode array detector and a CBM-20A communications bus module. A Phenomenex Luna® 5 µm C18(2) 100 Å 250 × 21.2 mm column was used. The injection volume was 2 mL. Chromatograms were monitored at 262, 254, 280 and 210 nm and fractions were manually collected.

Method preparative HPLC

Column:	Luna C18 (2)
Column temperature:	30 °C
Eluent gradient:	H_2O/MeCN = 95:5 + 0.1% TFA for isolation of **2i**), 2 min;
	linear gradient to H_2O/MeCN = 30:70, 13 min;
	linear gradient to 100% MeCN, 1 min;
	100 % MeCN, 5 min;
	100 % H_2O, 1 min;
Elution flow:	30.0 mL/min
Run time:	22 min

Preparative HPLC chromatograms

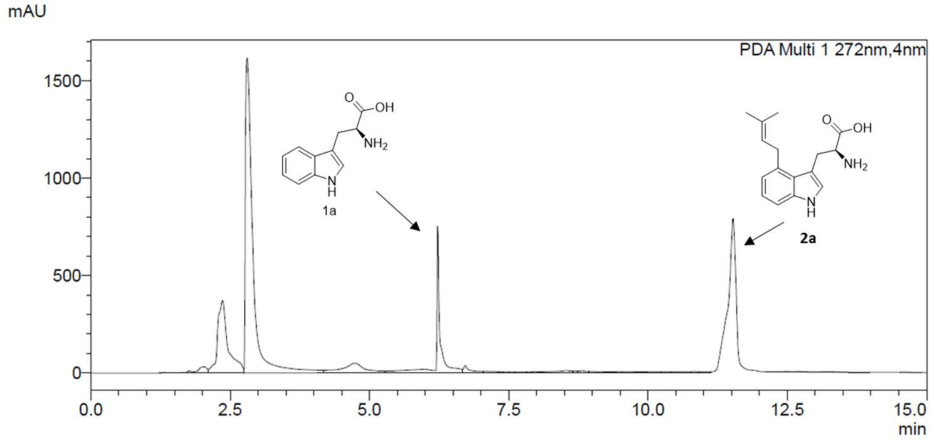

Figure S24: HPLC-UV chromatogram (272 nm) by preparative HPLC of L-**1a** prenylation by DmaW preparation [CFE of *E. coli* BL21(DE3)] showing substrate L-**1a** and product L-**2a**.

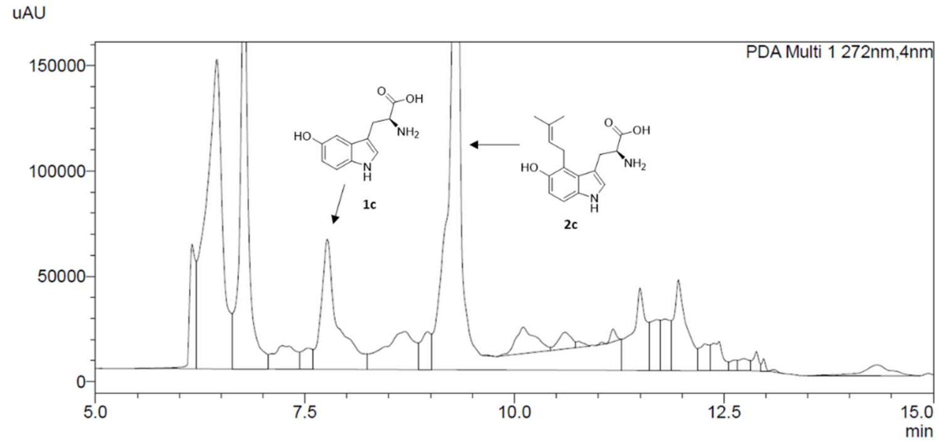

Figure S25: HPLC-UV chromatogram (272 nm) by preparative HPLC of L-**1c** prenylation by DmaW variant Y195S preparation [CFE of *E. coli* ArcticExpress (DE3)] showing substrate L-**1c** and product L-**2c**.

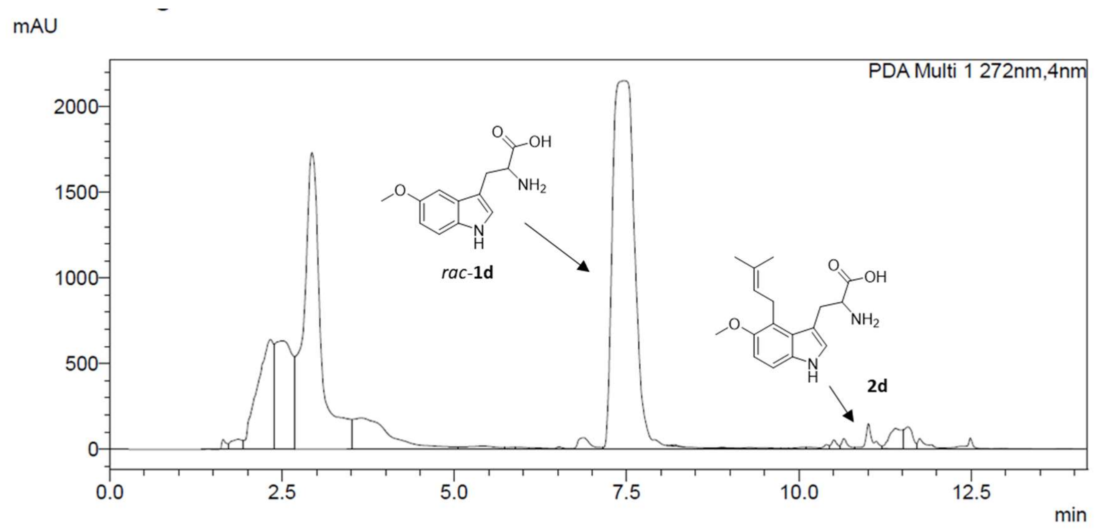

Figure S26: HPLC-UV chromatogram (272 nm) by preparative HPLC of *rac*-**1d** prenylation by DmaW variant Y195S preparation [CFE of *E. coli* ArcticExpress (DE3)] showing substrate L-**1d** and product **2d**.

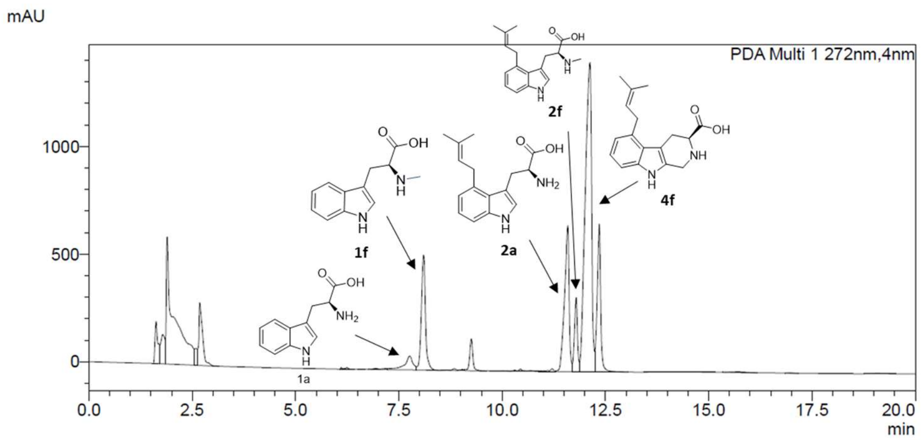

Figure S27: HPLC-UV chromatogram (272 nm) by preparative HPLC of L-**1f** prenylation by DmaW preparation [CFE of *E. coli* BL21(DE3)] showing L-**1a,** L-**1f**, product L-**2a,** L-**2f** and oxidation product L-**4f**.

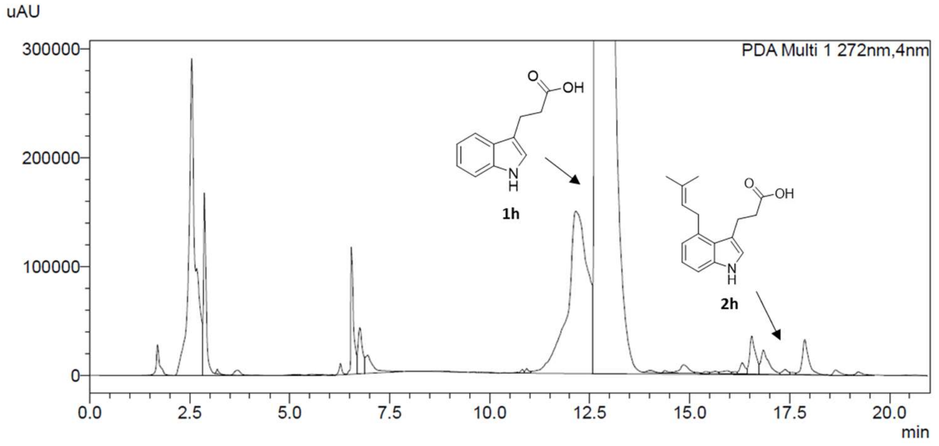

Figure S28: HPLC-UV chromatogram (272 nm) by preparative HPLC of L-**1h** prenylation by DmaW preparation [CFE of *E. coli* BL21(DE3)] showing substrate **1h** and product **2h**.

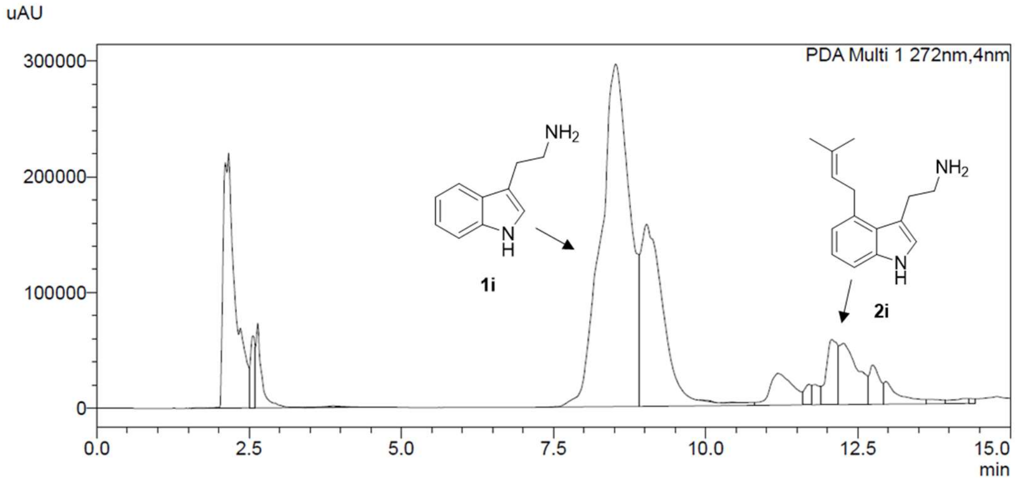

Figure S29: HPLC-UV chromatogram (272 nm) by preparative HPLC of **1i** prenylation by DMATS from *Claviceps purpurea* preparation [CFE of *E. coli* BL21(DE3)] showing substrate **1i** and product **2i**.

NMR spectra of synthesized compounds

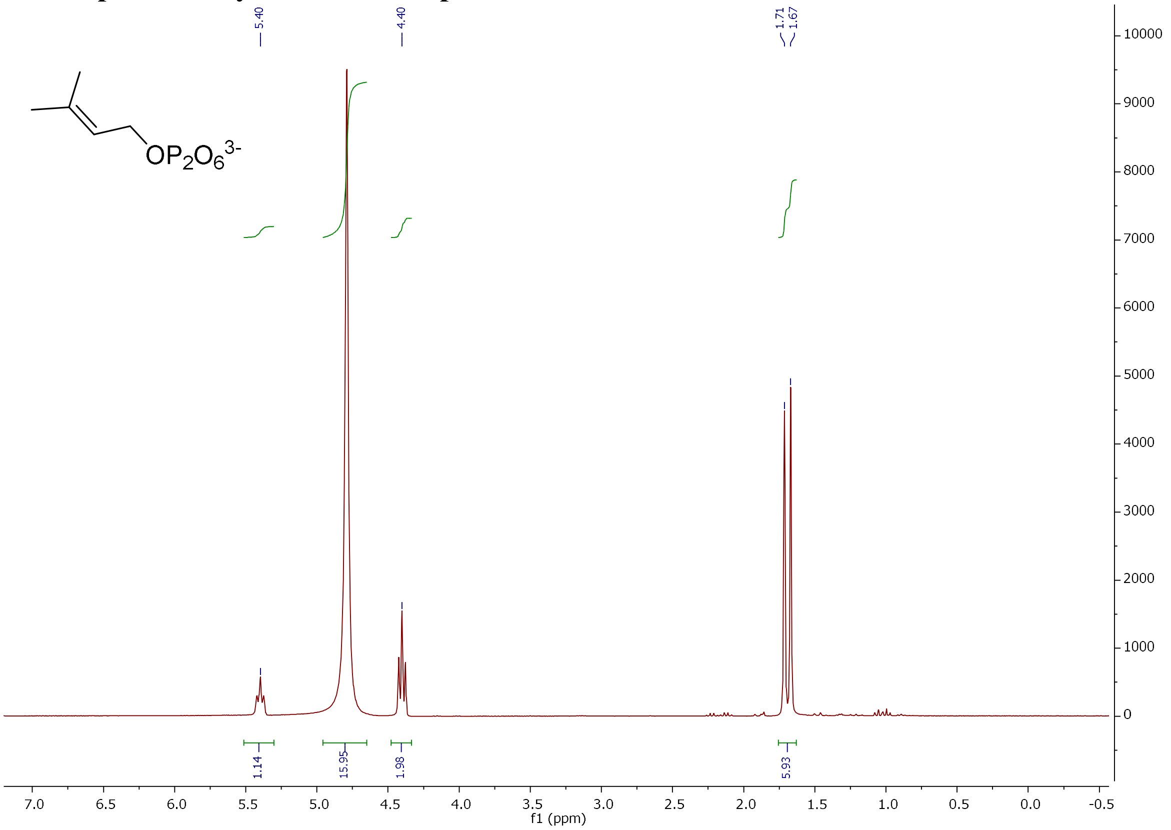

Figure S30: ^{1}H NMR spectrum of DMAPP in D_2O.

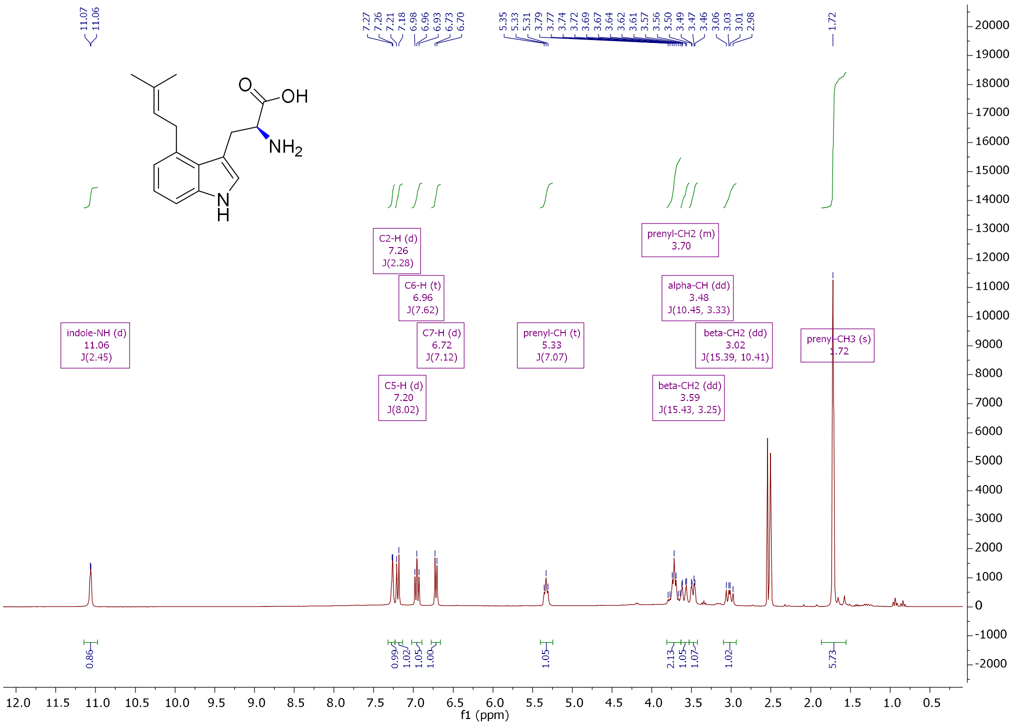

Figure S31: ^{1}H NMR (300 MHz) spectrum of L-**2a** in DMSO-d6.

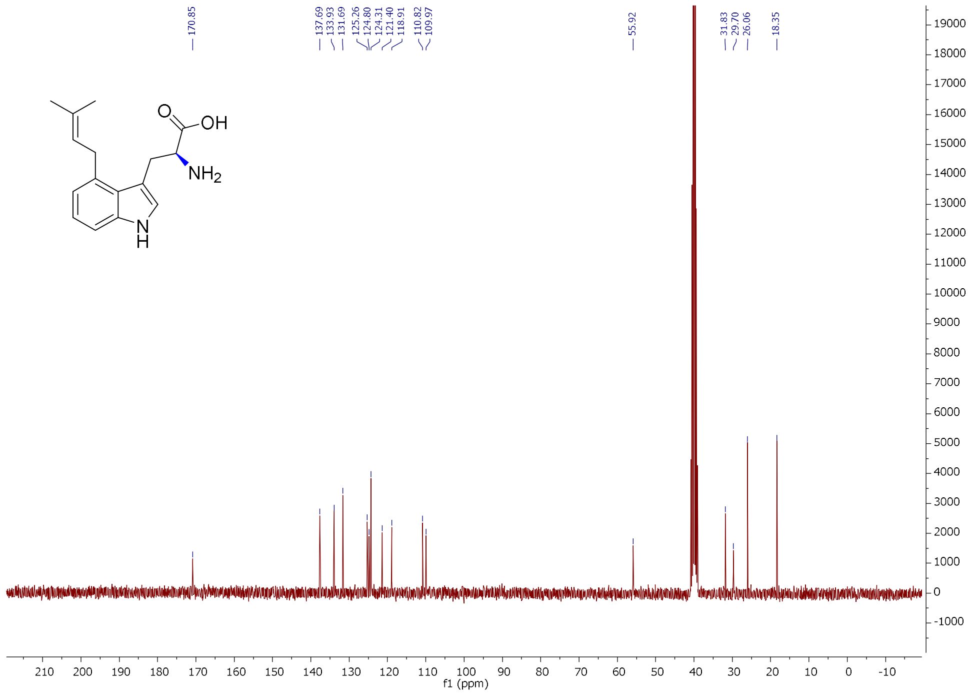

Figure S32: ^{13}C NMR (75 MHz) spectrum of L-**2a** in DMSO-d6.

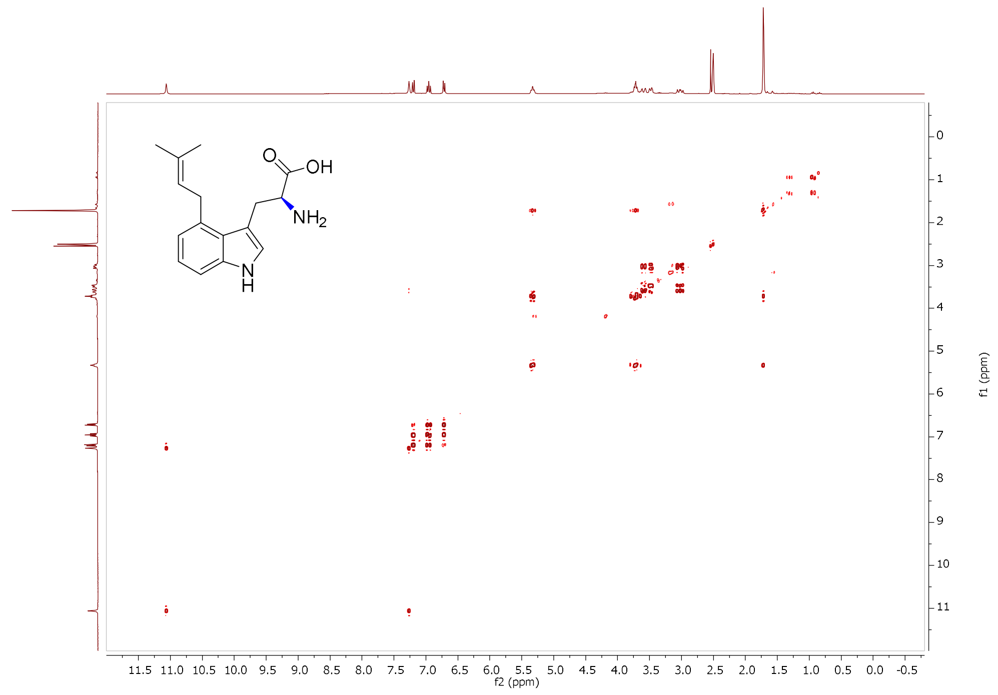

Figure S33: COSY NMR (300 MHz) spectrum of L-**2a** in DMSO-d6.

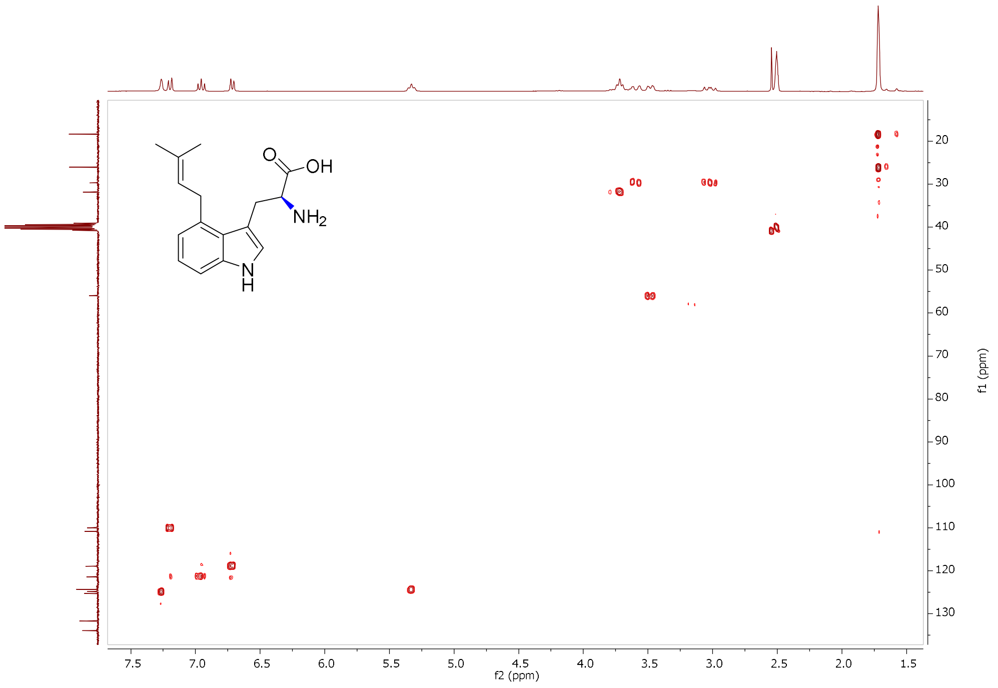

Figure S34: HSQC NMR (300 MHz) spectrum of L-**2a** in DMSO-d6.

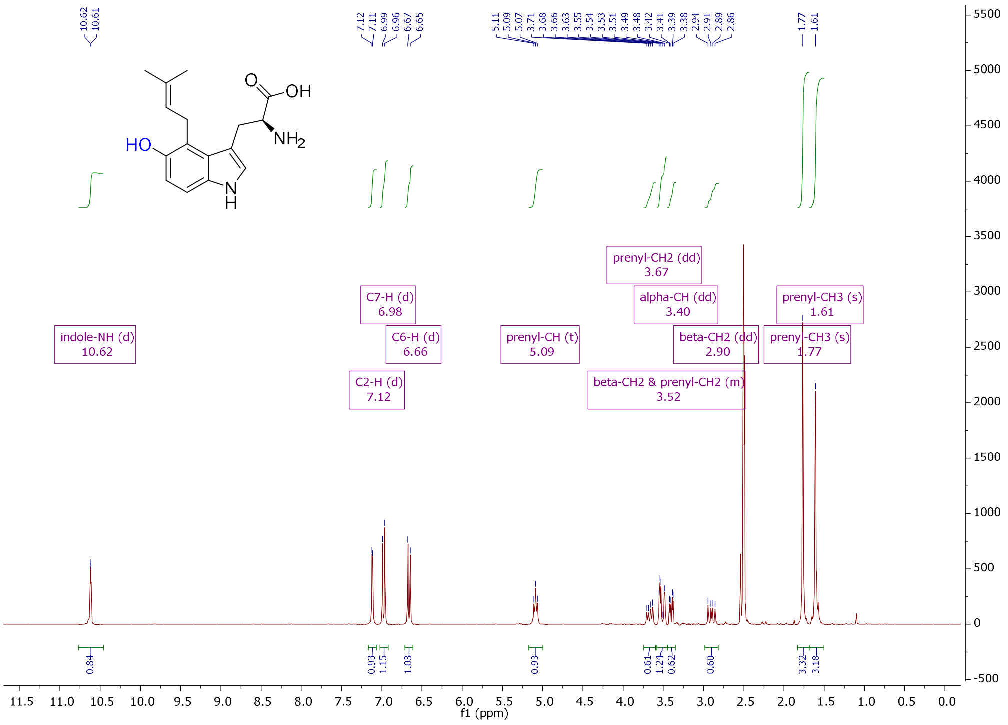

Figure S35: ^{1}H NMR (300 MHz) spectrum of L-**2c** in DMSO-d6.

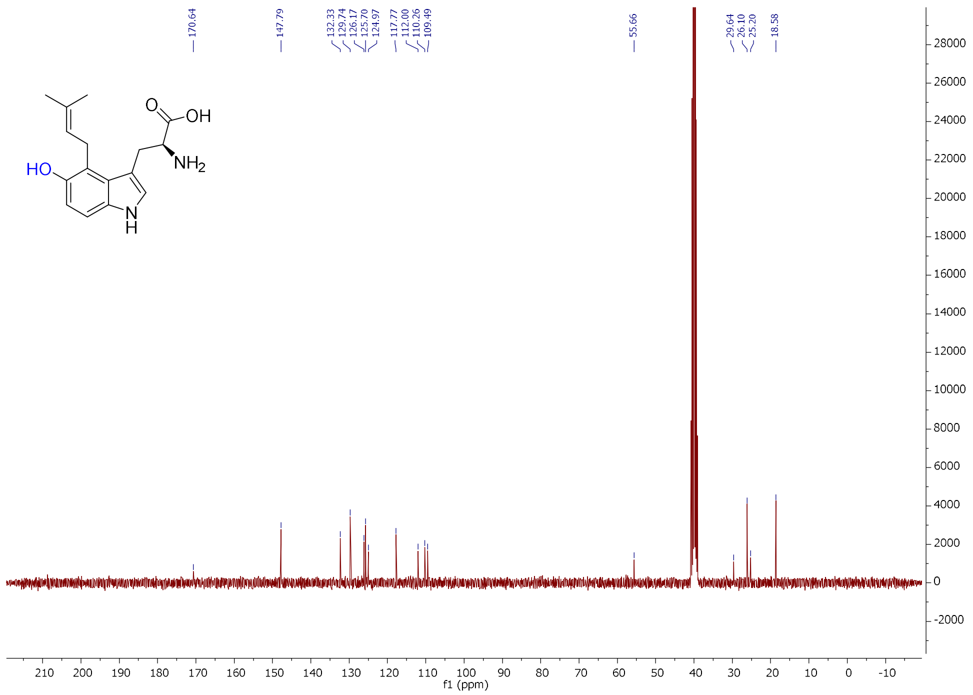

Figure S36: ^{13}C NMR (75 MHz) spectrum of L-**2c** in DMSO-d6.

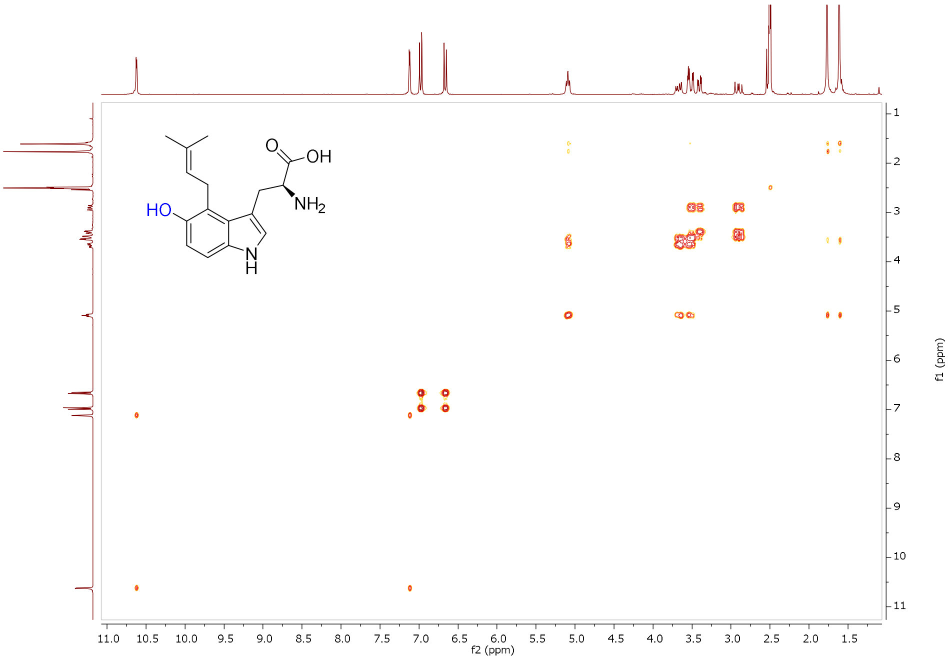

Figure S37: COSY NMR (300 MHz) spectrum of L-**2c** in DMSO-d6.

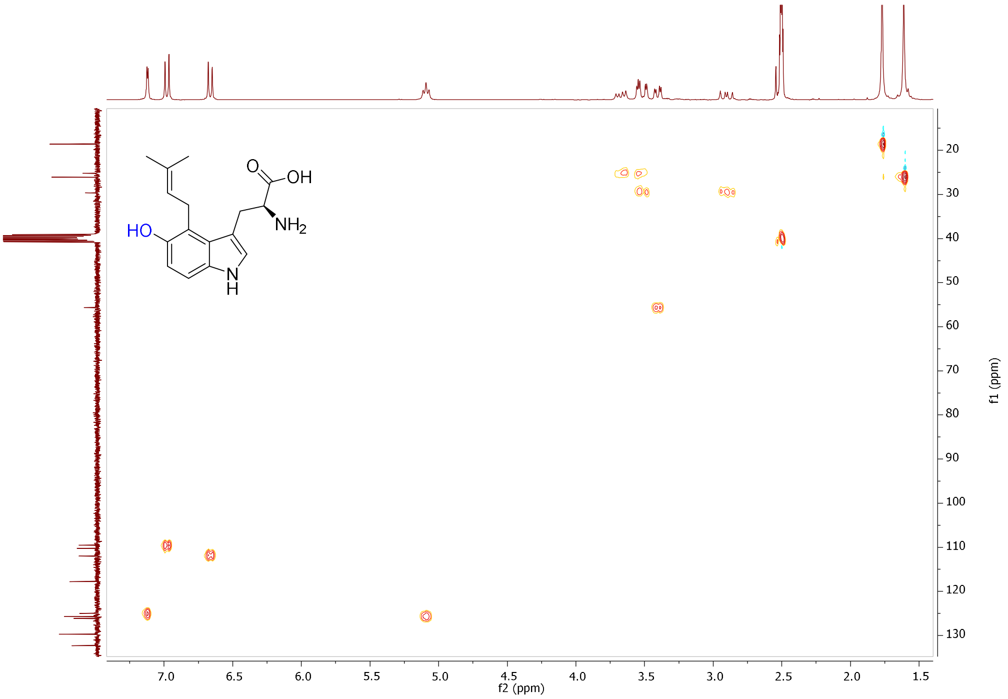

Figure S38: HSQC NMR (300 MHz) spectrum of L-**2c** in DMSO-d6.

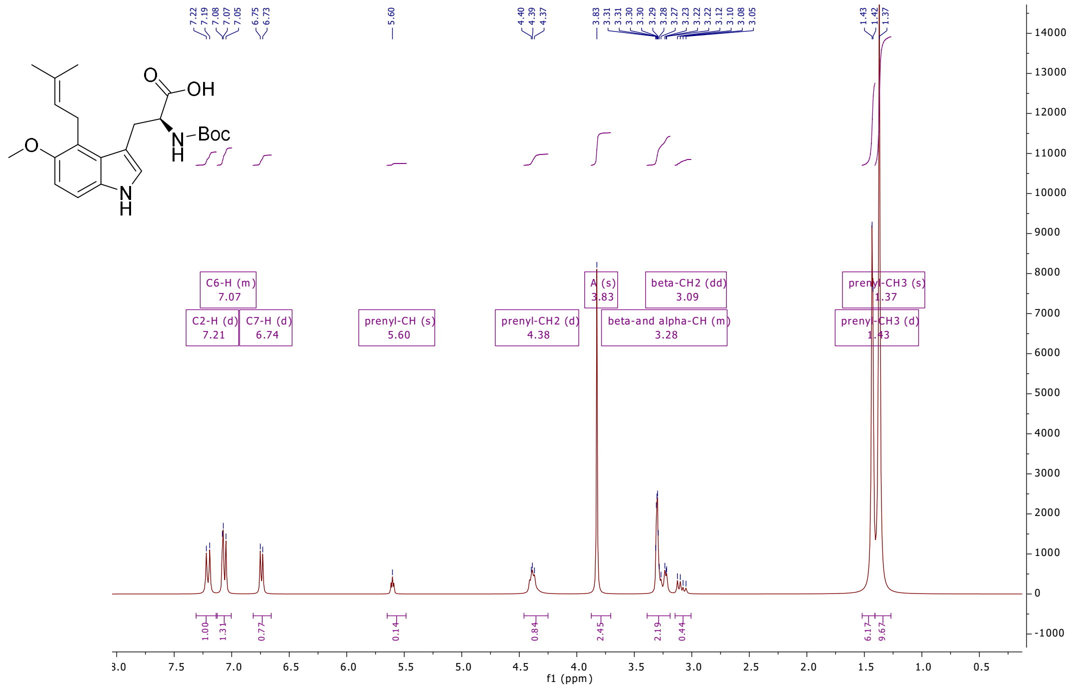

Figure S39: ^{1}H NMR (300 MHz) spectrum of isolated compound Boc-**2d** in methanol-d4.

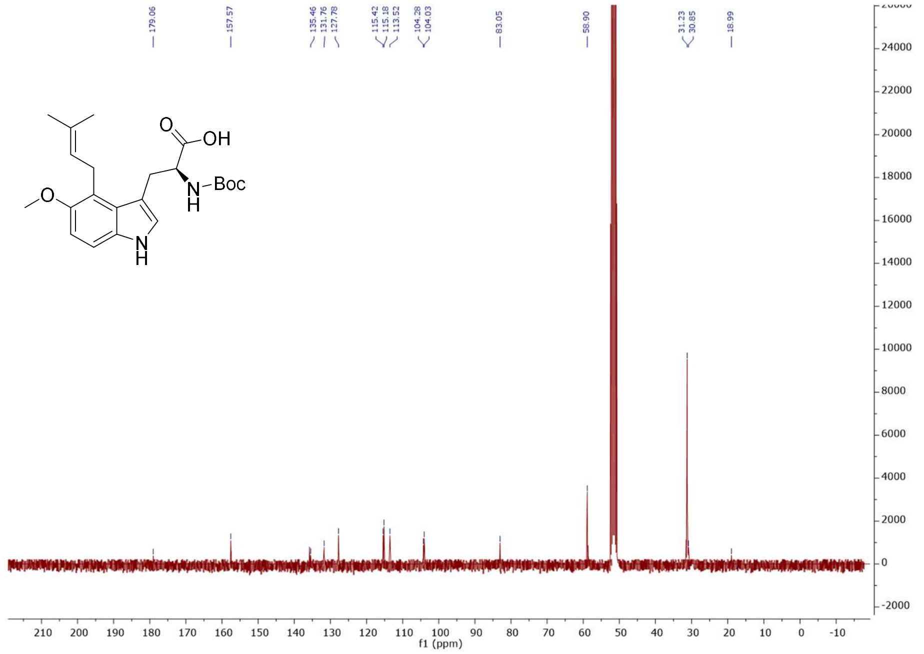

Figure S40: ^{13}C NMR (75 MHz) spectrum of Boc-**2d** in methanol-d4.

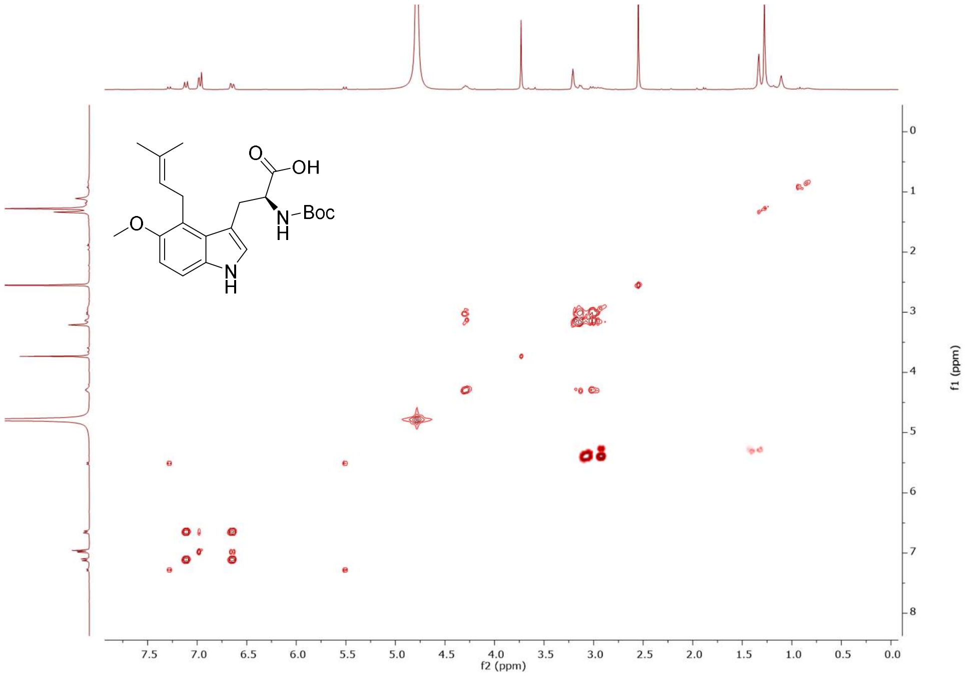

Figure S41: COSY NMR (300 MHz) spectrum of isolated compound Boc-**2d** in methanol-d4.

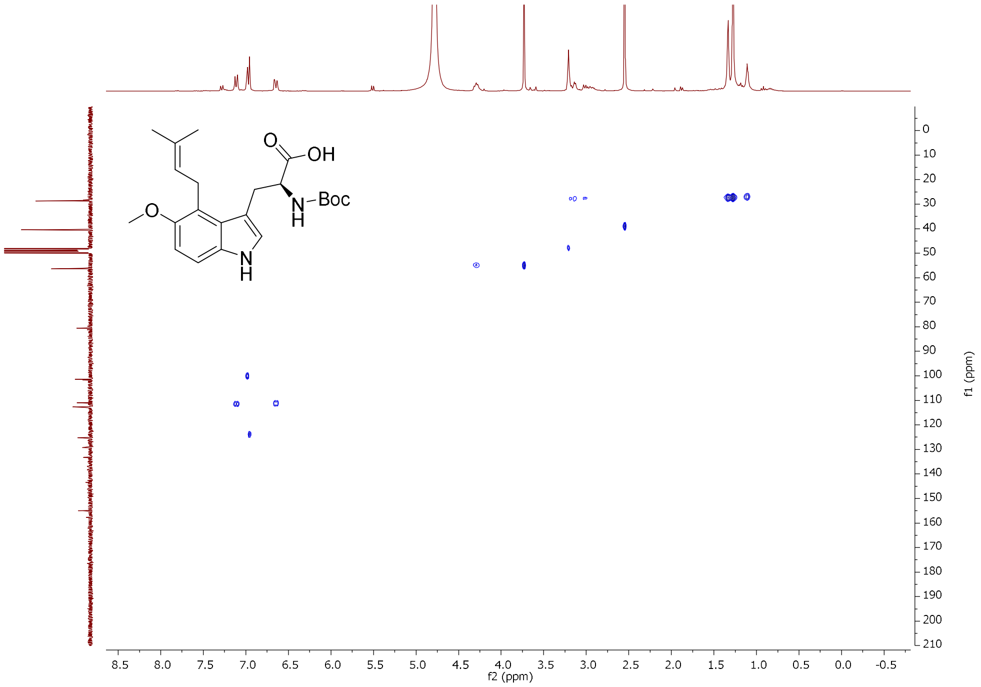

Figure S42: HSQC NMR (300 MHz) spectrum of isolated compound Boc-**2d** in methanol-d4.

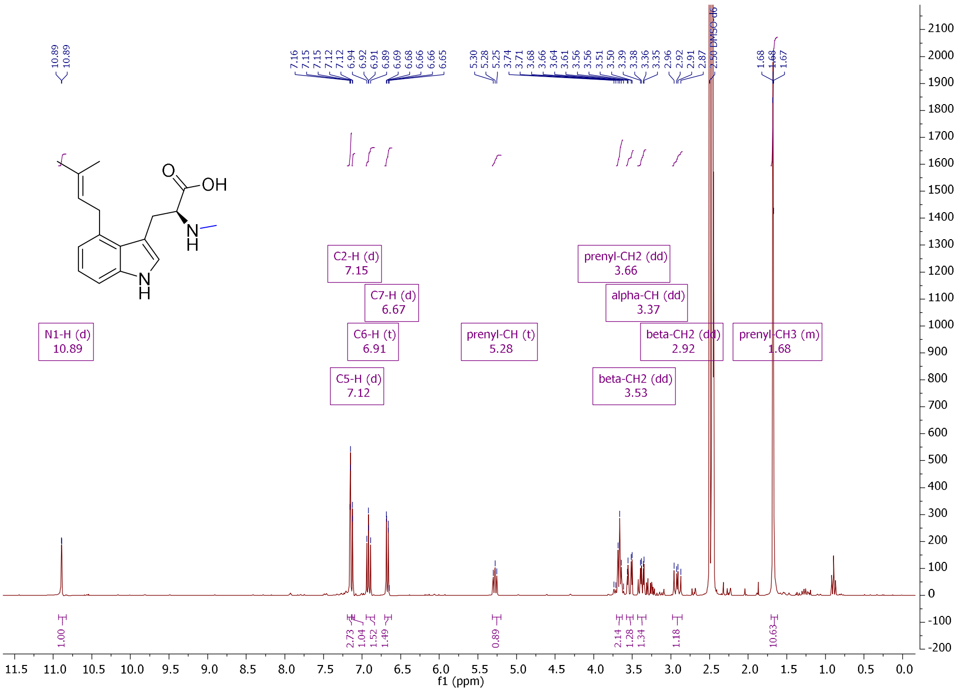

Figure S43: ^{1}H NMR (300 MHz) spectrum of L-**2f** in DMSO-d6.

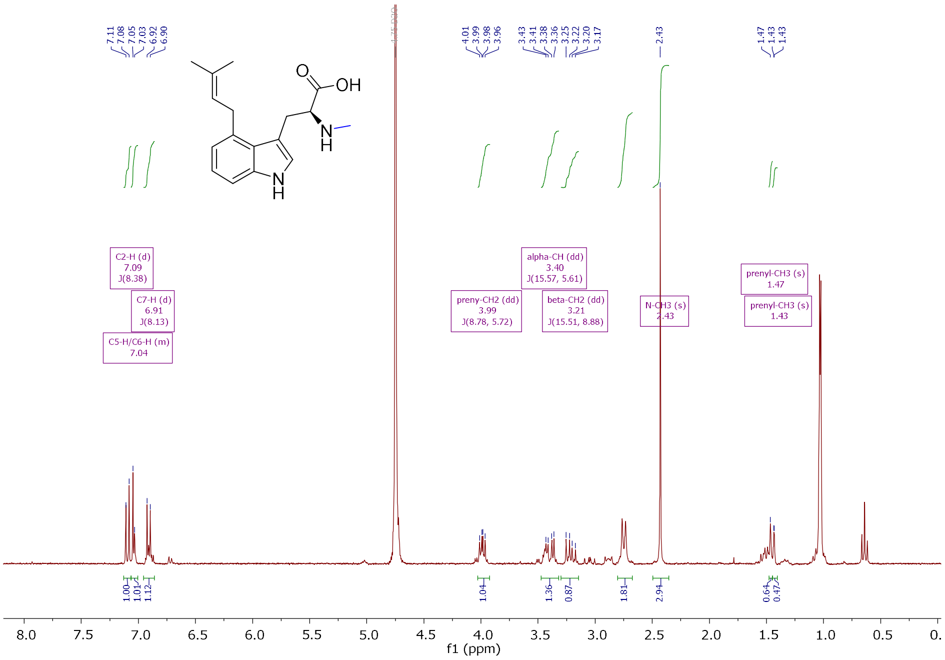

Figure S44: ^{1}H NMR (300 MHz) spectrum of L-**2f** in D_2O +2 drops DCl.

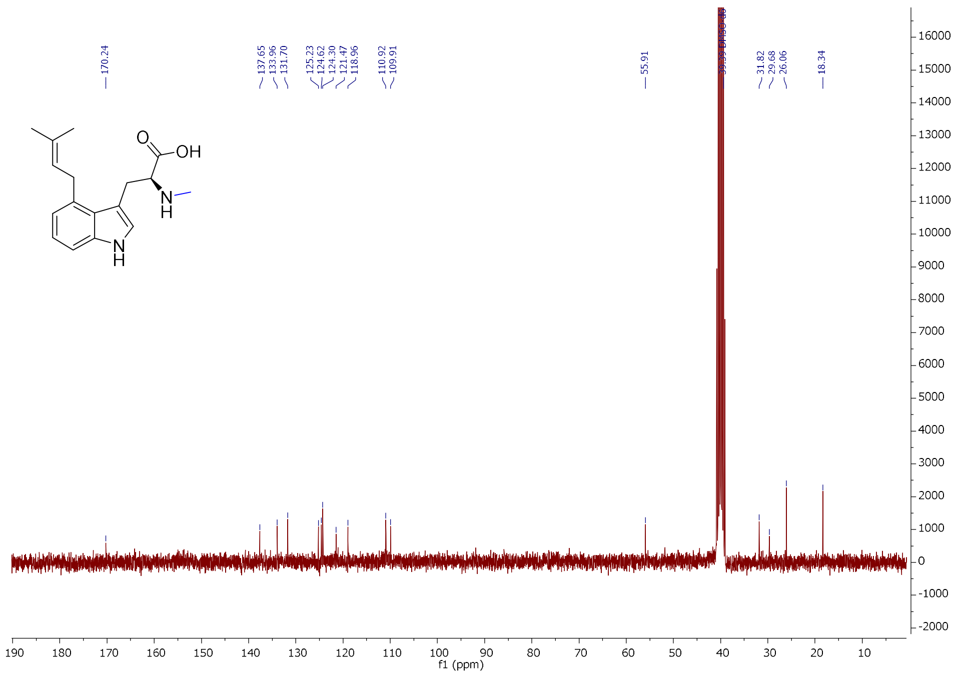

Figure S45: ^{13}C NMR (75 MHz) spectrum of L-**2f** in DMSO-d6.

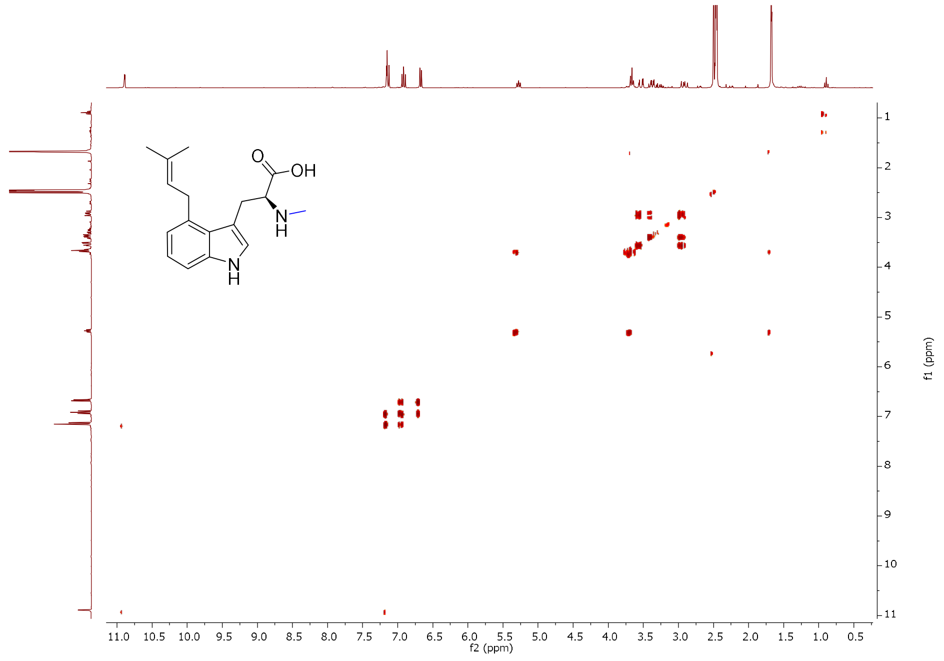

Figure S46: COSY NMR (300 MHz) spectrum of L-**2f** in DMSO-d6.

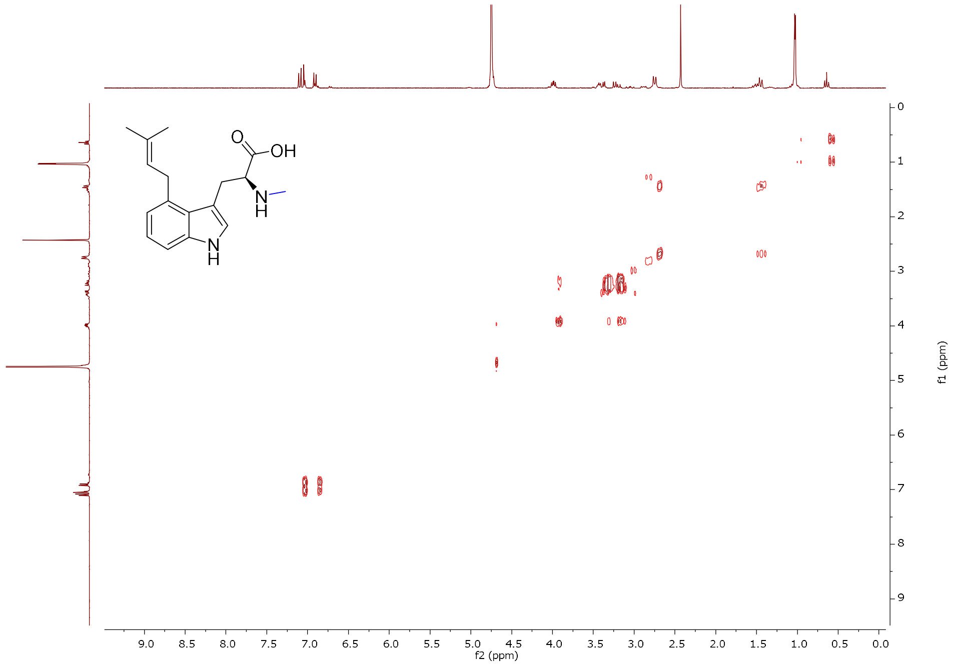

Figure S47: COSY NMR (300 MHz) spectrum of L-**2f** in D_2O +2 drops aqu. DCl.

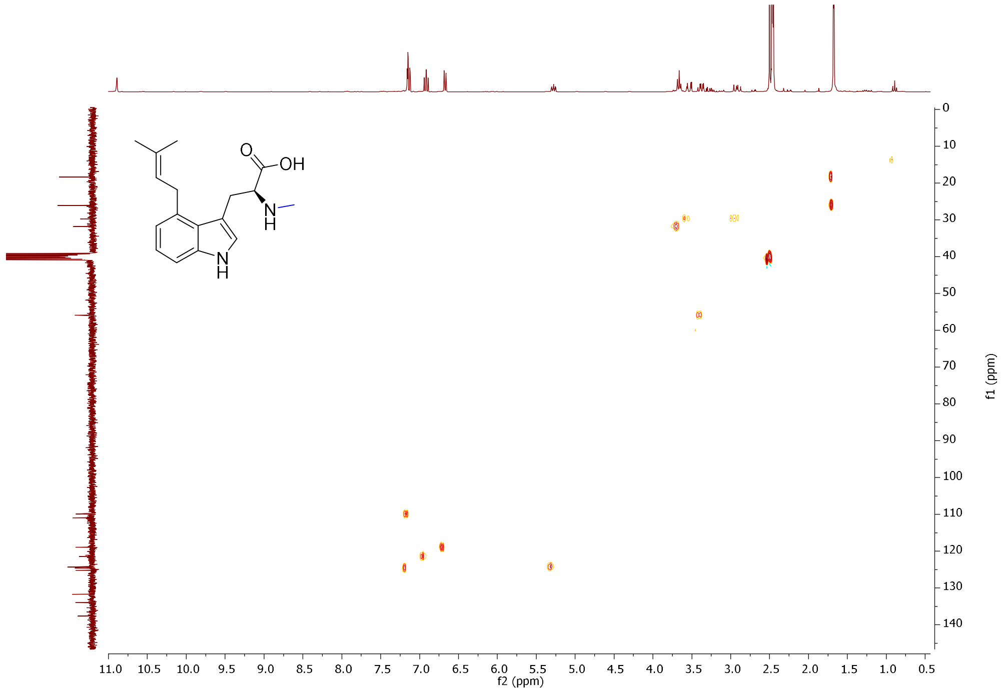

Figure S48: HSQC NMR (300 MHz) spectrum of L-**2f** in DMSO-d6.

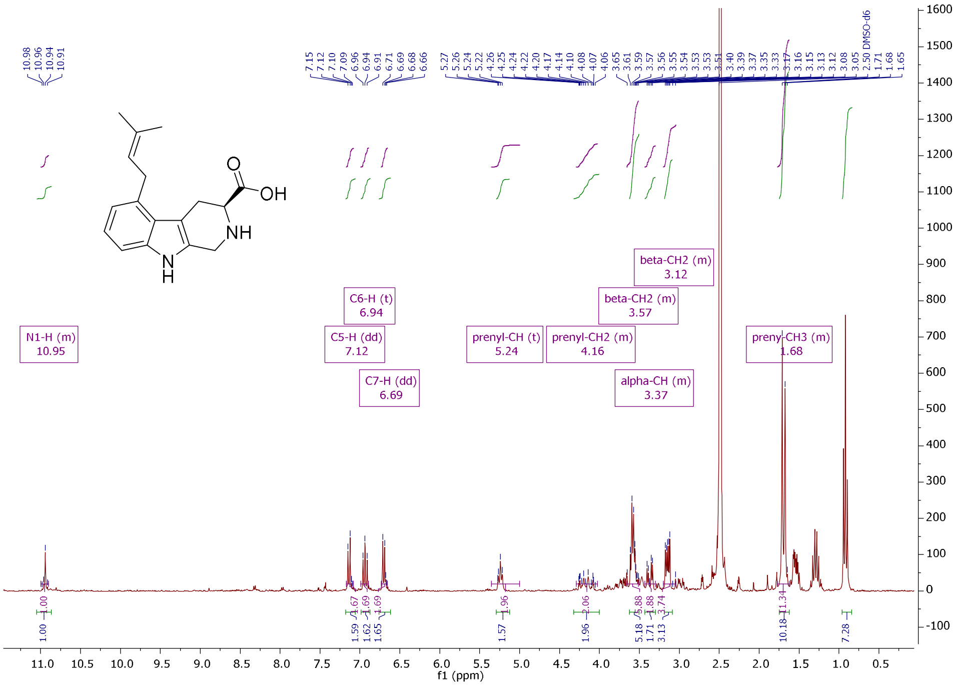

Figure S49: ^{1}H NMR (300 MHz) spectrum of L-**4f** in DMSO-d6.

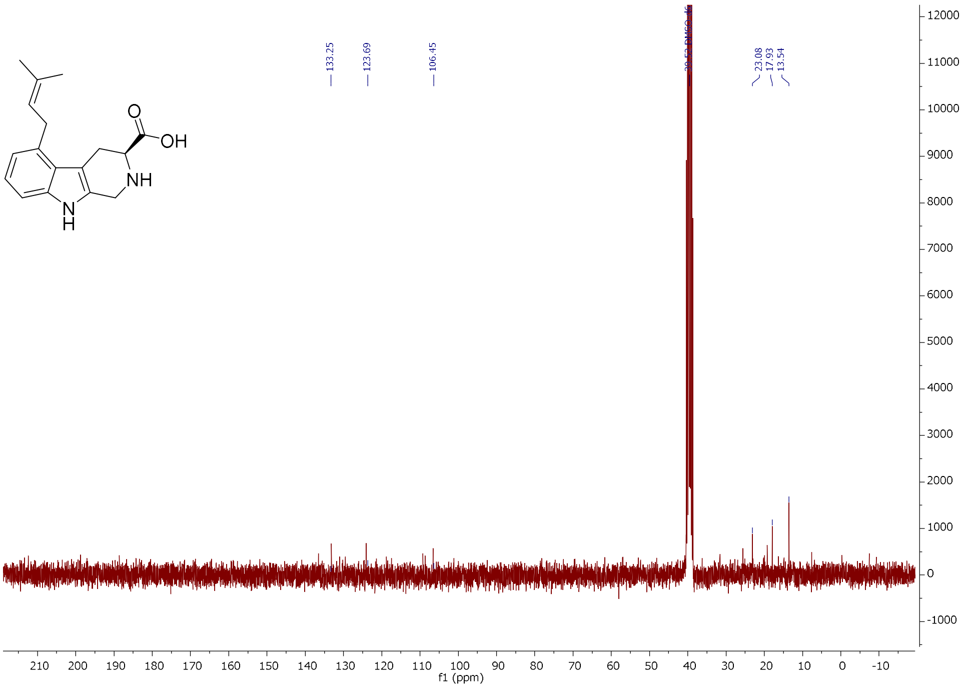

Figure S50: ^{13}C NMR (75 MHz) spectrum of L-**4f** in DMSO-d6.

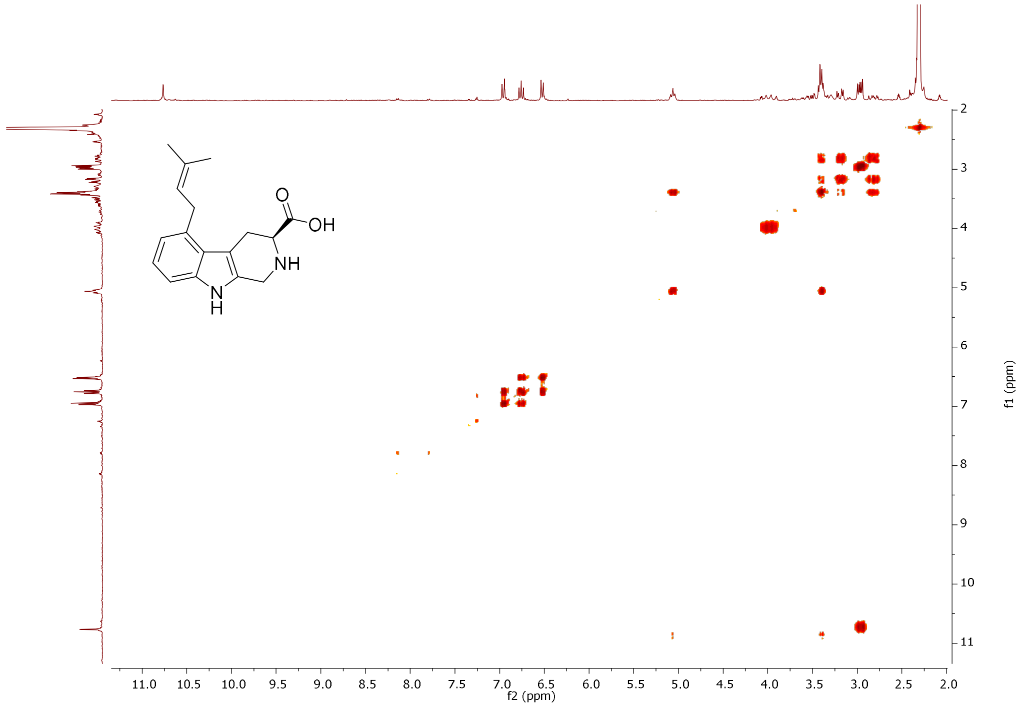

Figure S51: COSY NMR (300 MHz) spectrum of L-**4f** in DMSO-d6.

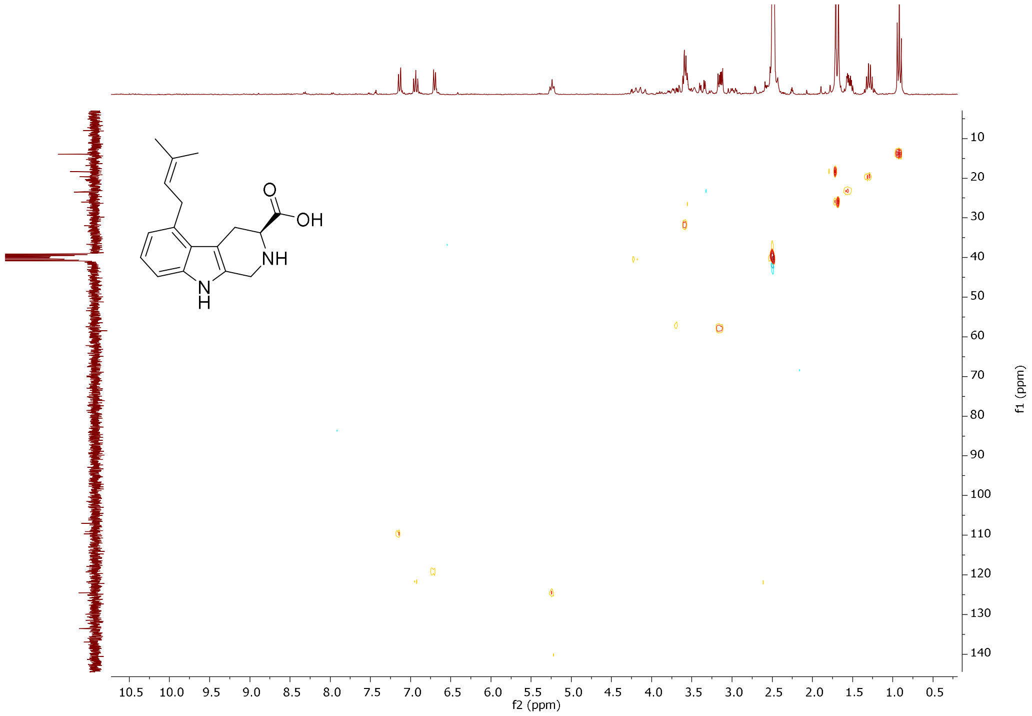

Figure S52: HSQC NMR (300 MHz) spectrum of L-**4f** in DMSO-d6.

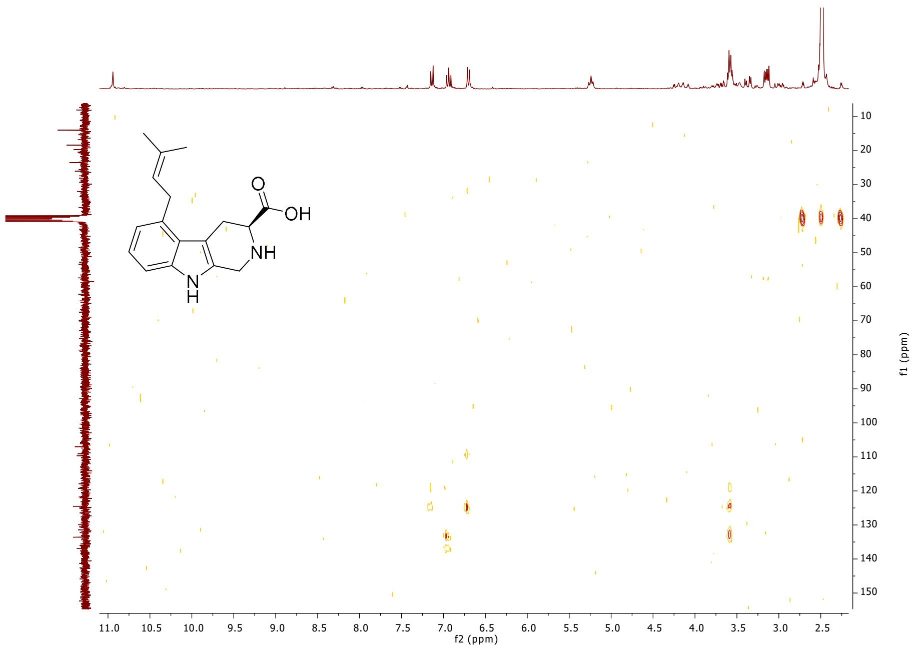

Figure S53: HMBC NMR (300 MHz) spectrum of L-**4f** in DMSO-d6.

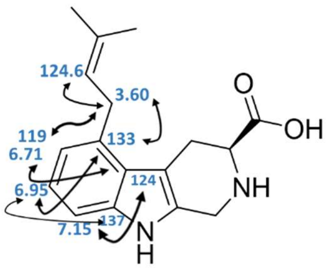

Figure S54: Correlations of signals in HMBC NMR spectrum of **L-4f** in DMSO-d6.

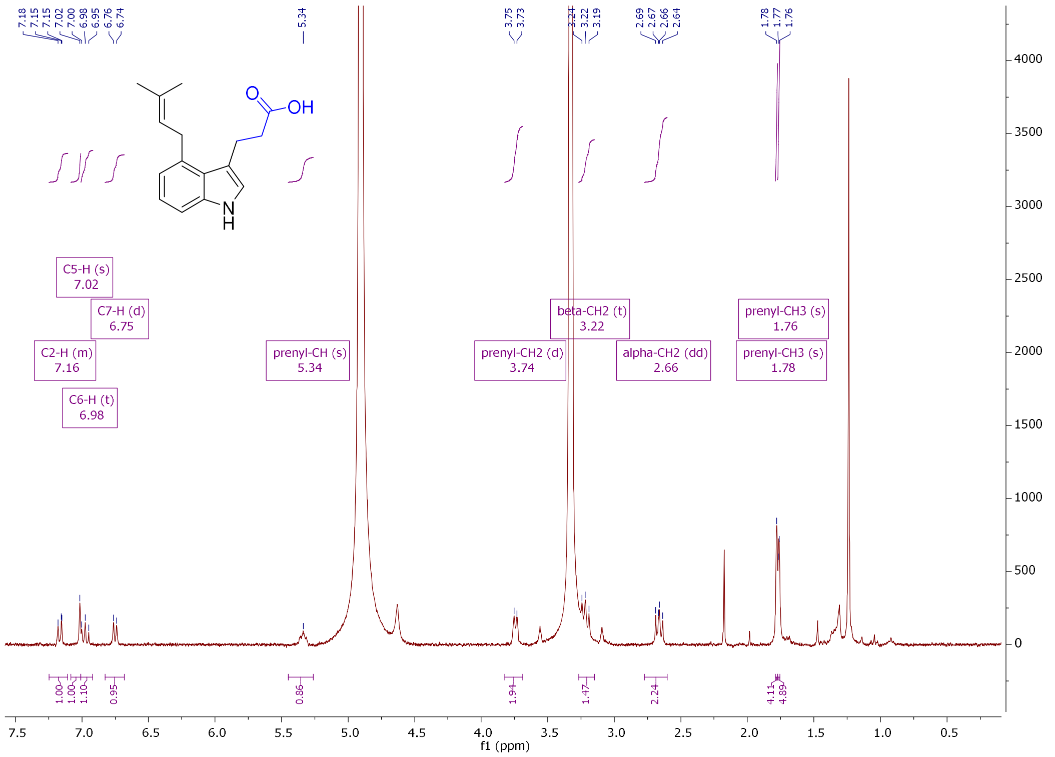

Figure S55: ^{1}H NMR (300 MHz) spectrum of isolated compound **2h** in methanol-d4.

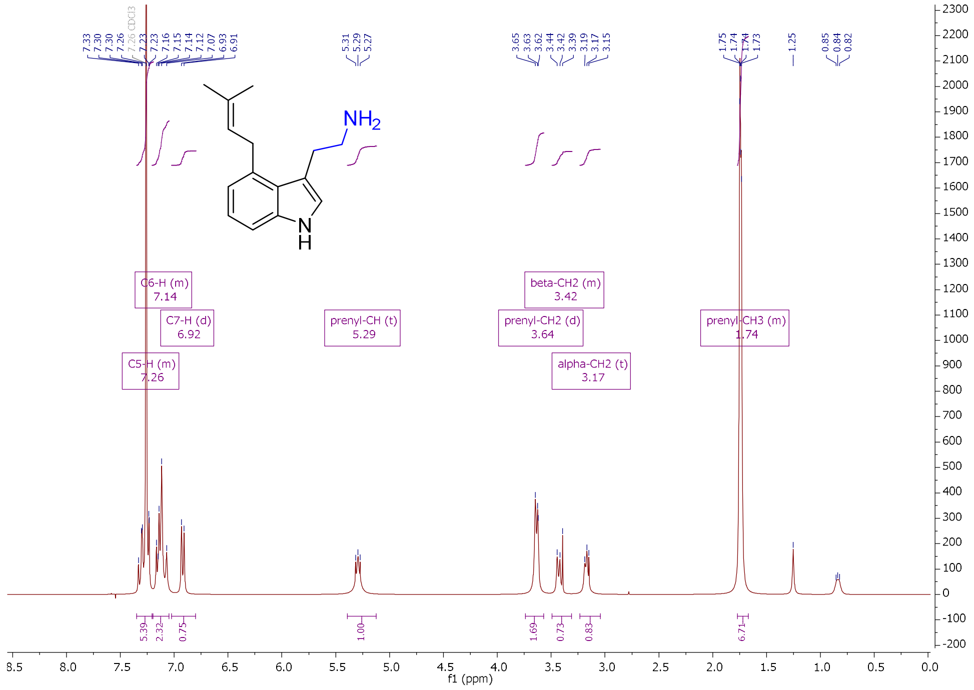

Figure S56: ^{1}H NMR (300 MHz) spectrum of isolated compound **2i** in $CDCl_3$.

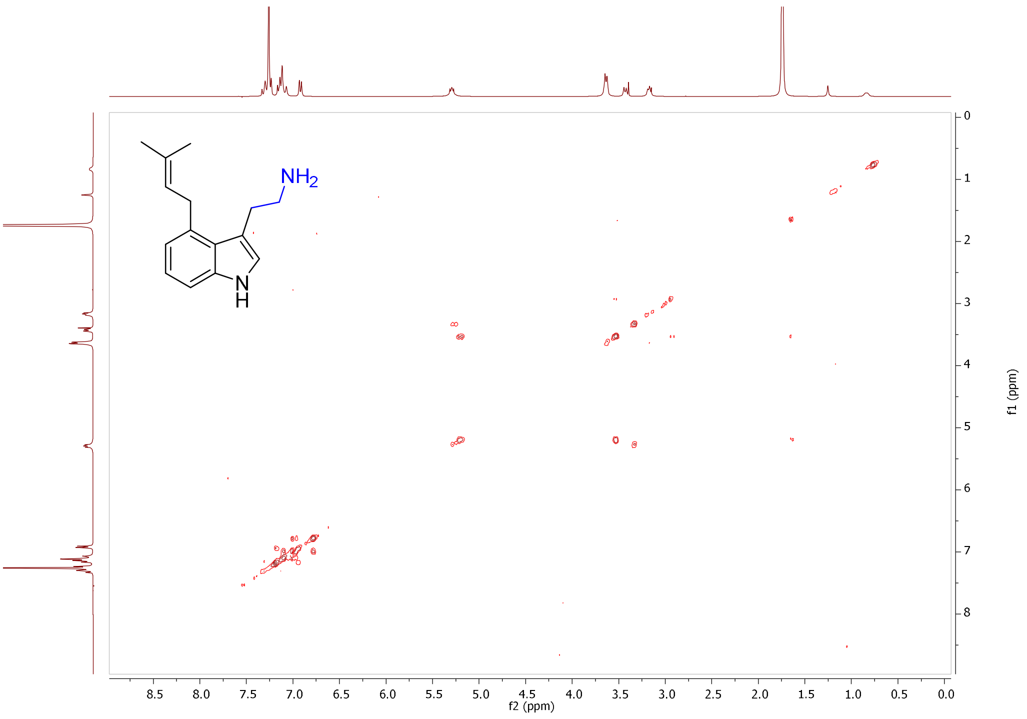

Figure S57: COSY NMR (300 MHz) spectrum of isolated compound **2i** in $CDCl_3$.

OrbiTrap chromatograms and spectra

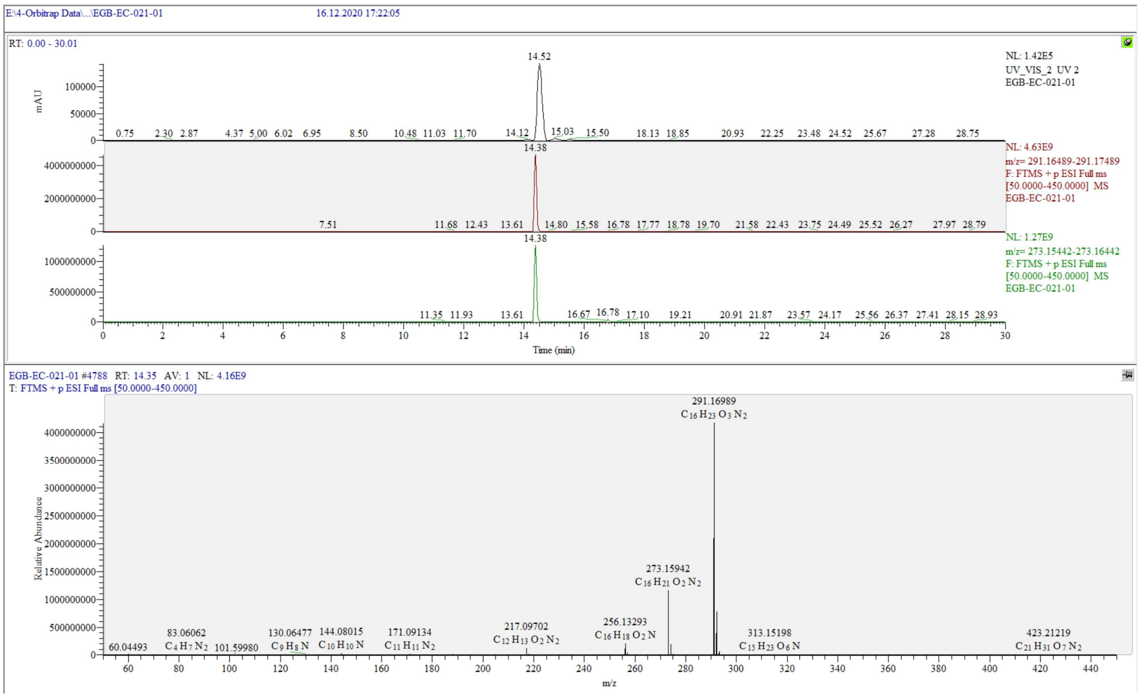

Figure S58: OrbiTrap measurement of reaction sample representing DMAT L-**2a** with retention time 14.35 min, showing the ESI-Full MS spectrum.

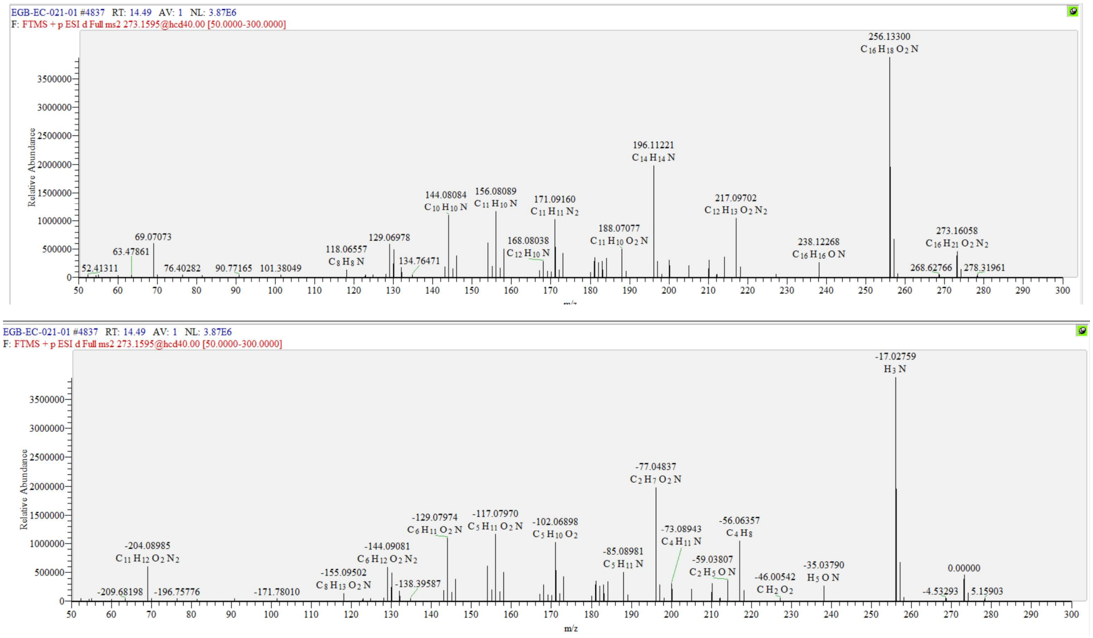

Figure S59: MS/MS spectra of mass 273.16058 at retention time 14.49 min, presenting the fragmentation mechanism of L-**2a**.

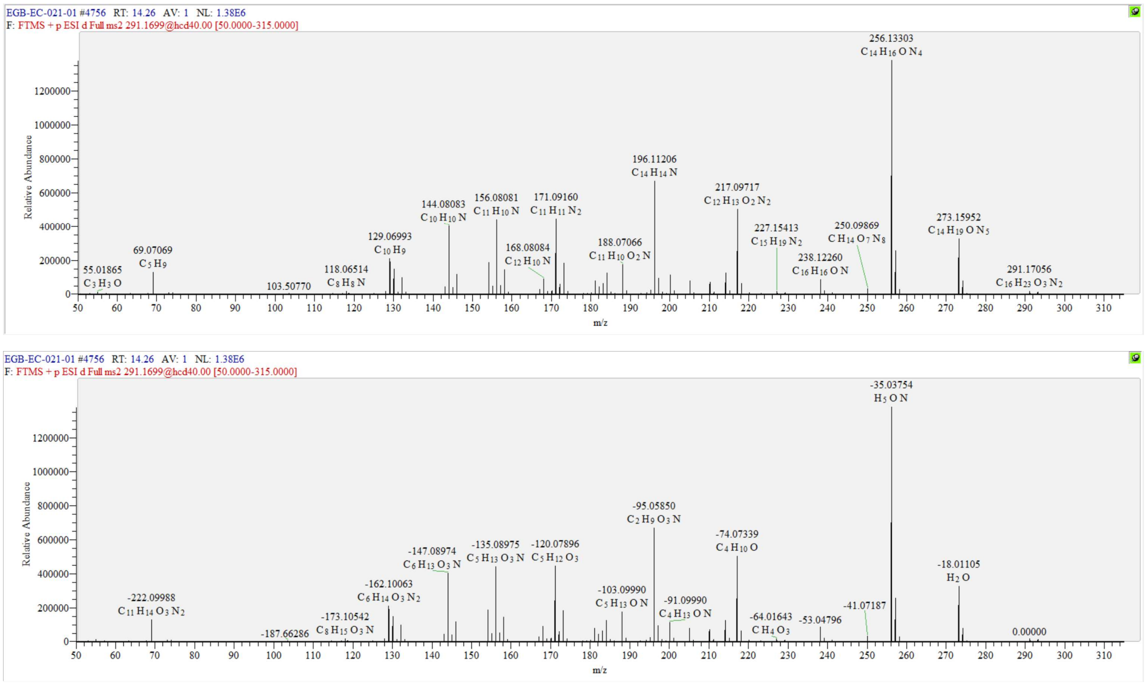

Figure S60: MS/MS spectra of mass 291.16489 at retention time 14.26 min, presenting the fragmentation mechanism of L-**2a**.

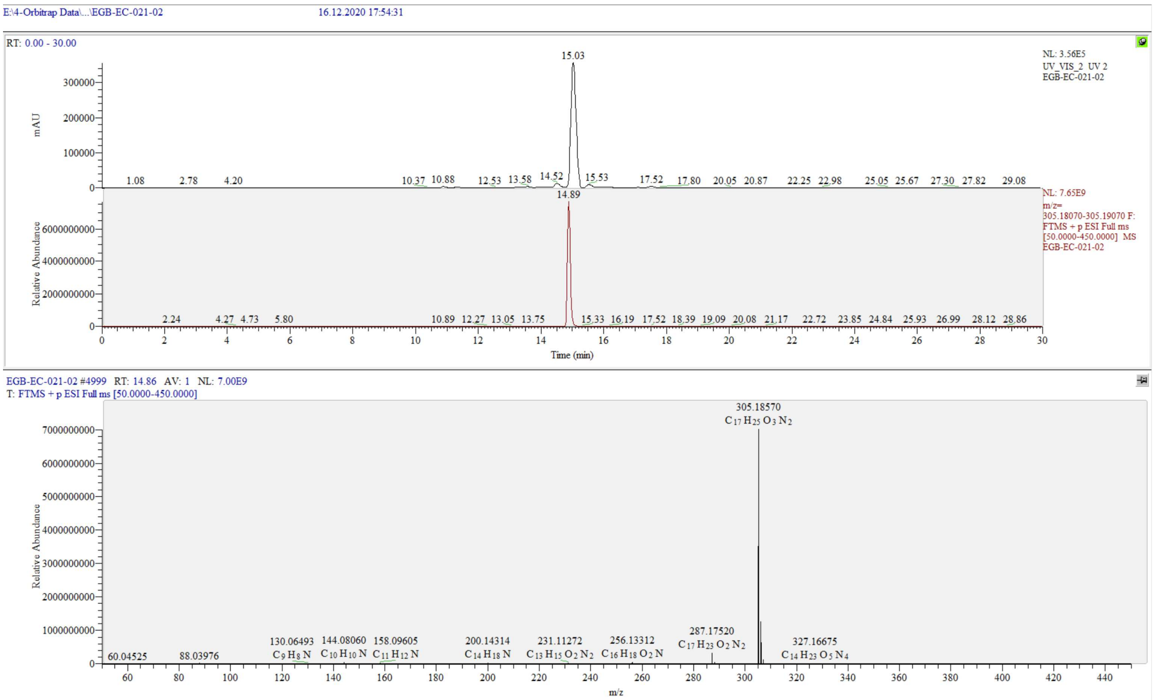

Figure S61: OrbiTrap measurement of reaction sample representing L-**2a** at the retention time 14.86 min, showing the ESI-Full MS spectrum.

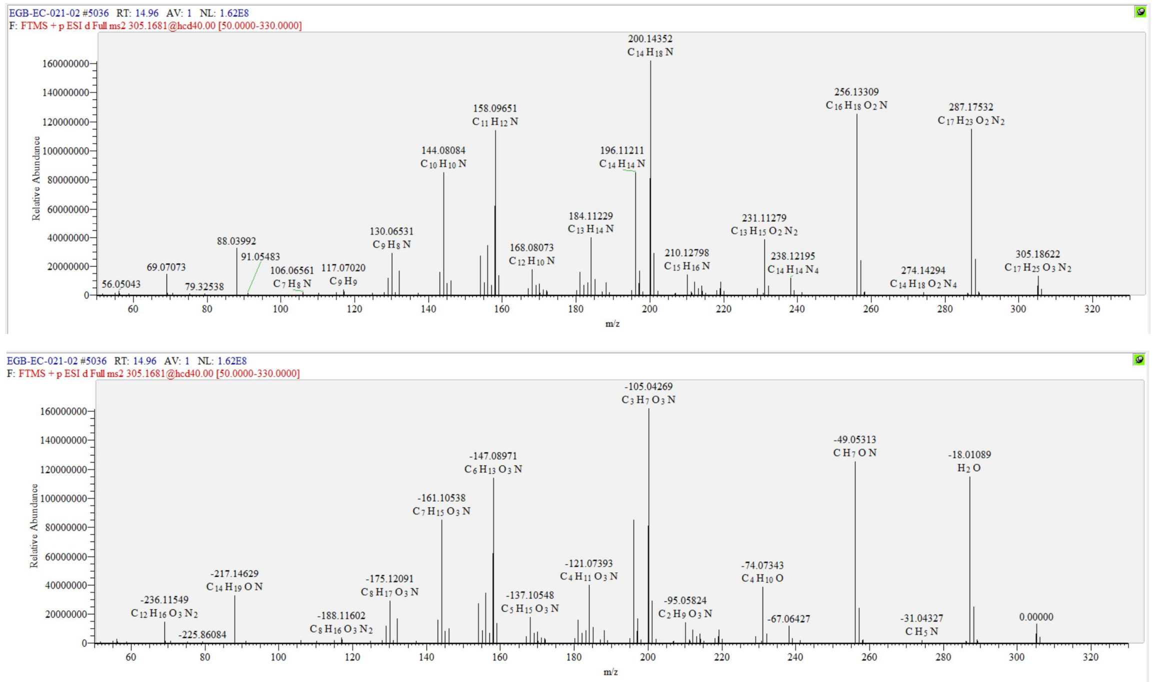

Figure S62: MS/MS spectra of mass 305.18570 at retention time 14.96 min, presenting the fragmentation mechanism of L-**2a**.

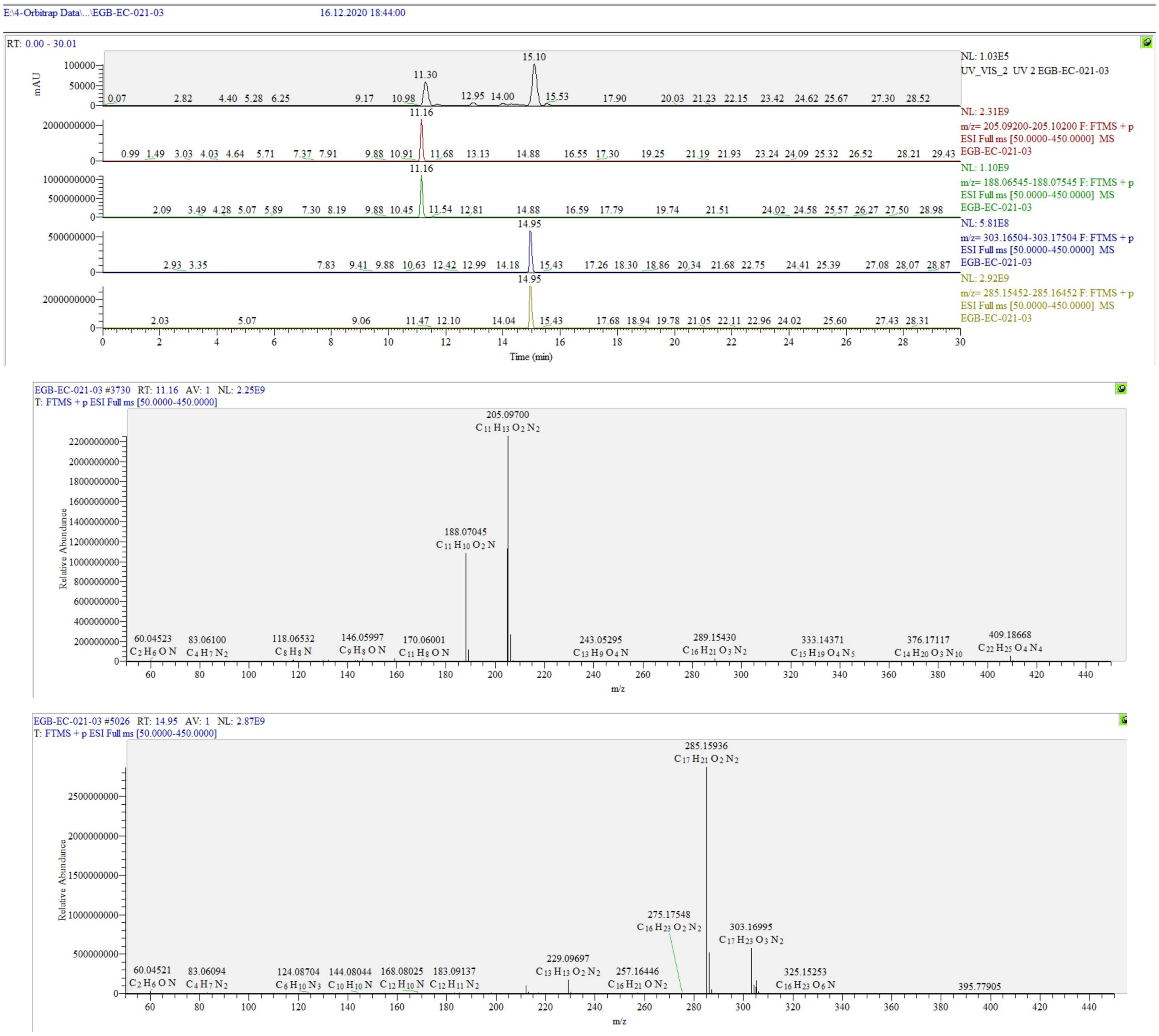

Figure S63: OrbiTrap measurement of reaction sample representing the reaction mixture of L-**1f** prenylation by DmaW, showing the ESI-Full MS spectra at retention time 11.16 min and 15.00 min.

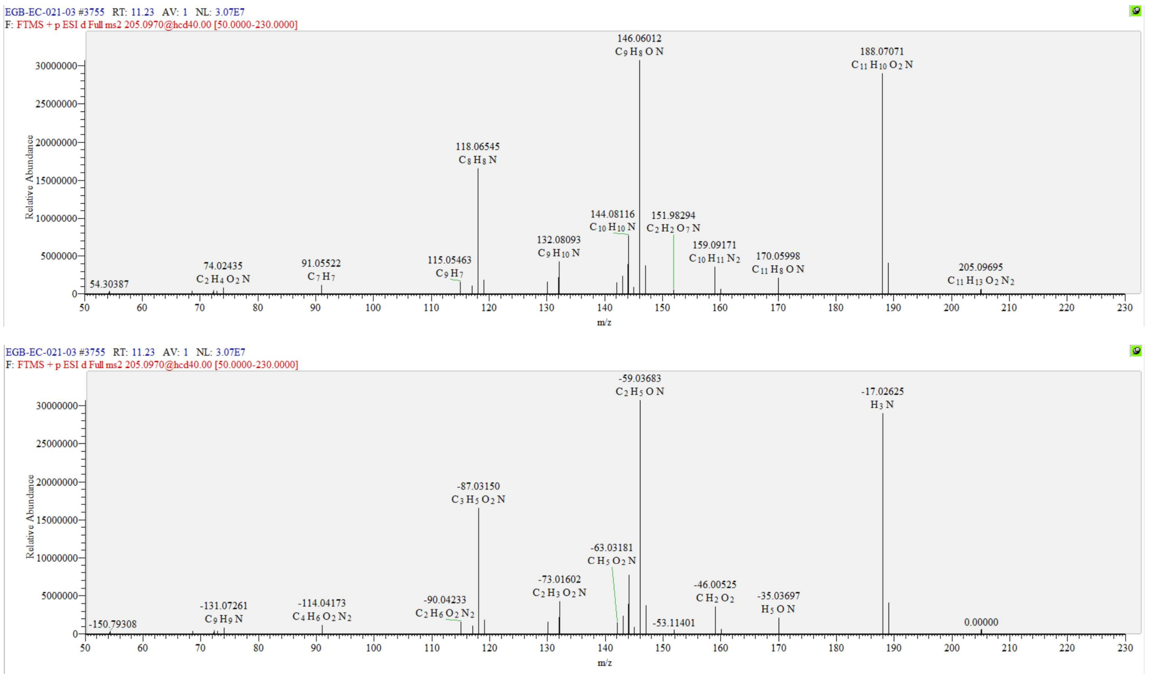

Figure S64: MS/MS spectra of mass 205.09700 at retention time 11.20 min, presenting the fragmentation mechanism of L-**2f**.

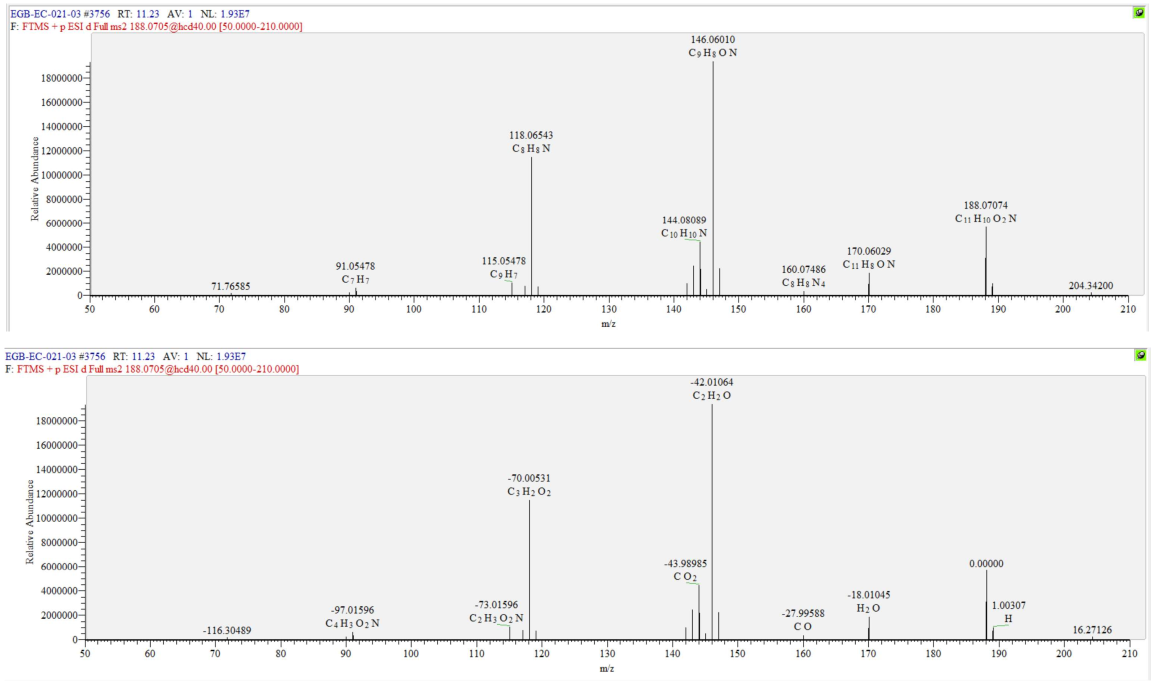

Figure S65: MS/MS spectra of mass 188.07045 at retention time 11.20 min, presenting the fragmentation mechanism of L-**2f**.

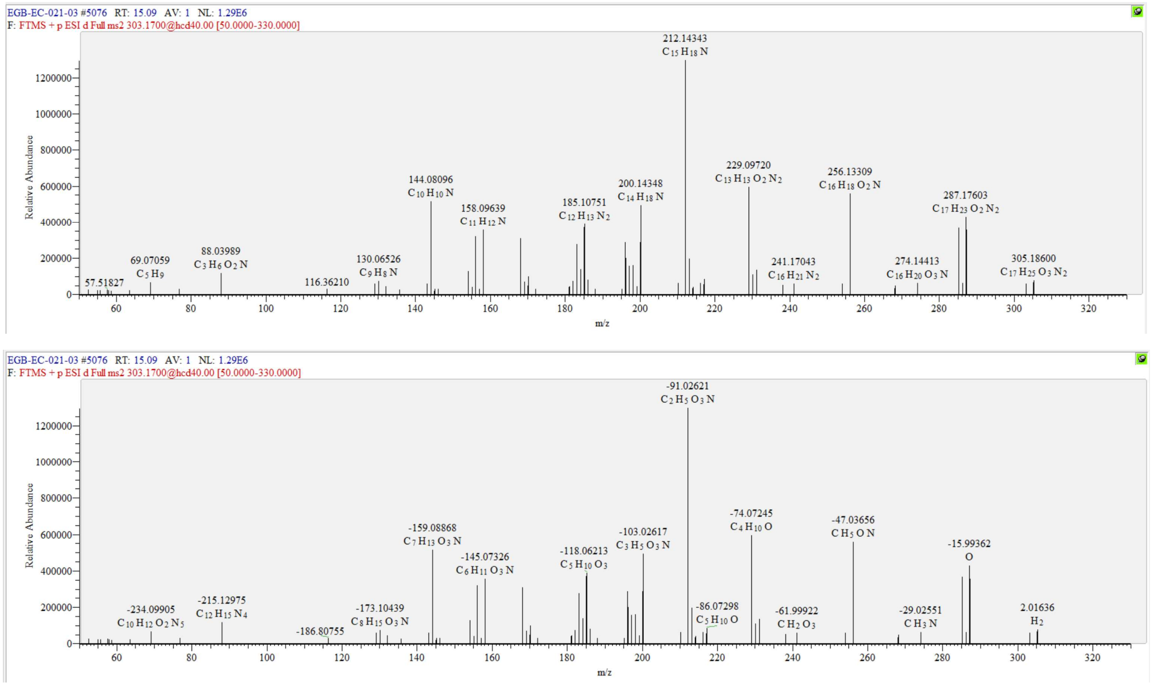

Figure S66: MS/MS spectra of mass 303.17004 at retention time 15.09 min, presenting the fragmentation mechanism of L-**4f**.

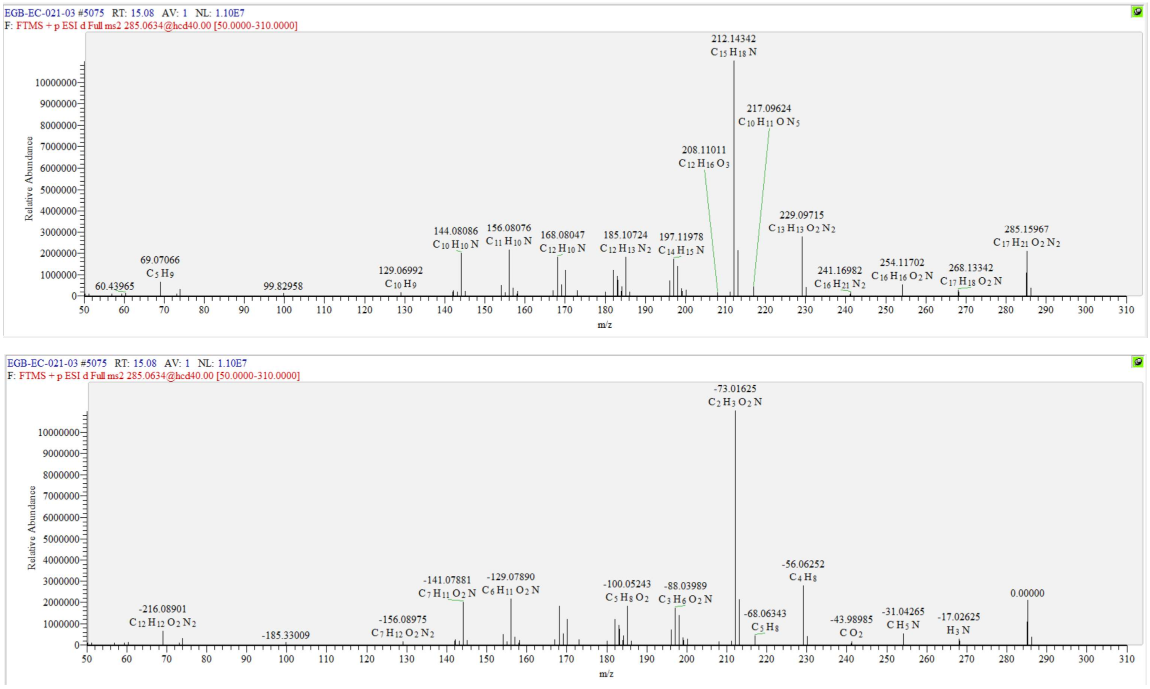

Figure S67: MS/MS spectra of mass 285.15952 at retention time 15.08 min, presenting the fragmentation mechanism of L-**4f**.

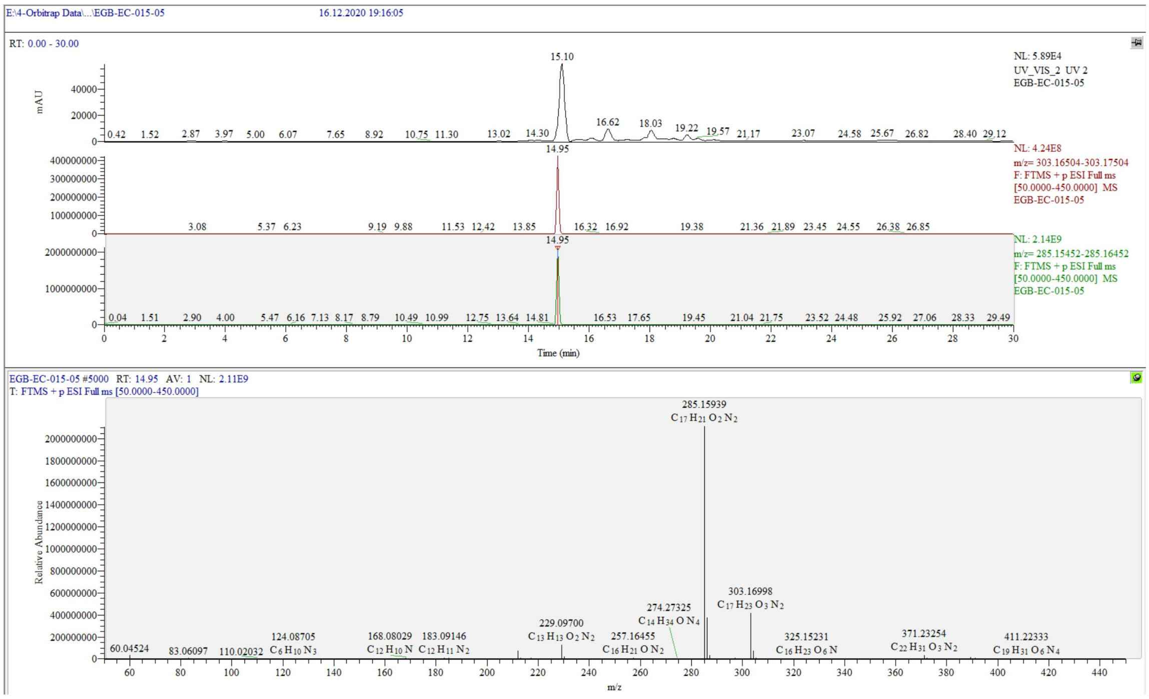

Figure S68: OrbiTrap measurement of reaction sample representing the reaction mixture of **1f** prenylation by DmaW, showing the ESI-Full MS spectrum at retention time 15.10 min.

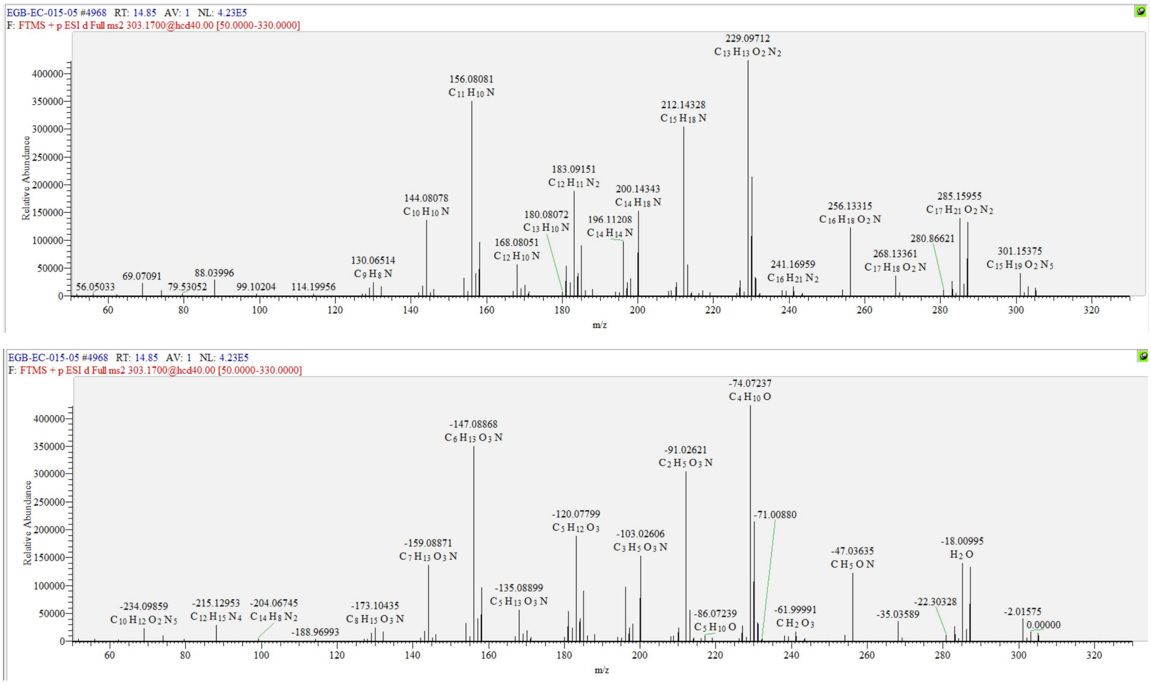

Figure S69: MS/MS spectra of mass 303.17004 at retention time 14.85 min, presenting the fragmentation mechanism of L-**4f**.

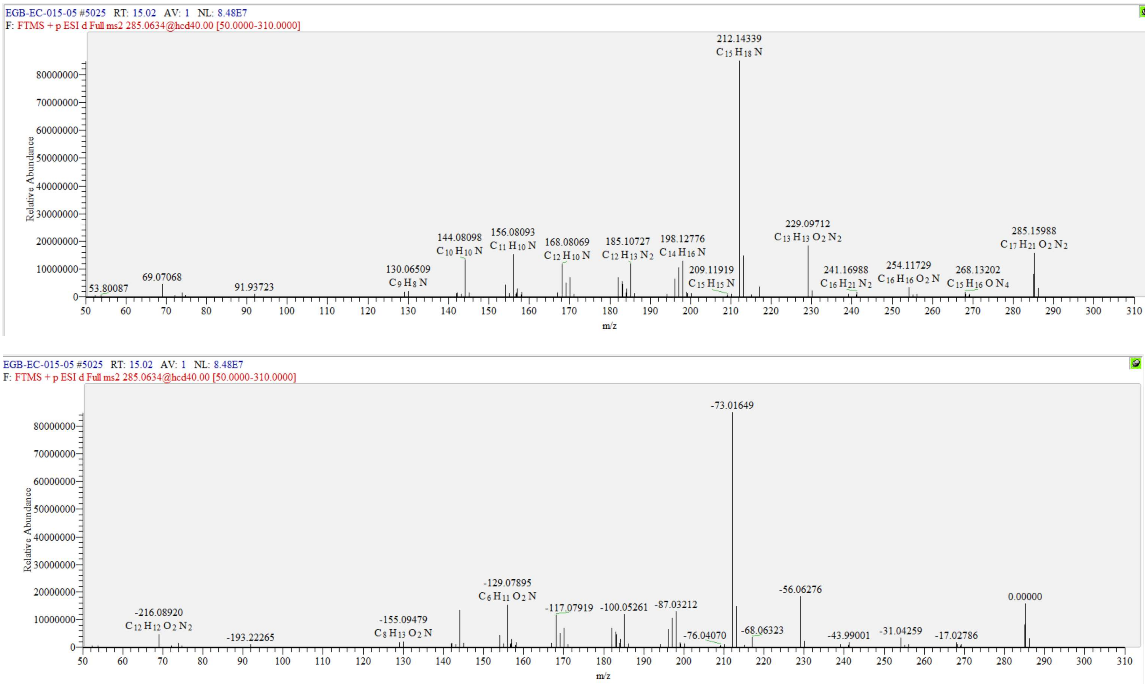

Figure S70: MS/MS spectra of mass 285.15952 at retention time 15.02 min, presenting the fragmentation mechanism of cyclic product L-**4f**.

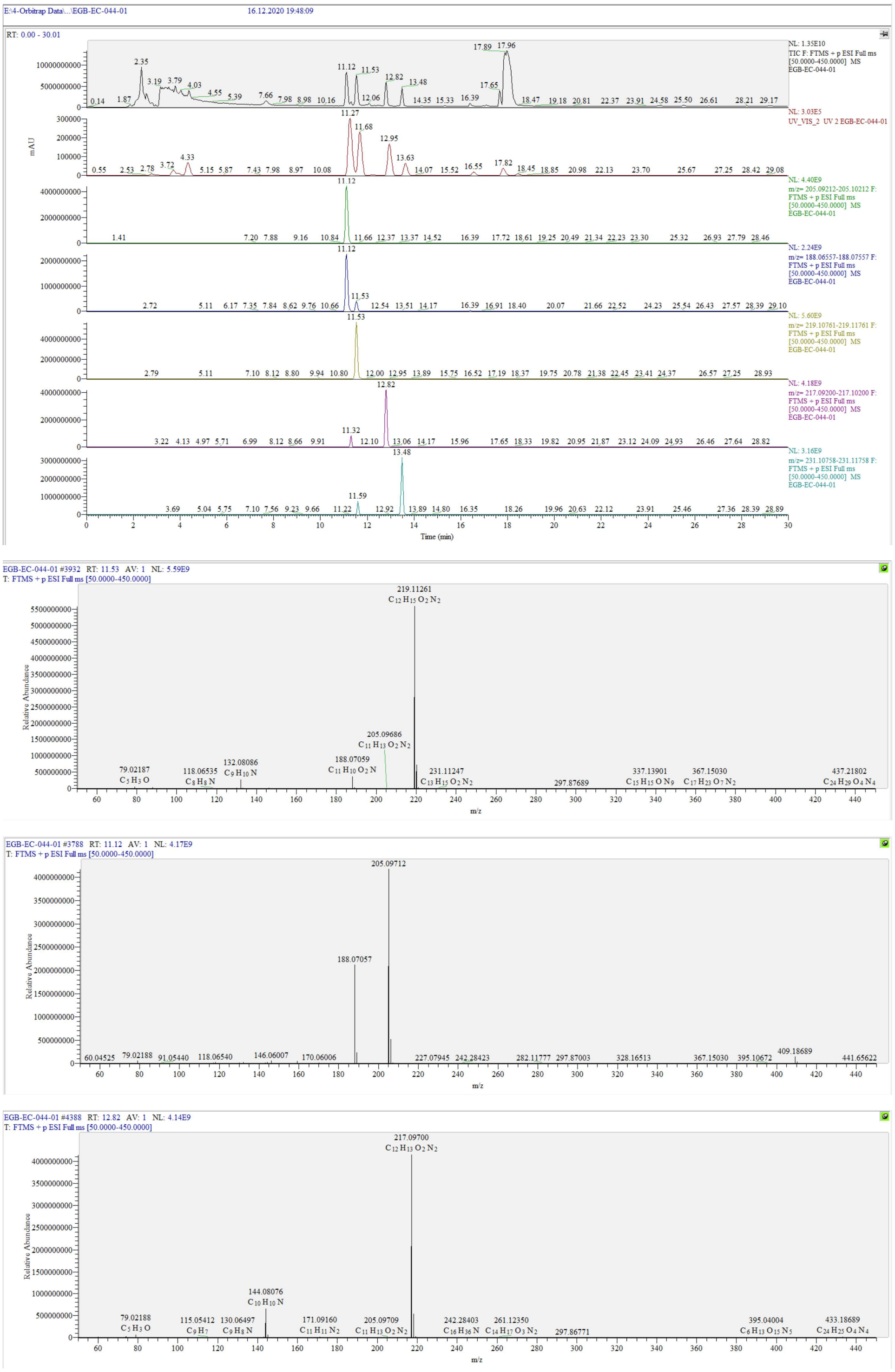

Figure S71: OrbiTrap measurement of reaction sample representing the reaction mixture of **1f** prenylation by DmaW, showing the ESI-Full MS spectra at retention times 11.12, 11.53, 12.82 and 13.48 min, corresponding to L-abrine (**1f**), L-tryptophan (**1a**) and cyclic product **3f**.

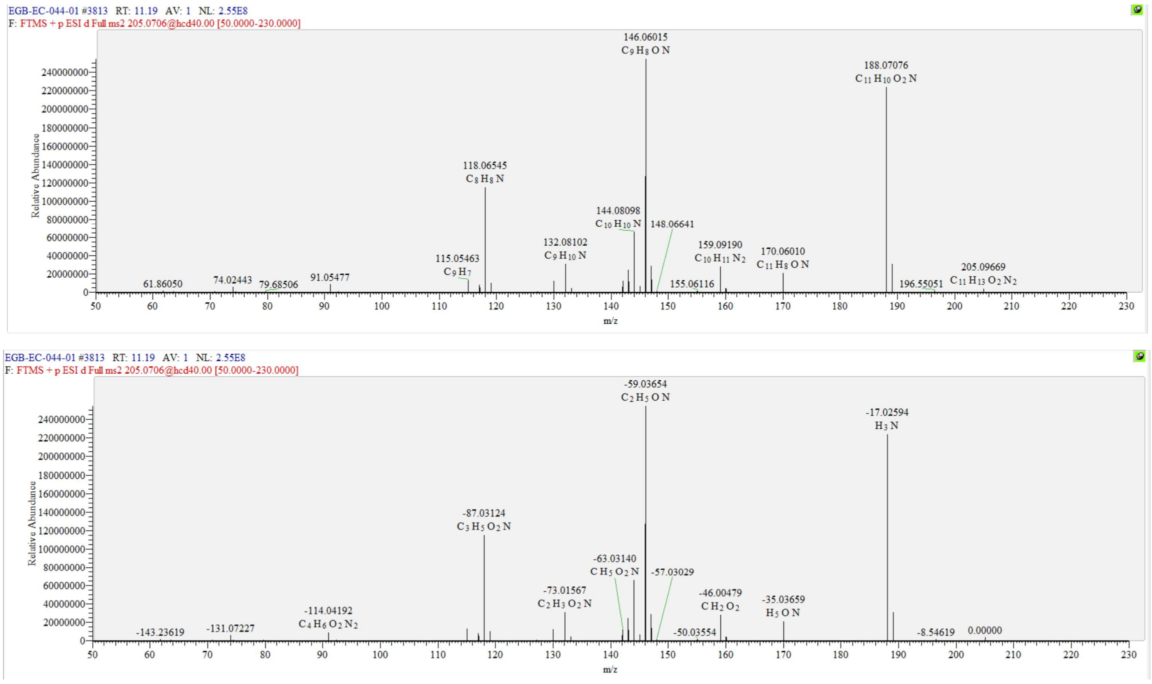

Figure S72: MS/MS spectra of mass 205.09712 at retention time 11.12 min, presenting the fragmentation mechanism of L-**1a**.

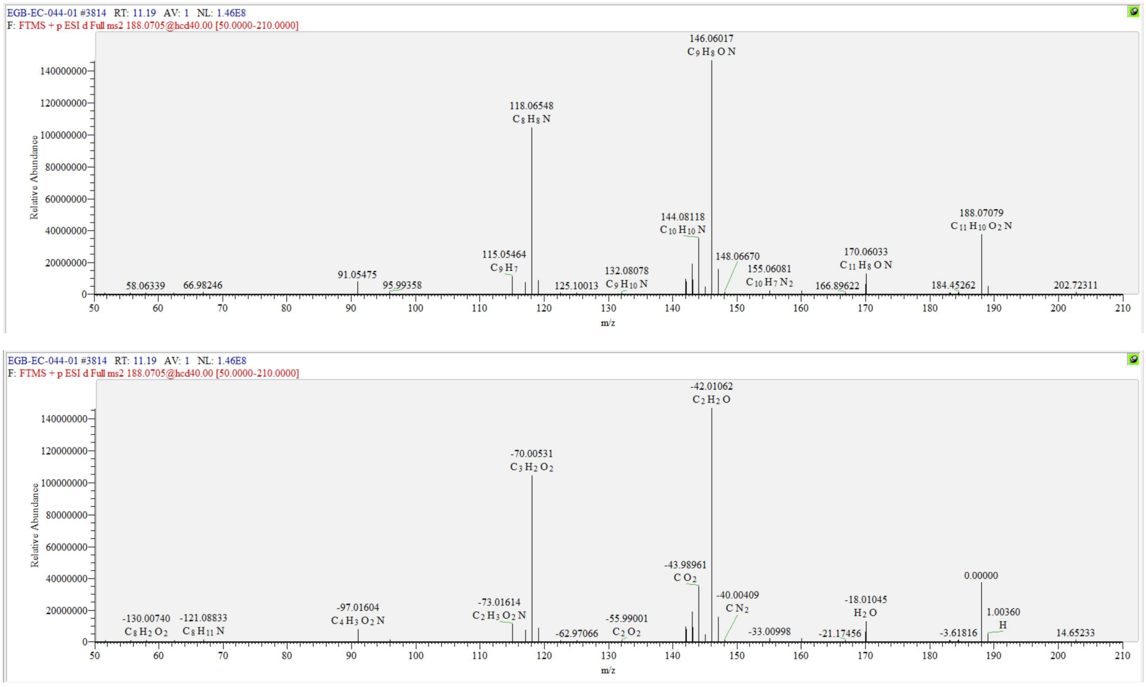

Figure S73: MS/MS spectra of mass 188.07057 at retention time 11.12 min, presenting the fragmentation mechanism of L-**1a**.

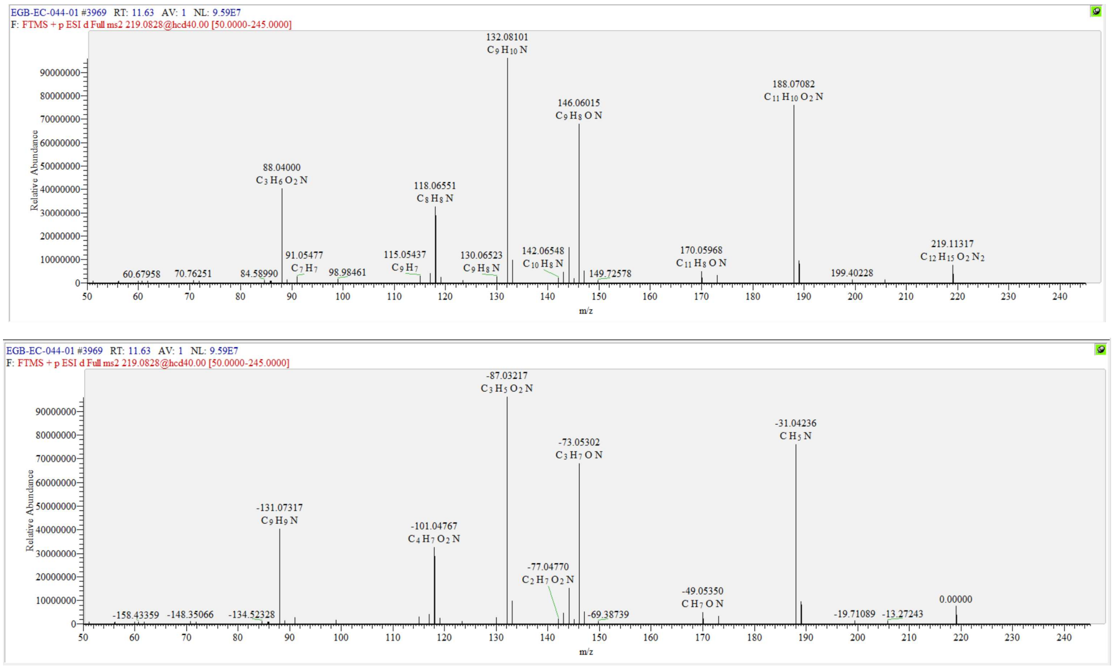

Figure S74: MS/MS spectra of mass 219.11261 at retention time 11.53 min, presenting the fragmentation mechanism of L-**1f**.

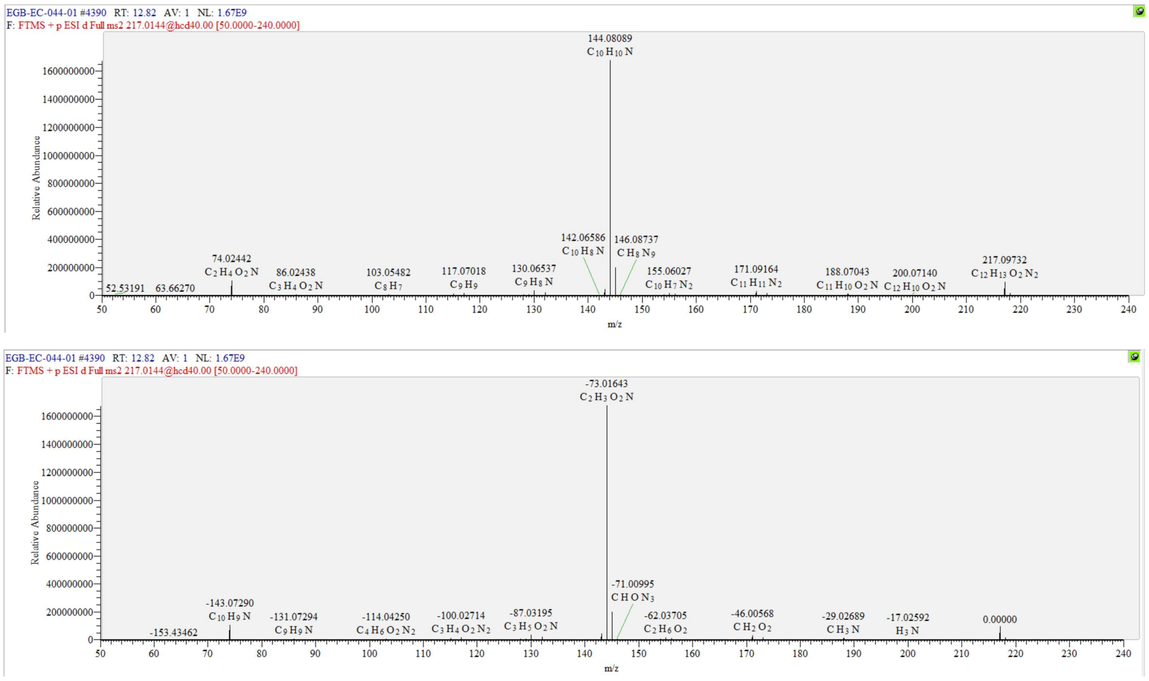

Figure S75: MS/MS spectra of mass 217.09700 at retention time 12.82 min, presenting the fragmentation mechanism of L-**3f**.

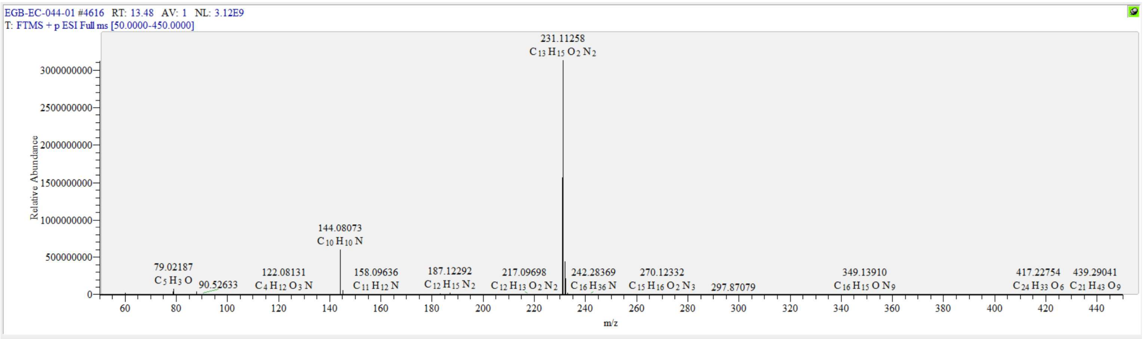

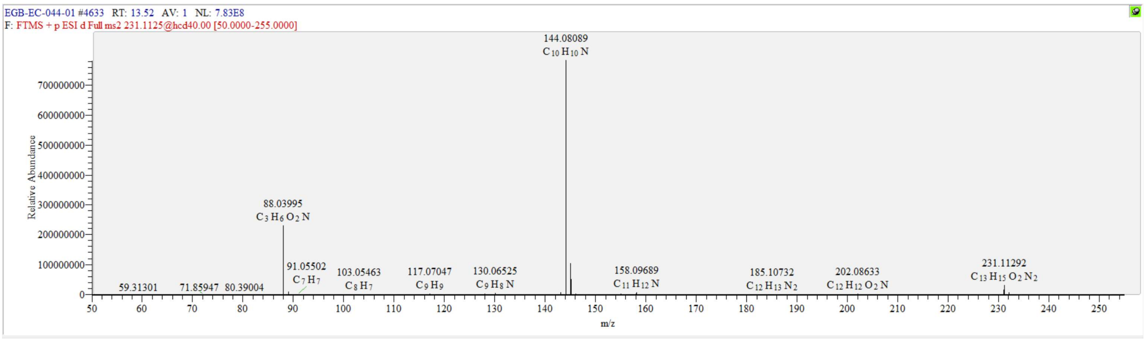

Figure S76: MS/MS spectra of mass 231.117258 at retention time 13.52 min, presenting the fragmentation mechanism of unknown compound.

Reference chromatograms of HPLC-UV

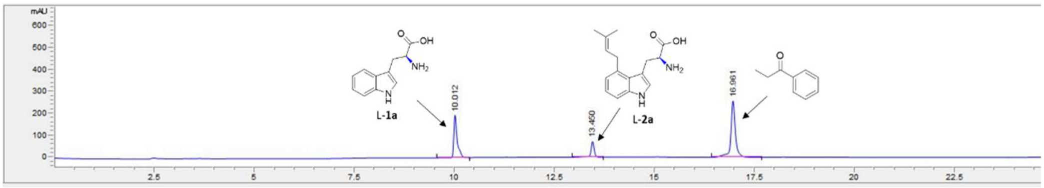

Figure S77: HPLC-UV chromatogram (262 nm) of L-**1a** prenylation by DmaW preparation [CFE of *E. coli* BL21(DE3)] showing substrate L-**1a**, product L-**2a** and internal standard propiophenone.

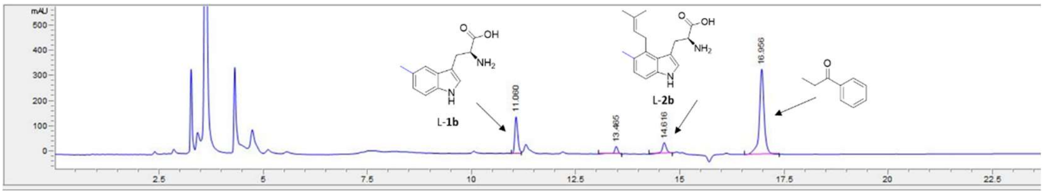

Figure S78: HPLC-UV chromatogram (262 nm) of L-**1b** prenylation by DmaW preparation [CFE of *E. coli* BL21(DE3)] showing substrate L-**1b**, product L-**2b** and internal standard propiophenone.

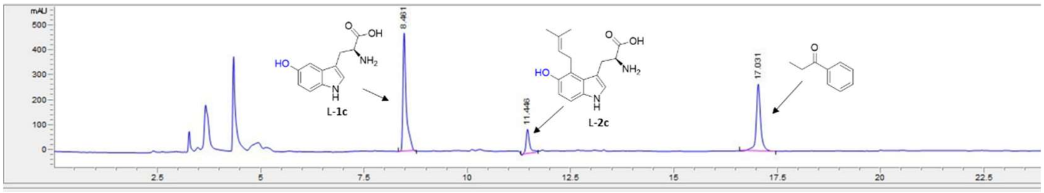

Figure S79: HPLC-UV chromatogram (262 nm) of L-**1c** prenylation by DmaW preparation [CFE of *E. coli* BL21(DE3)] showing substrate L-**1c**, product L-**2c** and internal standard propiophenone.

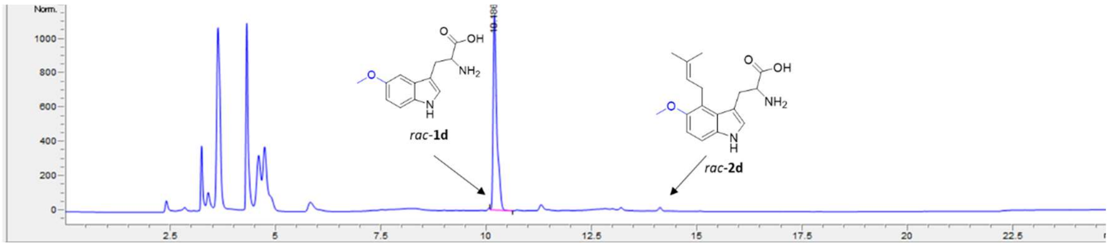

Figure S80: HPLC-UV chromatogram (262 nm) of *rac*-**1d** prenylation by DmaW preparation [CFE of *E. coli* BL21(DE3)] showing substrate *rac*-**1d** and product *rac*-**2d**.

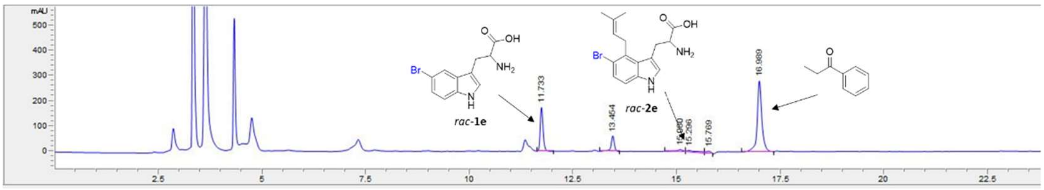

Figure S81: HPLC-UV chromatogram (262 nm) of L-**1e** prenylation by DmaW preparation [CFE of *E. coli* BL21(DE3)] showing substrate L-**1e**, product L-**2e** and internal standard propiophenone.

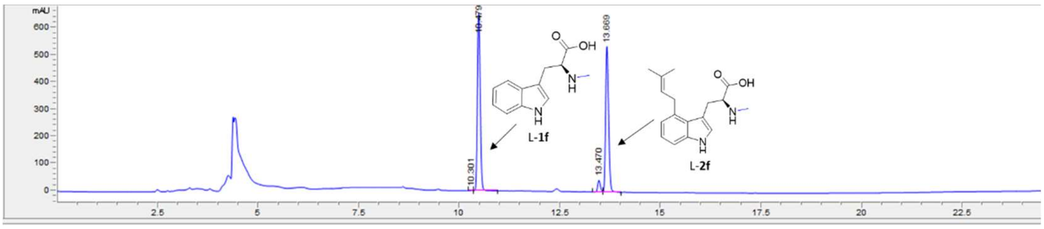

Figure S82: HPLC-UV chromatogram (262 nm) of L-**1f** prenylation by DmaW preparation [CFE of *E. coli* BL21(DE3)] in the absence of molecular oxygen showing substrate L-**1f** and product L-**2f**.

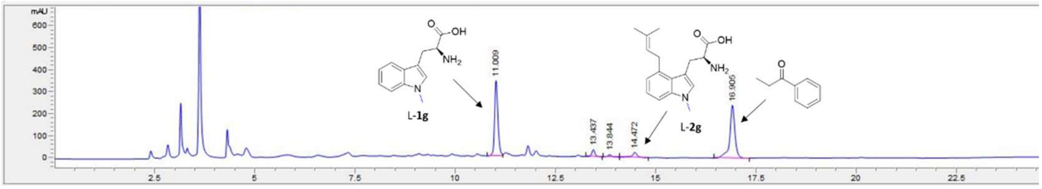

Figure S83: HPLC-UV chromatogram (262 nm) of L-**1g** prenylation by DmaW preparation [CFE of *E. coli* BL21(DE3)] showing substrate L-**1g**, product L-**2g** and internal standard propiophenone.

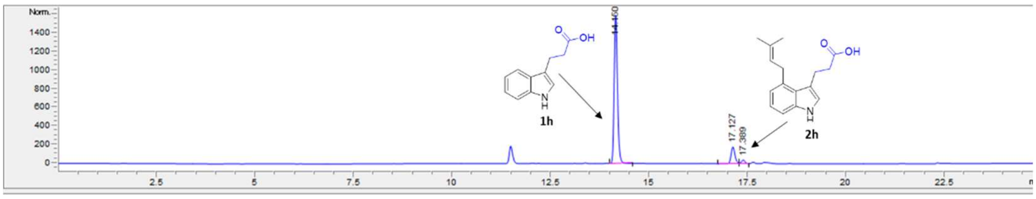

Figure S84: HPLC-UV chromatogram (262 nm) of **1h** prenylation by DmaW preparation [CFE of *E. coli* BL21(DE3)] showing substrate **1h** and product **2h**.

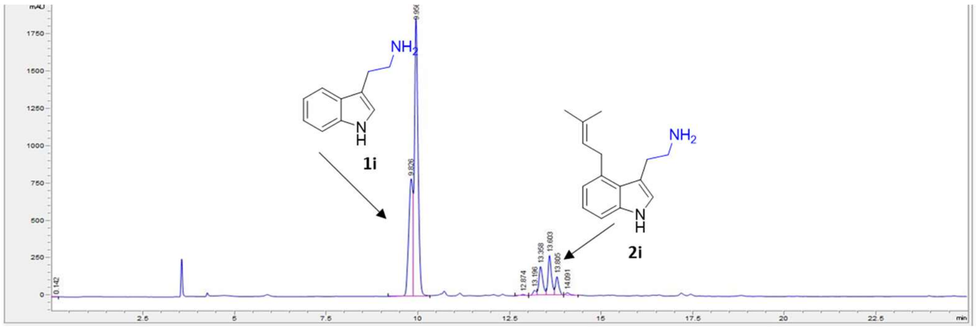

Figure S85: HPLC-UV chromatogram (262 nm) of **1i** prenylation by DmaW preparation [CFE of *E. coli* BL21(DE3)] showing substrate **1i** and product **2i**.

Optical purity of 1f and 1g

1)

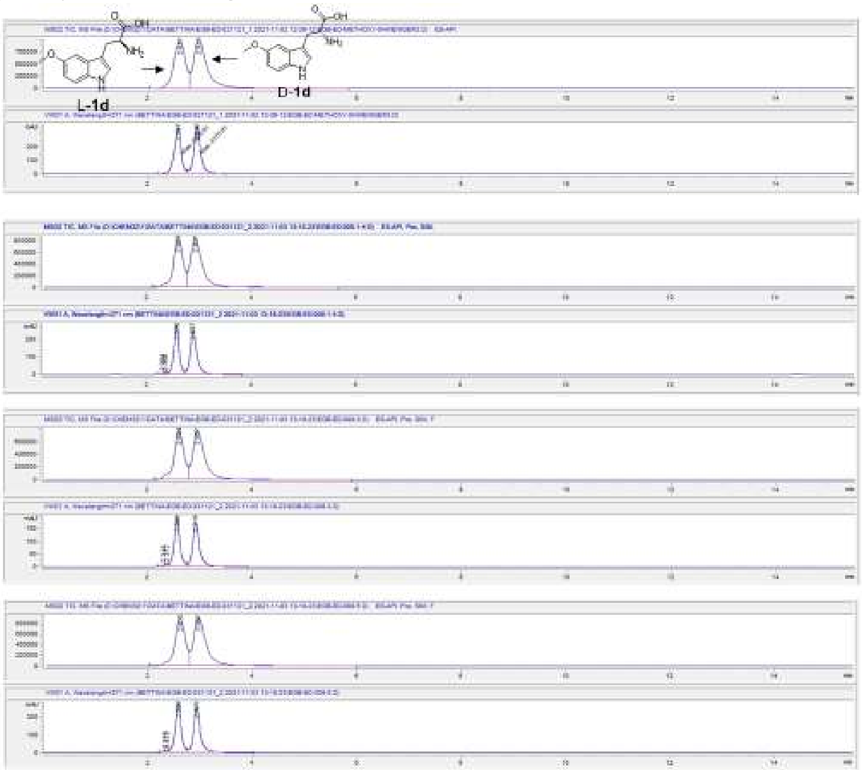

(a) m/z = 235 | UV λ=271 nm

(b) m/z = 235 | UV λ=271 nm

(c) m/z = 235 | UV λ=271 nm

(d) m/z = 235 | UV λ=271 nm

2)

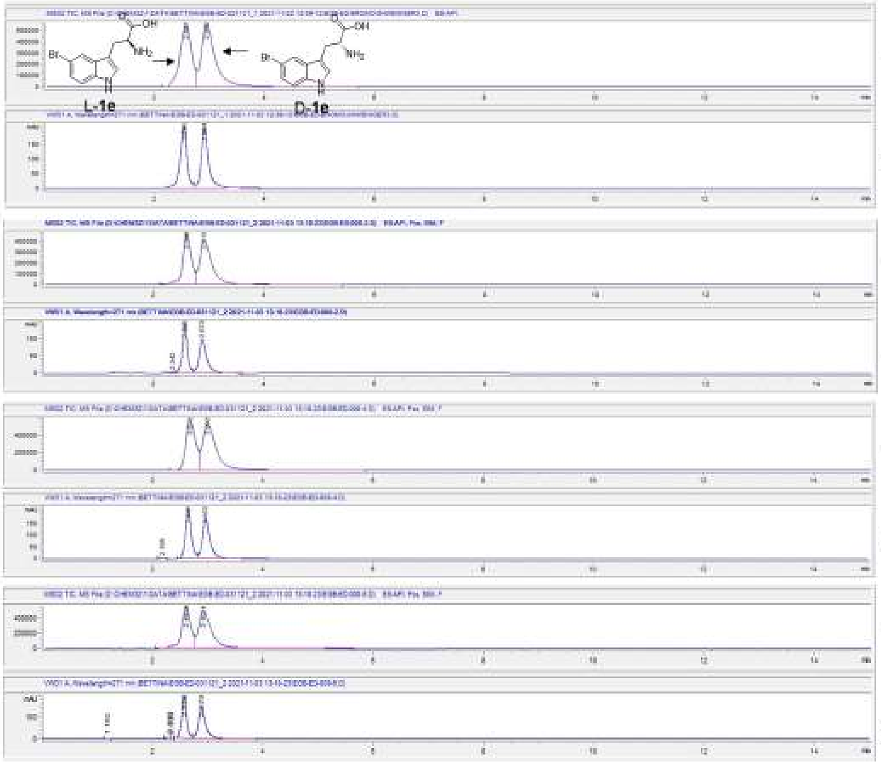

(a) m/z = 284 | UV λ=271 nm

(b) m/z = 284 | UV λ=271 nm

(c) m/z = 284 | UV λ=271 nm

(d) m/z = 284 | UV λ=271 nm

Figure S86: Chiral analysis showing HPLC-MS and UV chromatograms (271 nm) of 1) *rac*-**1d** prenylation by DMATS from *A. japonicus* (b), *C. purpurea* (c) and *T. benhamiae* (d) preparation [CFE of *E. coli* BL21(DE3)] showing the isolated, remaining two enantiomers L and D of substrate after 24 h. (a) shows *rac*-**1d** reference. The MS chromatograms highlight the substrate **1d** with an *m/z* = 235; 2) *rac*-**1e** prenylation by DMATS from *A. japonicus* (b), *C. purpurea* (c) and *T. benhamiae* (d) preparation [CFE of *E. coli* BL21(DE3)] showing the isolated, remaining two enantiomers L and D of substrate after 24 h. (a) shows *rac*-**1e** reference. The MS chromatograms highlight the substrate **1e** with an *m/z* = 284. In all the three chromatograms the L-enantiomer is the earlier peak, and the D-enantiomer could be determined as the second peak (t_r = 3.2 min) based on measurement of a mixture of 2:1 ratio of L-**1a** and D-**1a** with the chiral column.

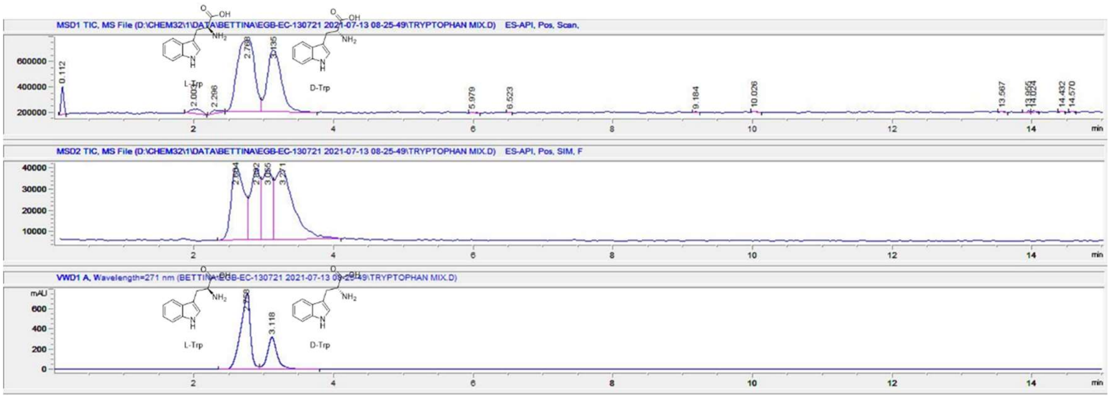

Figure S87: HPLC-MS and UV chromatograms (271 nm) of L-**1a** and D-**1a** in 2:1 ratio, showing L-**1a** to be the earlier eluting enantiomer.

Structural Alignment and Docking Results

For protein engineering, the tryptophan derivatives were docked into the active site of DMATS from *A. fumigatus* together with DMAPP. The experimentally determined structure of the DMATS from *A. fumigatus* was used because (i) a predicted structure of the DMATS from *A. japonicus* using AlphaFold2[6] aligned very nicely to the published crystal structure of *A. fumigatus* harboring L-**1a** and the unreactive sulfur-analog of DMAPP, DMSPP (PDB code: 3I4X, Figure S88) and (ii) using this approach, the need of a stepwise docking of two substrates into the active site could be avoided.

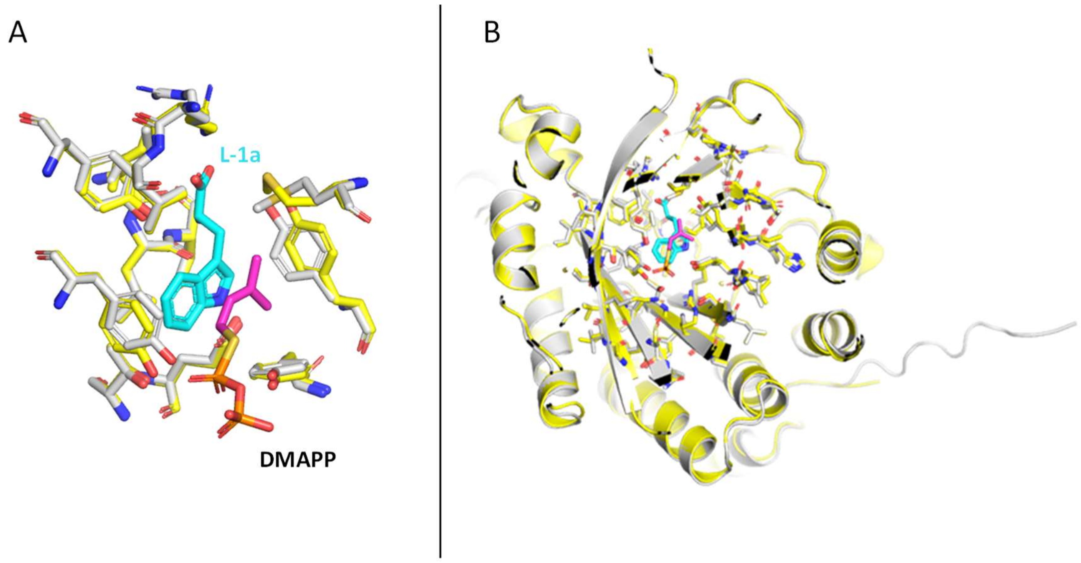

Figure S88: Structural alignment of the predicted structure of the DMATS from *Aspergillus japonicus* (grey) and the experimentally solved structure of the DMATS from *Aspergillus fumigatus* (yellow) with **L-1a** (turquoise) and DMATS in the active site. (A) active site residues within 5 angstroms of **L-1a. (**B) monomer of both DMATSs.

Details about the docking procedure in YASARA and structure analysis in PyMOL are provided in the main article. Structural alignments of the best docking poses of D-**1a** and L-**1g** and the natural substrate L-**1a** in the DMATS is given below.

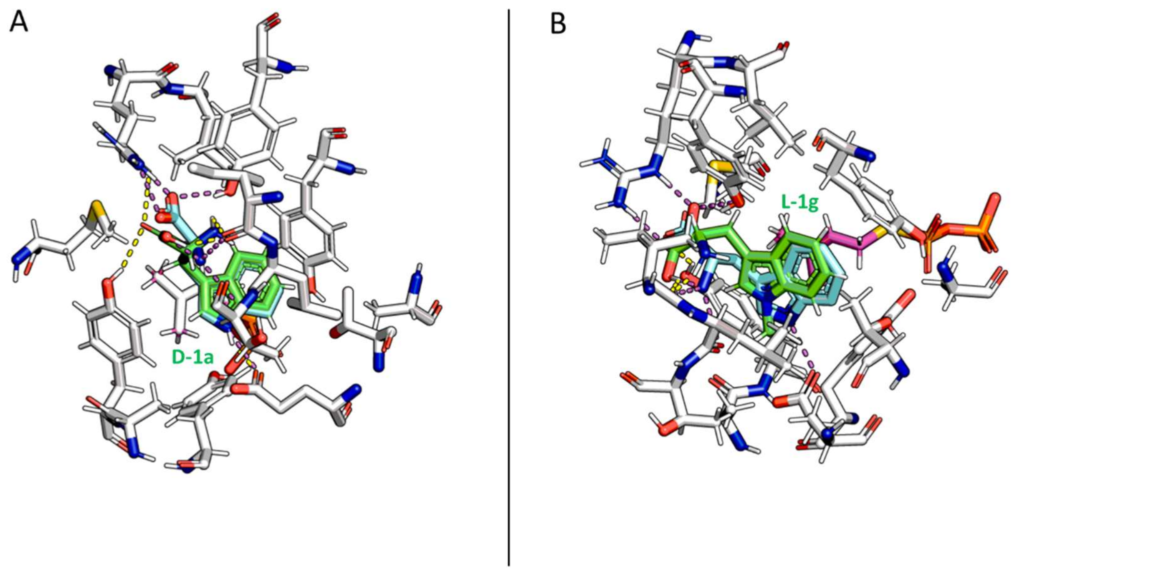

Figure S89: Structural alignment of L-**1a** (turquoise) and (A) D-**1a** / (B) L-**1g** (green) docking results into DMATS from *A. fumigatus* (See "Experimental Section"). Polar interactions of L-**1a** are highlighted in magenta and polar interactions of tryptophan derivatives in yellow.

References

[1] A. Dummler, A. M. Lawrence, A. de Marco, *Microb. Cell. Fact.* **2005**, *4*, 34.

[2] C. A. Nielsen, C. Folly, A. Hatsch, A. Molt, H. Schroder, S. E. O'Connor, M. Naesby, *Microb. Cell Fact.* **2014**, *13*, 95.

[3] H. F. Tsai, H. Wang, J. C. Gebler, C. D. Poulter, C. L. Schardl, *Biochem. Biophys. Res. Comm.* **1995**, *216*, 119-125.

[4] P. Zhang, W. Chan, I. L. Ang, R. Wei, M. M. T. Lam, K. M. K. Lei, T. C. W. Poon, *Sci Rep.* **2019**, *9*, 6453.

[5] X. Wang, X. J. Zhong, N. Zhou, N. Cai, J. H. Xu, Q. B. Wang, J. J. Li, Q. Liu, P. C. Lin, X. Y. Shang, *Molecules* **2020**, *25*.

[6] J. Jumper, R. Evans, A. Pritzel, T. Green, M. Figurnov, O. Ronneberger, K. Tunyasuvunakool, R. Bates, A. Zidek, A. Potapenko, A. Bridgland, C. Meyer, S. A. A. Kohl, A. J. Ballard, A. Cowie, B. Romera-Paredes, S. Nikolov, R. Jain, J. Adler, T. Back, S. Petersen, D. Reiman, E. Clancy, M. Zielinski, M. Steinegger, M. Pacholska, T. Berghammer, S. Bodenstein, D. Silver, O. Vinyals, A. W. Senior, K. Kavukcuoglu, P. Kohli, D. Hassabis, *Nature* **2021**, *596*, 583-589.
